# Reference Standards for Newborn Screening of Metabolic Disorders by Tandem Mass Spectrometry: A Nationwide Study on Millions of Chinese Neonatal Populations

**DOI:** 10.3389/fmolb.2021.719866

**Published:** 2021-12-16

**Authors:** Falin He, Rulai Yang, Xinwen Huang, Yaping Tian, Xiaofang Pei, Mary Kathryn Bohn, Lin Zou, Yan Wang, Haibo Li, Ting Wang, Maosheng Gu, Tao Jiang, Xigui Chen, Hui Zou, Hongwei Wei, Weibing Tian, Tian Tang, Khosrow Adeli, Zhiguo Wang

**Affiliations:** ^1^ National Center for Clinical Laboratories, Beijing Engineering Research Center of Laboratory Medicine, Beijing Hospital, National Center of Gerontology, Institute of Geriatric Medicine, Chinese Academy of Medical Sciences, Beijing, China; ^2^ Department of Genetics and Metabolism, Children’s Hospital of Zhejiang University School of Medicine, National Clinical Research Center for Child Health, Hangzhou, China; ^3^ Chinese PLA General Hospital and Medical School of Chinese PLA, Beijing, China; ^4^ Department of Laboratory Sciences, West China School of Public Health and West China Fourth Hospital, Sichuan University, Chengdu, China; ^5^ Department of Pediatric Laboratory Medicine, CALIPER Program, The Hospital for Sick Children, Toronto, ON, Canada; ^6^ The Children’s Hospital of Chongqing Medical University, Chongqing, China; ^7^ Ningbo Women and Children’s Hospital, Ningbo, China; ^8^ Suzhou Municipal Hospital, Suzhou, China; ^9^ The Xuzhou Maternity and Child Health Care Hospital, Xuzhou, China; ^10^ Nanjing Maternity and Child Health Care Hospital, Nanjing, China; ^11^ Jining Maternal and Child Health Family Planning Service Center, Jining, China; ^12^ Jinan Maternity and Child Care Hospital Affiliated to Shandong First Medical University, Jinan, China; ^13^ Linyi Maternity and Child Health Care Hospital, Linyi, China; ^14^ W. F. Maternal and Child Health Hospital, Weifang, China

**Keywords:** newborn screening, inborn errors of metabolism, mass spectrometry, reference intervals, biomarkers

## Abstract

**Introduction:** The major clinical problem presently confronting the Chinese newborn screening (NBS) programs by tandem mass spectrometry (MS/MS) is the lack of comprehensive reference intervals (RIs) for disease biomarkers. To close this gap, the Chinese National Center for Clinical Laboratories (NCCL) launched a nationwide study to investigate the dynamic pattern of 35 MS/MS NBS biomarkers and establish accurate and robust RIs.

**Methods:** Blood spot samples from 4,714,089 Chinese neonates were tested in participating centers/laboratories and used for study analysis. MS/MS NBS biomarker trends were visually assessed by their concentrations over age. Specific partitions were determined arbitrarily by each day and sex or by the statistical method of Harris and Boyd. RIs, corresponding to the 2.5th and 97.5th percentiles, as well as the 1th, 25th, 75th and 99th percentiles were calculated for each reference partition using a non-parametric rank approach.

**Results:** Most MS/MS NBS biomarkers fluctuated during the first week of life, followed by a relatively stable concentration. Age and sex-specific RIs were established and presented an improved specificity over the RIs used in participating centers/laboratories. Females demonstrated higher 2.5th and 97.5th percentiles in all amino acids except arginine and ornithine than males, whereas males showed higher 2.5th and 97.5th percentiles in most acylcarnitines.

**Conclusion:** The present study determined the dynamic trends of 35 MS/MS biomarkers and established age and sex-specific RIs, valuably contributing to the current literature and timely evaluation of neonatal health and disease.

## Introduction

The introduction of tandem mass spectrometry (MS/MS) has been a tremendous advance in newborn screening (NBS) programs. By assessing the concentrations of clinically important biomarkers in neonatal dried blood spot (DBS) samples, MS/MS is capable of recognizing dozens of inborn errors of metabolism, including amino acid, organic acid and fatty acid disorders (Wilcken et al., 2003). Consequently, determining whether the levels of disease biomarkers are within the normal or “healthy” range, commonly referred to as a reference interval (RI), is critical for clinical decision-making in early life. Several studies have highlighted serious consequences for patients when inappropriate RIs are used in clinical practice. One report by Cavedon and colleagues indicated that the diagnosis of long-chain acyl-CoA dehydrogenase and trifunctional protein deficiencies could be missed if the neonatal long-chain acylcarnitines test results were interpreted using RIs for older children (Cavedon et al., 2005). Another report suggested that age-adjusted cutoffs for thyroid stimulating hormone are essential to reduce false positive rates of congenital hypothyroidism (CH) (Ogunkeye et al., 2007). These investigations underscore the importance of establishing accurate and robust RIs for disease biomarkers in MS/MS NBS programs.

The region four stork (R4S) collaborative project has made great advances in establishing comprehensive RIs for disease biomarkers in MS/MS NBS programs (McHugh et al., 2011). However, recent evidence suggested that the RIs provided by R4S were not entirely suitable for the Chinese newborns because several true-positive cases of hyperprolinemia or *β*-ketothiolase deficiency were missed while using the RIs of R4S as reference standards (Zhenzhen et al., 2018). Hence, the establishment of Chinese neonatal population specific RIs of biomarkers becomes a priority for the nationwide MS/MS NBS programs.

The Chinese National Center for Clinical Laboratories (NCCL) is the National Newborn Screening Laboratory Quality Control Center of China, which has been authorized to evaluate the neonatal screening quality among different laboratories since 1998 (Zhan et al., 2009). In early 2014, the NCCL initiated a large-scale NBS survey where 114 newborn screening laboratories/centers distributed in 29 provinces or municipalities participated. The resultant and unpublished data demonstrated that the criteria of sampling age among different MS/MS NBS centers/laboratories varied significantly. In addition, 56 of the 114 (49.1%) MS/MS NBS facilities adopted default RIs from manufacturers. The remaining laboratories developed their own RIs and their “new standard” was derived based on a limited number of samples, which did not reflect the general healthy population. To address these critical gaps, the NCCL launched a national newborn health care initiative, namely, Nationwide Newborn Screening Cooperative Program (NNSCP). The specific objectives of NNSCP were: 1) to investigate the baseline level of disease biomarkers in healthy Chinese newborns; 2) to monitor the dynamic change of biomarkers through the neonatal age range; 3) to develop a formal guideline on sampling age for MS/MS NBS programs; 4) to establish Chinese neonatal RIs for disease biomarkers; and 6) to address whether the variance in demographics (e.g. sex, age, ethnicity), environment (e.g. climate, seasonal changes) and/or diet preferences influenced the concentration of biomarkers. Since its inception in 2014, NNSCP has become a National Key Research and Development Program of China. As of December 31, 2019, more than 7.2 million neonates participated in this initiative, contributing a total of 6,447,276 DBS samples. In the present study, we report age- and sex-specific variations observed in NNSCP and establish robust RIs for 35 MS/MS NBS biomarkers, including 11 amino acids and 24 acylcarnitines.

## Methods

### Participating Study Centers

To recruit eligible MS/MS NBS centers/laboratories for the NNSCP, strict criteria was applied. Only MS/MS NBS centers/laboratories that passed three consecutive external quality assessments (EQA) by NCCL could join the program. The details of the NCCL EQA procedure include the following: the NCCL distributed a batch of quality control products containing low, median and high concentration of certain target analytes to each enrolled NBS center/laboratory; study centers were required to test all quality control products and return the results to the NCCL; the acceptable performance for any test analytes was ±3 standard deviation (SD) from the median value of the peer group laboratories. Reference value data for the current study were not collected from participating centers/laboratories until they passed quality requirements. All participating centers/laboratories adopted the uniform screening panel covering 39 conditions. The details of disorders screened in participating centers/laboratories are reported in Table 1.

**TABLE 1:** The newborn screening panel in the MS/MS NBS participating centers/laboratories of NNSCP.

Categories	Conditions
Amino acid disorders	maple syrup urine disease
	tyrosinemia
	hyperphenylalaninemia
	homocysteinemia
	non-ketotic hyperglycinemia
	hypermethioninemia
Organic acid metabolism disorders	methylmalonic acidemia
	propionic acidemia
	glutaric acidemia
	multiple carboxylase deficiency
	3-hydroxy-3-methylglutaric acidemia
	isovaleric acidemia
	3-methylcrotonyl coenzyme A carboxylase deficiency
	malonyl coenzyme A decarboxylase deficiency
	3-methylglutaconic acidemia
	ethylmalonic encephalopathy
	beta-ketothiolase deficiency
	bisobutyryl coenzyme A dehydrogenase deficiency
	2-methylbutyrylglycinuria
	2-methyl-3-hydroxybutyryl coenzyme A dehydrogenase deficiency
Fatty acid oxidation disorders	very long-chain acyl coenzyme A dehydrogenase deficiency
	long-chain 3 hydroxyacyl coenzyme A dehydrogenase deficiency
	medium-chain acyl coenzyme A dehydrogenase deficiency
	short-chain acyl coenzyme A dehydrogenase deficiency
	short-chain 3-hydroxyacyl coenzyme A dehydrogenase deficiency
	multiple acyl coenzyme A deficiency
	carnitine palmitoyltransferase II deficiency
	primary carnitine deficiency
	carnitine palmitoyltransferase I deficiency
	carnitine-acylcarnitine translocase deficiency
Urea cycle disorders	argininemia
	argininosuccinic acidemia
	carbamoyl phosphate synthetase I deficiency
	N-acetylglutamate synthase deficiency
	ornithine transcarbamylase deficiency
	argininosuccinate synthetase deficiency
	citrin deficiency
	hyperornithinemia-hyperammonemia-homocitrullinemia
	ornithine aminotransferase deficiency

### Sample Acquisition and Analysis

Full-term neonates (37 0/7 weeks of gestation through 41 6/7 weeks of gestation) from birth to 14 days of age with birth weight of 2.5–4.0 kg from the NNSCP were selected as the study population. Subjects were excluded in the case of acute/chronic illness, family history of inborn errors, use of medications or receiving parenteral nutrition. A heel prick blood sample was taken from each subject and dripped on a Whatman^TM^ 903 filter paper (GE Healthcare Ltd., Cardiff, UK). After evaporating to dryness in ambient temperature, the DBS samples were sent immediately to the local NNSCP participating centers/laboratories for testing. The time interval between sampling and testing was ≤1 week. The MS/MS platforms for sample analysis included: Waters Acquity TQD-, Xevo TQD- or Quattro micro API-triple quadrupole mass spectrometry; AB Sciex QTRAP 3200-, 4,000- or 4500-hybrid triple quadrupole/linear ion trap mass spectrometry. The allowable bias between the analyte concentrations obtained from analytical platforms was ≤10%, which is in line with the criteria of clinical laboratory standard institute (CLSI) (CLSI, 2017). In all study centers/laboratories, the procedures for sample preparation and analysis were uniformly performed with NeoBase™ Non-derivatized MS/MS kit (PerkinElmer, MA, United States ) according to the manufacturer’s instructions. In brief, a single disc of 3.2 mm diameter was punched from each DBS and placed on a 96-well plate. An aliquot of 100 µL working solution (containing stable-isotope labeled internal standards) was added to each well. The plate was immediately covered with aluminum foil and incubated in a heating shaker at 45°C for 45 min at a speed of 750 rpm. Then, 75 µL contents from each well was transferred to a V-bottomed, heat resistant microplate for testing. The multiple reaction monitoring (MRM) mode was used in each examination, and the running parameters per instrument are listed in Supplementary Table S1. The MassLynx 4.1 and NeoLynx 4.1 software (PerkinElmer, MA, United States ) were used to analyze the raw data. The limits of detection (LoD), limits of quantification (LoQ), linear dynamic range and the total imprecision (TI) of analytes were estimated according to the protocols described in FDA 510 k documents (FDA, 2010), and the resultant data are reported in Supplementary Tables S2–S8.

### Data Collection

All participating centers/laboratories submitted a worksheet to the appointed chief of the NNSCP, which included: 1) age of subject; 2) gender of subject; 3) residence of subject; 4) ethnicity of subject (if possible); 5) health associated information of subject; 6) RIs used in routine screening practice; and 7) complete set of available amino acids and acylcarnitines test results.

### Data for RIs Calculation and the Performance Validation of RIs

The MS/MS data used in RI derivation were from subjects who passed the MS/MS NBS screening test without showing any abnormalities in the subsequent 1 year following birth. Subjects who failed the MS/MS test, or passed the MS/MS inspection but demonstrated any physical/mental retardation or suspect clinical manifestation of inborn errors of metabolism within the first year of life, were excluded from RI derivations. The performance validation of RIs was conducted in each participating center/laboratory where the level of target biomarkers in true-positives, false-positives and false-negative cases were compared to the RIs.

### Statistical Analysis

Data were analyzed in accordance with CLSI EP28-A3c guidelines (CLSI, 2010). Statistical analysis was performed using Python and R. In brief, boxplots were used to visually inspect the data; extreme outlying observations (e.g., due to a mistake in the analysis) was identified by Box-Cox transformation in conjunction with Tukey’s fences (BCT) and remove (CLSI, 2010). The distribution of each analyte was inspected by the Kolmogorov-Smirnov test. The RIs of any given analytes were partitioned arbitrarily by each day and sex or by the method of Harris and Boyd (CLSI, 2010).

The central 95% interval, corresponding to the range between the 2.5th and 97.5th percentiles, was defined as the RI. For each RI, the 90% confidence intervals were also calculated for the end points, using the bootstrapping resampling method. The nonparametric rank method was used to calculate the 1st, 2.5th, 25th, 50th, 75th, 97.5th and 99^th^ percentiles of each reference partition.

The differences between any two arbitrarily partitioned age groups of a given analyte were determined by the Kruskal-Wallis test. The resultant *p* value was adjusted to a *Q* value using the false discovery rate (FDR) algorithm (Storey, 2003), and the *Q* value <0.05 indicated statistically significant. The Mann-Whitney U test was used to examine the differences between males and females within age partitions, and the *p* value <0.05 was considered statistically significant.

## Results

**FIGURE 1:**
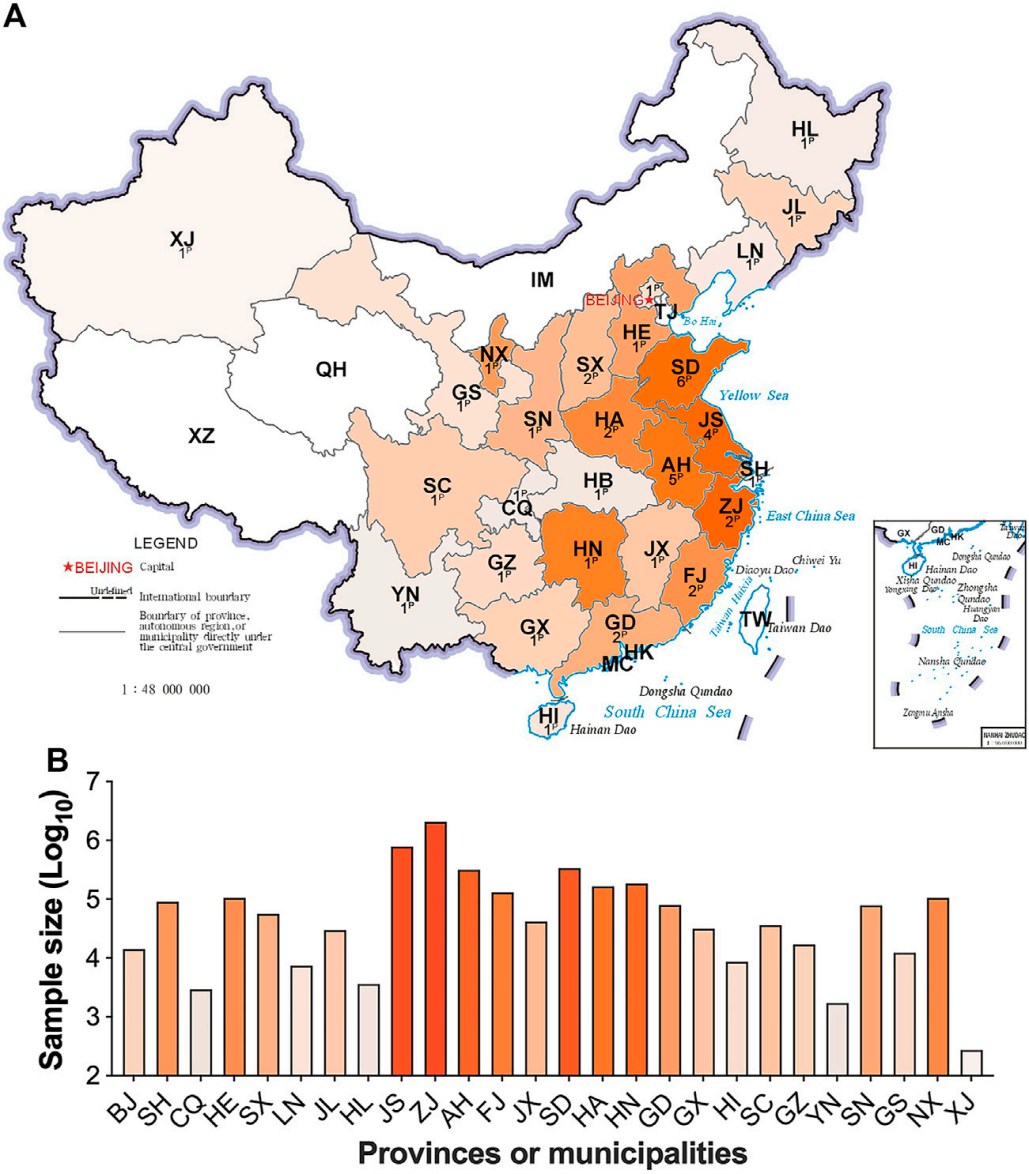
The contribution and distribution of study centers/laboratories at each province/municipality. Figure 1A, the number and location of participating centers/laboratories; Figure 1B, the sample size at each province/municipality. The superscript capital P means the number of participating centers/laboratories. HL, Heilongjiang; JL, Jilin; LN, Liaoning; XJ, Xinjiang; IM, Inner Mongolia; TJ, Tianjin, HE, Hebei; SX, Shanxi; SN, Shaanxi; NX, Ningxia; GS, Gansu; QH, Qinghai; SD, Shandong; JS, Jiangsu; AH, Anhui; HA, Henan; SH, Shanghai; HB, Hubei; CQ, Chongqing; SC, Sichuan; XZ, Tibet; ZJ, Zhejiang; JX, Jiangxi; HN, Hunan; GZ, Guizhou; YN, Yunnan; FJ, Fujian; TW, Taiwan; GD, Guangdong; GX, Guangxi; HK, Hongkong; MC, Macau. Areas with no data submitted show no color. The darker in orange, the larger in sample size.

Samples from 4,714,089 newborns who met the inclusion criteria were included in study analysis. Screening results were submitted from 43 participating centers/laboratories located in 27 provinces/municipalities of mainland China. No data were available from Tibet, Qinghai or Inner Mongolia due to the short MS/MS NBS implementation duration (Figure 1A). Most provinces/municipalities had only one or two participating centers/laboratories, whereas Shandong, Anhui and Jiangsu had four or more participating centers (Figure 1A). The number of samples examined varied among provinces/municipalities, wherein, Zhejiang screened more than two million specimens, contributing most to the dataset. In comparison, only hundreds of specimens were collected and analyzed in Xinjiang province (Figure 1B). The details of sample collection date at each province/municipality are listed in Supplementary Table S9.

**FIGURE 2:**
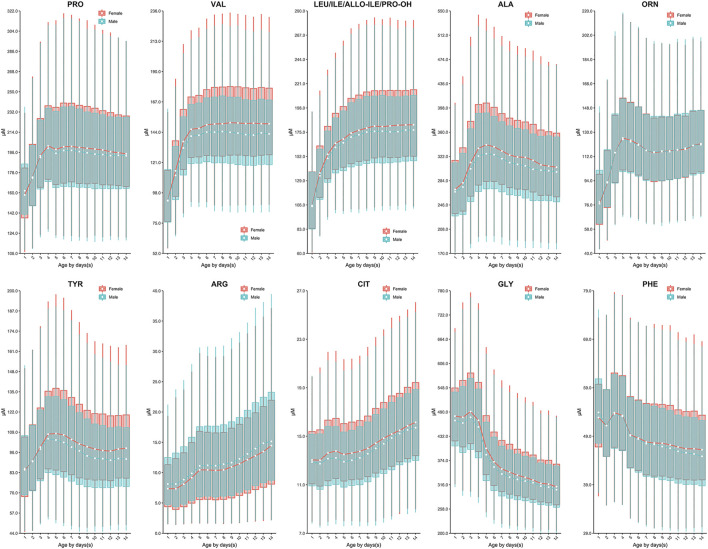
The dynamic change of amino acids except methionine over age and sex. Data of male and female partitions are shown in green and red boxes with whiskers, respectively. The boxes extend from the 25^th^ to the 75^th^ percentile, with whiskers extending to the 2.5^th^ or 97.5^th^ percentile. Medians are shown as white circles in the body of boxes, and are linked with green (male) or red (female) line to shown the dynamics. Abbreviations are listed in the legend of Table 2.

Continuous change in reference end points (2.5th and 97.5th) over age and/or sex for each analyte are shown in Table 2. Additional percentiles (1st, 25th, 50th, 75th and 99th) are presented in Supplementary Table S10. The RIs determined by Harris and Boyd approach are presented in Supplementary Table S11. Both age and sex were found to be important factors affecting the value of testing biomarkers (Table 1). Females demonstrated a higher 2.5th and 97.5th percentile value than males in all test amino acids except arginine and ornithine, irrespective of age (Table 2). Proline, valine and leucine/isoleucine/alloisoleucine/hydroxyproline demonstrated a similar value distribution with increasing concentrations throughout the first week of life, followed by constant levels from 7 to 14 days (Figure 2). Increased levels of alanine, ornithine and tyrosine were also observed throughout the first week of life, but demonstrated a more dynamic pattern from 7 to 14 days, decreasing slightly (Figure 2). Despite most amino acids fluctuating during the first week of life, citrulline and arginine showed a consistent increase from birth to 14 days of life (Figure 2). In contrast, glycine and phenylalanine presented a consistent decrease across the screening age range (Figure 2).

**TABLE 2:** The Reference intervals partitioned by each day and sex for 35 MS/MS NBS biomarkers (μM).

Analyte	Age	Amino acids					
		Male			Female		
		Lower limit (2.5^th^) and 90% CI	Upper limit (97.5^th^) and 90% CI	No. of samples	Lower limit (2.5^th^) and 90% CI	Upper limit (97.5^th^) and 90% CI	No. of samples
ALA	0 to ≤1 day (***a***)	**171.0 (168.7–172.8)**	**404.2 (402.1–406.4)**	**10,291**	**171.8 (169.5–173.9)**	**406.7 (404.3–409.2)**	**9,540**
	1 day to ≤2 days (***b***)	**163.6 (163.0–166.3)**	**435.9 (435.2–436.8)**	**115,292**	**168.5 (167.8–169.0)**	**448.4 (447.3–449.4)**	**105,039**
	2 days to ≤3 days (***c***)	**178.0 (177.8–181.3)**	**495.8 (495.4–496.1)**	**1,053,952**	**185.0 (184.8–185.2)**	**510.5 (510.1–510.9)**	**965,270**
	3 days to ≤4 days (***d***)	**188.2 (188.0–191.5)**	**527.4 (527.0–527.8)**	**801,271**	**195.9 (195.6–196.2)**	**545.3 (544.8–545.8)**	**729,980**
	4 days to ≤5 days (*e*)	**188.1 (187.6–192.7)**	**519.6 (518.6–520.4)**	**158,961**	**197.3 (196.6–197.9)**	**539.1 (538.1–540.3)**	**143,970**
	5 days to ≤6 days (***f***)	**188.0 (187.1–192.8)**	**508.0 (506.7–509.1)**	**96,903**	**197.1 (196.3–198.0)**	**526.2 (525.0–527.4)**	**87,591**
	6 days to ≤7 days (***g***)	**184.1 (183.2–189.9)**	**496.2 (494.9–497.5)**	**59,713**	**195.2 (194.1–196.1)**	**511.5 (510.2–513.3)**	**53,406**
	7 days to ≤8 days (***h***)	**182.3 (180.8–186.7)**	**488.0 (485.8–489.8)**	**33,973**	**190.4 (189.1–192.0)**	**503.6 (501.7–505.8)**	**29,789**
	8 days to ≤9 days (***i***)	**180.0 (179.0–185.2)**	**482.1 (479.7–484.3)**	**25,407**	**190.2 (188.3–191.8)**	**495.9 (492.9–497.9)**	**22,233**
	9 days to ≤10 days (***j***)	**178.3 (177.0–183.4)**	**479.5 (477.4–481.9)**	**19,866**	**189.1 (187.7–190.8)**	**492.5 (489.7–494.8)**	**17,364**
	10 days to ≤11 days (***k***)	**178.5 (176.5–184.1)**	**473.0 (470.2–475.9)**	**15,363**	**189.6 (187.9–191.2)**	**488.7 (486.6–491.9)**	**13,365**
	11 days to ≤12 days (***L***)	**178.4 (176.5–182.9)**	**466.9 (464.5–470.1)**	**14,117**	**187.0 (184.9–188.4)**	**479.8 (477.1–481.9)**	**12,293**
	12 days to ≤13 days (***lm***)	**176.6 (174.8–182.2)**	**464.9 (462.5–467.9)**	**12,489**	**186.3 (184.4–188.4)**	**471.0 (468.0–473.3)**	**11,147**
	13 days to ≤14 days (***m***)	**176.4 (174.6–182.0)**	**466.3 (464.7–468.7)**	**11,465**	**186.1 (184.0–188.0)**	**467.5 (465.1–471.1)**	**10,117**
ARG	0 to ≤1 day (***a***)	**1.5 (1.5–1.6)**	**21.3 (21.0–21.5)**	**10,355**	**1.5 (1.4–1.5)**	**19.4 (19.2–19.7)**	**9,613**
	1 day to ≤2 days (***a***)	**1.4 (1.4–1.4)**	**23.8 (23.7–23.9)**	**116,704**	**1.4 (1.4–1.4)**	**22.5 (22.4–22.6)**	**105,982**
	2 days to ≤3 days (***b***)	**1.5 (1.5–1.5)**	**25.4 (25.3–25.4)**	**1,062,374**	**1.5 (1.5–1.5)**	**23.8 (23.8–23.8)**	**971,780**
	3 days to ≤4 days (***c***)	**1.7 (1.7–1.7)**	**28.0 (28.0–28.0)**	**806,759**	**1.6 (1.6–1.6)**	**26.8 (26.7–26.8)**	**734,811**
	4 days to ≤5 days (***def***)	**1.7 (1.7–1.7)**	**30.7 (30.7–30.8)**	**159,837**	**1.6 (1.6–1.6)**	**29.8 (29.7–29.9)**	**144,722**
	5 days to ≤6 days (***d***)	**1.7 (1.6–1.7)**	**30.9 (30.8–31.0)**	**97,358**	**1.6 (1.6–1.6)**	**29.3 (29.2–29.5)**	**87,942**
	6 days to ≤7 days (***e***)	**1.6 (1.6–1.6)**	**30.9 (30.7–31.0)**	**60,025**	**1.6 (1.6–1.6)**	**29.2 (29.0–29.3)**	**53,625**
	7 days to ≤8 days (***f***)	**1.6 (1.6–1.6)**	**31.1 (30.8–31.2)**	**34,169**	**1.5 (1.5–1.6)**	**29.3 (29.1–29.6)**	**29,956**
	8 days to ≤9 days (***g***)	**1.6 (1.6–1.6)**	**32.3 (32.1–32.5)**	**25,571**	**1.6 (1.6–1.6)**	**30.5 (30.3–30.8)**	**22,326**
	9 days to ≤10 days (***h***)	**1.7 (1.7–1.8)**	**33.3 (33.1–33.6)**	**19,992**	**1.7 (1.6–1.7)**	**31.6 (31.3–31.8)**	**17,453**
	10 days to ≤11 days (***i***)	**1.9 (1.8–1.9)**	**34.9 (34.5–35.2)**	**15,431**	**1.9 (1.8–1.9)**	**33.1 (32.8–33.4)**	**13,398**
	11 days to ≤12 days (***j***)	**2.1 (2.0–2.1)**	**37.3 (37.0–37.7)**	**14,169**	**1.9 (1.9–2.0)**	**34.3 (34.0–34.6)**	**12,333**
	12 days to ≤13 days (***k***)	**2.2 (2.2–2.3)**	**38.3 (37.8–38.6)**	**12,545**	**2.0 (2.0–2.1)**	**35.8 (35.5–36.2)**	**11,181**
	13 days to ≤14Vdays (***L***)	**2.1 (2.1–2.2)**	**39.6 (39.2–40.0)**	**11,513**	**2.2 (2.1–2.3)**	**37.3 (36.9–37.6)**	**10,146**
CIT	0 to ≤1 day (***a***)	**7.8 (7.7–7.9)**	**20.0 (19.9–20.2)**	**10,250**	**7.9 (7.9–8.0)**	**20.0 (19.8–20.1)**	**9,535**
	1 day to ≤2 days (***a***)	**7.5 (7.4–7.5)**	**20.3 (20.2–20.3)**	**115,213**	**7.6 (7.6–7.7)**	**20.7 (20.7–20.8)**	**104,802**
	2 days to ≤3 days (***b***)	**7.6 (7.6–7.6)**	**21.3 (21.3–21.3)**	**1,048,453**	**7.9 (7.9–8.0)**	**22.0 (22.0–22.0)**	**959,730**
	3 days to ≤4 days (***c***)	**7.8 (7.7–7.8)**	**21.2 (21.2–21.2)**	**797,190**	**8.1 (8.1–8.1)**	**22.1 (22.1–22.1)**	**726,262**
	4 days to ≤5 days (***d***)	**7.6 (7.6–7.6)**	**20.5 (20.4–20.5)**	**157,824**	**8.0 (8.0–8.0)**	**21.4 (21.4–21.4)**	**143,092**
	5 days to ≤6 days (***e***)	**7.7 (7.6–7.7)**	**20.6 (20.5–20.6)**	**96,245**	**8.0 (8.0–8.0)**	**21.4 (21.4–21.5)**	**86,916**
	6 days to ≤7 days (***b***)	**7.6 (7.5–7.6)**	**21.0 (21.0–21.1)**	**59,179**	**8.0 (7.9–8.0)**	**21.7 (21.6–21.8)**	**52,850**
	7 days to ≤8 days (***f***)	**7.6 (7.5–7.7)**	**21.7 (21.6–21.7)**	**33,642**	**7.9 (7.8–7.9)**	**22.4 (22.3–22.5)**	**29,458**
	8 days to ≤9 days (***g***)	**7.6 (7.5–7.7)**	**22.5 (22.4–22.6)**	**25,126**	**8.0 (8.0–8.1)**	**23.2 (23.1–23.3)**	**21,940**
	9 days to ≤10 days (***h***)	**7.9 (7.8–9.0)**	**23.3 (23.2–23.5)**	**19,643**	**8.3 (8.2–8.3)**	**23.9 (23.7–24.0)**	**17,187**
	10 days to ≤11 days (***i***)	**8.1 (8.0–8.2)**	**24.0 (23.8–24.1)**	**15,175**	**8.4 (8.3–8.5)**	**24.8 (24.7–25.0)**	**13,189**
	11 days to ≤12 days (***j***)	**8.7 (8.6–8.9)**	**24.5 (24.3–24.7)**	**13,972**	**8.6 (8.4–8.7)**	**25.0 (24.9–25.2)**	**12,105**
	12 days to ≤13 days (***k***)	**8.6 (8.4–8.7)**	**24.9 (24.8–25.1)**	**12,342**	**9.0 (8.9–9.1)**	**25.5 (25.3–25.8)**	**10,995**
	13 days to ≤14 days (***L***)	**8.7 (8.6–8.8)**	**25.3 (25.1–25.5)**	**11,379**	**9.0 (8.9–9.1)**	**26.1 (26.0–26.3)**	**9,951**
GLY	0 to ≤1 day (***a***)	**310.4 (306.9–316.7)**	**682.4 (679.4–684.9)**	**10,321**	**317.1 (313.1–320.2)**	**691.6 (688.3–694.8)**	**9,587**
	1 day to ≤2 days (***b***)	**289.6 (288.5–295.6)**	**738.1 (736.7–739.4)**	**115,855**	**301.7 (300.8–302.8)**	**753.6 (751.8–755.2)**	**105,328**
	2 days to ≤3 days (***c***)	**287.1 (286.8–292.4)**	**766.7 (766.2–767.2)**	**1,052,604**	**299.4 (299.0–299.7)**	**777.6 (777.0–778.2)**	**963,360**
	3 days to ≤4 days (***d***)	**273.0 (272.7–279.4)**	**737.1 (736.5–737.6)**	**799,784**	**286.3 (285.9–286.6)**	**751.5 (750.9–752.0)**	**729,189**
	4 days to ≤5 days (***e***)	**238.1 (237.4–243.9)**	**630.8 (629.7–631.8)**	**158,084**	**250.0 (249.3–250.7)**	**646.3 (645.2–647.6)**	**143,631**
	5 days to ≤6 days (***f***)	**222.9 (222.1–227.8)**	**580.3 (579.0–581.4)**	**95,506**	**232.2 (231.3–233.0)**	**592.8 (591.4–594.6)**	**86,842**
	6 days to ≤7 days (***g***)	**213.4 (212.4–217.3)**	**550.0 (548.5–551.8)**	**58,328**	**222.5 (221.5–223.6)**	**566.8 (565.5–568.6)**	**52,592**
	7 days to ≤8 days (***h***)	**211.0 (210.0–214.0)**	**538.8 (537.0–541.0)**	**33,096**	**218.4 (217.3–219.8)**	**554.7 (552.7–557.3)**	**29,265**
	8 days to ≤9 days (***i***)	**207.5 (205.5–212.1)**	**524.5 (522.4–526.7)**	**24,687**	**216.7 (215.3–218.4)**	**536.4 (534.2–539.1)**	**21,780**
	9 days to ≤10 days (***j***)	**207.8 (206.3–211.5)**	**513.4 (511.6–516.3)**	**19,284**	**213.7 (211.9–215.4)**	**526.2 (523.7–528.0)**	**16,971**
	10 days to ≤11 days (***k***)	**204.1 (202.3–208.1)**	**506.5 (503.9–509.2)**	**14,784**	**212.6 (210.6–214.1)**	**515.2 (513.2–518.9)**	**13,016**
	11 days to ≤12 days (***L***)	**201.0 (199.1–204.2)**	**487.7 (483.8–490.6)**	**13,520**	**206.3 (204.0–208.1)**	**494.8 (492.4–497.3)**	**11,882**
	12 days to ≤13 days (***L***)	**201.9 (200.4–205.8)**	**484.2 (481.6–486.2)**	**11,973**	**207.9 (206.2–210.7)**	**494.4 (491.2–497.8)**	**10,764**
	13 days to ≤14 days (***m***)	**198.7 (197.4–201.8)**	**479.3 (477.0–481.5)**	**10,879**	**204.7 (203.2–206.5)**	**482.0 (478.9–484.9)**	**9,688**
LEU/ILE/ALLO-ILE/PRO-OH	0 to ≤1 day (***a***)	61.2 (60.8–61.9)	195.0 (193.5–196.1)	16,934	61.2 (60.8–61.9)	195.0 (193.5–196.1)	16,934
	1 day to ≤2 days (***b***)	**76.0 (75.6–76.2)**	**210.5 (210.1–210.9)**	**113,894**	**76.8 (76.5–77.1)**	**214.8 (214.3–215.3)**	**103,619**
	2 days to ≤3 days (***c***)	**92.0 (91.9–93.1)**	**229.4 (229.3–229.6)**	**1,055,947**	**94.5 (94.4–94.6)**	**234.7 (234.5–234.9)**	**966,309**
	3 days to ≤4 days (***d***)	**98.2 (98.1–99.4)**	**245.7 (245.5–245.9)**	**801,425**	**101.3 (101.2–101.4)**	**250.9 (250.7–251.1)**	**729,610**
	4 days to ≤5 days (***e***)	**97.6 (97.3–98.9)**	**257.6 (257.2–258.1)**	**158,196**	**100.2 (99.8–100.5)**	**262.2 (261.7–262.7)**	**143,037**
	5 days to ≤6 days (***f***)	**99.0 (98.6–100.4)**	**268.8 (268.2–269.4)**	**96,026**	**101.9 (101.5–102.2)**	**274.0 (273.5–274.7)**	**86,576**
	6 days to ≤7 days (***g***)	**98.3 (97.8–100.7)**	**273.5 (272.6–274.3)**	**59,038**	**101.7 (101.1–102.4)**	**278.2 (277.5–279.1)**	**52,609**
	7 days to ≤8 days (***h***)	**100.2 (99.4–102.0)**	**277.6 (276.7–278.6)**	**33,575**	**102.5 (101.6–103.3)**	**282.0 (281.0–283.3)**	**29,328**
	8 days to ≤9 days (***i***)	**100.4 (99.7–102.2)**	**277.5 (276.2–278.6)**	**25,098**	**103.6 (103.0–104.2)**	**284.4 (282.8–285.9)**	**21,809**
	9days to ≤10 days (***ij***)	**100.4 (99.7–103.2)**	**277.3 (276.1–278.4)**	**19,623**	**104.9 (104.0–105.9)**	**282.7 (281.4–284.2)**	**17,052**
	10 days to ≤11 days (***ij***)	**101.3 (100.5–103.5)**	**276.6 (274.9–278.0)**	**15,166**	**105.1 (103.7–105.9)**	**284.2 (282.8–285.7)**	**13,113**
	11 days to ≤12 days (***ik***)	**101.5 (100.4–103.8)**	**273.6 (272.0–274.6)**	**13,970**	**105.2 (104.0–106.8)**	**282.0 (280.3–283.1)**	**12,100**
	12 days to ≤13 days (***jk***)	**100.3 (99.3–103.3)**	**275.5 (274.0–277.3)**	**12,372**	**106.3 (105.2–107.4)**	**281.2 (279.4–282.2)**	**10,959**
	13 days to ≤14 days (***k***)	**102.5 (100.9–105.8)**	**274.2 (272.2–275.7)**	**11,362**	**104.6 (102.7–105.5)**	**281.8 (279.9–283.0)**	**9,966**
MET	0 to ≤1 day (***a***)	11.7 (11.6–11.8)	31.0 (30.8–31.1)	19,894	11.7 (11.6–11.8)	31.0 (30.8–31.1)	19,894
	1 day to ≤2 days (***b***)	**10.5 (10.4–10.5)**	**31.0 (31.0–31.1)**	**116,118**	**10.8 (10.7–10.9)**	**32.4 (32.3–32.5)**	**105,323**
	2 days to ≤3 days (***c***)	**9.0 (9.0–9.0)**	**30.5 (30.5–30.5)**	**1,054,481**	**9.5 (9.5–9.5)**	**31.8 (31.8–31.8)**	**965,052**
	3 days to ≤4 days (***d***)	**9.0 (9.0–9.0)**	**31.3 (31.3–31.3)**	**800,415**	**9.6 (9.6–9.6)**	**32.7 (32.7–32.8)**	**729,270**
	4 days to ≤5 days (***e***)	**8.7 (8.7–8.8)**	**30.5 (30.4–30.6)**	**158,368**	**9.2 (9.1–9.2)**	**31.8 (31.7–31.9)**	**143,444**
	5 days to ≤6 days (***f***)	**8.5 (8.5–8.6)**	**29.9 (29.9–30.0)**	**96,333**	**9.0 (9.0–9.0)**	**31.1 (31.0–31.1)**	**87,247**
	6 days to ≤7 days (***g***)	**8.7 (8.6–8.7)**	**30.0 (29.9–30.1)**	**59,410**	**9.0 (8.9–9.0)**	**30.8 (30.7–30.9)**	**53,126**
	7 days to ≤8 days (***d***)	**8.9 (8.9–9.0)**	**30.7 (30.6–30.9)**	**33,839**	**9.2 (9.1–9.3)**	**31.3 (31.2–31.5)**	**29,756**
	8 days to ≤9 days (***h***)	**9.0 (8.9–9.1)**	**31.4 (31.3–31.6)**	**25,370**	**9.6 (9.5–9.7)**	**32.2 (32.0–32.4)**	**22,141**
	9 days to ≤10 days (***i***)	**9.3 (9.2–9.5)**	**32.4 (32.2–32.5)**	**19,833**	**9.7 (9.5–9.9)**	**33.0 (32.9–33.2)**	**17,301**
	10 days to ≤11 days (***j***)	**9.5 (9.4–9.7)**	**33.3 (33.1–33.6)**	**15,292**	**9.8 (9.7–10.0)**	**33.7 (33.4–33.9)**	**13,286**
	11 days to ≤12 days (***a***)	**9.9 (9.9–10.1)**	**33.5 (33.2–33.7)**	**14,085**	**9.9 (9.8–10.1)**	**34.2 (34.0–34.5)**	**12,208**
	12 days to ≤13 days (***k***)	**10.0 (9.8–10.1)**	**34.2 (34.0–34.4)**	**12,442**	**10.2 (10.0–10.4)**	**34.8 (34.5–35.1)**	**11,069**
	13 days to ≤14 days (***L***)	**10.0 (9.9–10.2)**	**34.9 (34.6–35.1)**	**11,418**	**10.5 (10.3–10.6)**	**35.2 (34.9–35.4)**	**10,050**
ORN	0 to ≤1 day (***a***)	**42.9 (42.4–43.2)**	**149.6 (147.9–150.4)**	**9,480**	**43.3 (42.9–43.9)**	**145.0 (143.8–145.8)**	**8,768**
	1 day to ≤2 days (***b***)	**50.0 (49.9–49.3)**	**169.7 (169.3–170.2)**	**112,878**	**49.6 (49.4–49.7)**	**169.0 (168.7–169.4)**	**102,396**
	2 days to ≤3 days (***c***)	**61.1 (61.0–61.1)**	**205.3 (205.2–205.5)**	**1,049,193**	**61.7 (61.7–61.8)**	**202.0 (201.9–202.2)**	**961,443**
	3 days to ≤4 days (***d***)	**67.2 (67.1–67.3)**	**219.4 (219.2–219.5)**	**798,075**	**68.5 (68.4–68.6)**	**218.1 (217.9–218.3)**	**728,161**
	4 days to ≤5 days (***e***)	**66.2 (65.9–66.4)**	**212.1 (211.7–212.5)**	**158,479**	**67.3 (67.0–67.5)**	**212.3 (211.8–212.6)**	**143,683**
	5 days to ≤6 days (***f***)	**64.2 (63.9–65.5)**	**208.7 (208.3–209.3)**	**96,493**	**65.6 (65.3–65.9)**	**207.5 (207.0–208.1)**	**87,300**
	6 days to ≤7 days (***g***)	**61.8 (61.5–62.3)**	**200.6 (200.0–201.1)**	**59,402**	**63.0 (62.6–63.4)**	**200.4 (199.8–201.1)**	**53,184**
	7 days to ≤8 days (***h***)	**61.4 (60.8–62.9)**	**198.0 (197.2–199.0)**	**33,810**	**61.9 (61.4–62.5)**	**196.2 (195.5–197.0)**	**29,639**
	8 days to ≤9 days (***chi***)	61.8 (61.2–62.2)	196.7 (196.2–197.4)	47,369	61.8 (61.2–62.2)	196.7 (196.2–197.4)	47,369
	9 days to ≤10 days (***i***)	63.0 (62.4–63.3)	195.8 (195.2–196.5)	37,062	63.0 (62.4–63.3)	195.8 (195.2–196.5)	37,062
	10 days to ≤11 days (***gk***)	63.8 (63.4–64.4)	198.6 (197.6–199.5)	28,618	63.8 (63.4–64.4)	198.6 (197.6–199.5)	28,618
	11 days to ≤12 days (***k***)	**64.1 (63.3–64.9)**	**199.4 (198.4–200.2)**	**14,093**	**64.1 (63.1–64.9)**	**195.2 (194.0–196.3)**	**12,229**
	12 days to ≤13 days (***f***)	66.5 (65.9–67.2)	200.4 (199.5–201.8)	23,587	66.5 (65.9–67.2)	200.4 (199.5–201.8)	23,587
	13 days to ≤14 days (***f***)	67.5 (67.0–68.0)	200.0 (199.0–200.8)	21,545	67.5 (67.0–68.0)	200.0 (199.0–200.8)	21,545
PEH	0 to ≤1 day (***a***)	**37.3 (37.0–37.6)**	**75.3 (74.8–75.7)**	**10,289**	**36.6 (36.3–37.0)**	**73.6 (73.2–74.0)**	**9,566**
	1 day to ≤2 days (***b***)	34.7 (34.7–34.8)	74.2 (74.1–74.3)	221,146	34.7 (34.7–34.8)	74.2 (74.1–74.3)	221,146
	2 days to ≤3 days (***c***)	**35.4 (35.3–35.4)**	**78.5 (78.4–78.5)**	**1,053,903**	**35.5 (35.4–35.5)**	**78.9 (78.8–78.9)**	**963,641**
	3 days to ≤4 days (***a***)	**34.8 (34.8–34.8)**	**78.0 (78.0–78.1)**	**799,887**	**34.9 (34.9–35.0)**	**78.2 (78.2–78.3)**	**728,624**
	4 days to ≤5 days (***d***)	**32.0 (31.9–32.1)**	**73.2 (73.1–73.4)**	**157,689**	**32.1 (32.0–32.2)**	**73.6 (73.5–73.7)**	**142,792**
	5 days to ≤6 days (***e***)	**31.1 (31.0–31.2)**	**72.7 (72.6–72.9)**	**95,700**	**31.4 (31.3–31.5)**	**72.6 (72.5–72.8)**	**86,626**
	6 days to ≤7 days (***f***)	**30.4 (30.2–30.5)**	**71.9 (71.8–72.2)**	**58,792**	**30.7 (30.6–30.9)**	**71.9 (71.7–72.1)**	**52,598**
	7 days to ≤8 days (***g***)	**30.0 (29.8–30.1)**	**71.5 (71.2–71.8)**	**33,366**	**30.1 (29.9–30.3)**	**72.3 (72.1–72.6)**	**29,279**
	8 days to ≤9 days (***h***)	**29.6 (29.5–30.9)**	**71.1 (70.9–71.3)**	**24,907**	**30.3 (29.9–30.5)**	**72.1 (71.8–72.3)**	**21,833**
	9 days to ≤10 days (***i***)	**29.6 (29.3–30.8)**	**70.9 (70.6–71.2)**	**19,481**	**30.1 (29.8–30.3)**	**72.0 (71.8–72.4)**	**17,056**
	10 days to ≤11 days (***j***)	**29.4 (29.1–29.7)**	**69.1 (68.7–69.4)**	**15,017**	**29.8 (29.5–30.1)**	**70.6 (70.3–70.9)**	**13,080**
	11 days to ≤12 days (***k***)	**29.3 (29.1–29.5)**	**68.6 (68.4–68.9)**	**13,807**	**30.0 (29.8–30.4)**	**69.5 (69.1–69.8)**	**12,052**
	12 days to ≤13 days (***L***)	**29.3 (29.0–29.6)**	**68.1 (67.8–68.5)**	**12,221**	**30.1 (29.8–30.4)**	**70.1 (69.7–70.5)**	**10,939**
	13 days to ≤14 days (***m***)	**29.2 (28.7–29.4)**	**67.7 (67.3–68.0)**	**11,218**	**30.3 (29.9–30.5)**	**68.7 (68.3–69.1)**	**9,947**
PRO	0 to ≤1 day (***a***)	**109.2 (108.3–108.2)**	**237.0 (235.6–238.7)**	**10,251**	**107.4 (106.6–108.4)**	**231.7 (230.3–233.2)**	**9,471**
	1 day to ≤2 days (***b***)	**109.8 (109.5–110.2)**	**262.6 (262.1–263.2)**	**115,138**	**110.6 (110.3–111.0)**	**264.1 (263.6–264.6)**	**104,636**
	2 days to ≤3 days (***c***)	**120.3 (120.2–121.5)**	**294.2 (294.0–294.4)**	**1,052,842**	**122.3 (122.2–122.4)**	**295.2 (295.0–295.4)**	**963,922**
	3 days to ≤4 days (***d***)	**125.3 (125.1–126.4)**	**310.4 (310.1–310.6)**	**797,965**	**127.6 (127.4–127.8)**	**312.6 (312.3–312.8)**	**727,183**
	4 days to ≤5 days (***e***)	**119.7 (119.4–121.1)**	**310.7 (310.1–311.2)**	**157,703**	**122.1 (121.8–122.3)**	**314.3 (313.7–314.8)**	**143,055**
	5 days to ≤6 days (***f***)	**118.5 (117.9–120.9)**	**316.5 (315.8–317.1)**	**96,029**	**121.4 (120.9–121.9)**	**320.6 (319.8–321.4)**	**86,822**
	6 days to ≤7 days (***f***)	**117.0 (116.3–119.5)**	**316.1 (315.2–317.2)**	**59,148**	**121.7 (121.1–122.3)**	**319.1 (318.2–319.9)**	**52,980**
	7 days to ≤8 days (***g***)	**117.4 (116.7–119.1)**	**313.8 (312.5–315.0)**	**33,731**	**120.7 (119.9–121.3)**	**316.0 (314.9–317.5)**	**29,599**
	8 days to ≤9 days (***eh***)	**116.8 (116.1–118.7)**	**306.8 (305.1–308.3)**	**25,231**	**120.0 (119.1–121.1)**	**314.4 (313.2–315.9)**	**22,058**
	9 days to ≤10 days (***hi***)	**116.2 (114.8–119.0)**	**306.2 (304.9–307.4)**	**19,744**	**121.8 (120.9–122.9)**	**310.3 (308.7–312.1)**	**17,283**
	10 days to ≤11 days (***ij***)	**116.7 (115.8–119.9)**	**302.1 (300.1–303.5)**	**15,237**	**120.5 (118.8–121.4)**	**305.7 (304.1–307.3)**	**13,279**
	11 days to ≤12 days (***jk***)	**117.9 (116.8–119.4)**	**300.2 (298.5–301.7)**	**14,052**	**121.3 (120.2–122.7)**	**303.4 (301.7–305.1)**	**12,240**
	12 days to ≤13 days (***k***)	**117.8 (116.8–119.8)**	**299.1 (297.2–300.8)**	**12,432**	**120.6 (119.8–121.6)**	**298.6 (297.3–300.1)**	**11,100**
	13 days to ≤14 days (***k***)	**117.6 (116.2–119.3)**	**295.9 (294.3–297.6)**	**11,413**	**120.0 (118.6–121.8)**	**295.7 (293.0–297.8)**	**10,081**
TYR	0 to ≤1 day (***a***)	**45.5 (45.0–45.1)**	**152.5 (151.4–153.7)**	**10,244**	**45.0 (44.4–45.8)**	**150.6 (149.2–152.0)**	**9,485**
	1 day to ≤2 days (***b***)	45.6 (45.4–45.7)	162.4 (162.1–162.7)	219,700	45.6 (45.4–45.7)	162.4 (162.1–162.7)	219,700
	2 days to ≤3 days (***c***)	**49.7 (49.6–50.7)**	**176.6 (176.4–176.7)**	**1,050,528**	**50.7 (50.6–50.8)**	**177.6 (177.5–177.7)**	**962,728**
	3 days to ≤4 days (***d***)	**52.9 (52.8–53.0)**	**189.1 (188.9–189.2)**	**797,519**	**54.5 (54.4–54.6)**	**193.5 (193.3–193.7)**	**727,587**
	4 days to ≤5 days (***e***)	**50.4 (50.2–51.7)**	**190.4 (189.9–190.8)**	**157,698**	**52.4 (52.2–52.7)**	**198.2 (197.8–198.6)**	**142,924**
	5 days to ≤6 days (***f***)	**48.6 (48.3–50.0)**	**188.1 (187.6–188.5)**	**95,962**	**50.6 (50.2–50.8)**	**196.0 (195.4–196.5)**	**86,967**
	6 days to ≤7 days (***g***)	**46.5 (46.1–47.9)**	**181.5 (181.0–182.1)**	**59,135**	**48.8 (48.5–49.3)**	**188.0 (187.4–188.6)**	**52,983**
	7 days to ≤8 days (***h***)	**45.0 (44.5–46.4)**	**172.3 (171.5–172.9)**	**33,698**	**47.8 (47.4–48.6)**	**180.2 (179.3–181.2)**	**29,630**
	8 days to ≤9 days (***i***)	**44.8 (44.4–46.2)**	**166.7 (166.2–167.6)**	**25,164**	**47.6 (47.1–48.1)**	**173.2 (172.7–174.2)**	**22,130**
	9 days to ≤10 days (***j***)	**45.0 (44.4–46.6)**	**159.6 (158.4–160.5)**	**19,724**	**48.4 (47.9–49.1)**	**167.9 (167.0–168.7)**	**17,298**
	10 days to ≤11 days (***jk***)	**45.0 (44.2–46.6)**	**157.4 (156.0–158.6)**	**15,256**	**48.2 (47.3–49.1)**	**166.5 (165.2–167.7)**	**13,301**
	11 days to ≤12 days (***k***)	**46.1 (45.7–47.9)**	**154.9 (154.1–156.0)**	**14,046**	**49.0 (47.9–49.9)**	**164.9 (163.9–166.6)**	**12,242**
	12 days to ≤13 days (***j***)	**45.9 (45.2–47.9)**	**152.8 (151.5–153.8)**	**12,420**	**49.6 (49.0–50.6)**	**164.0 (162.9–165.1)**	**11,112**
VAL	13 days to ≤14 days (***j***)	**45.8 (45.2–47.5)**	**152.5 (151.3–153.8)**	**11,409**	**49.3 (48.5–50.3)**	**165.5 (164.3–167.2)**	**10,070**
	0 to ≤1 day (***a***)	56.2 (55.9–56.5)	161.1 (160.2–161.8)	17,402	56.2 (55.9–56.5)	161.1 (160.2–161.8)	17,402
	1 day to ≤2 days (***b***)	**64.9 (64.7–65.2)**	**179.1 (178.7–179.5)**	**112,450**	**66.4 (66.1–66.6)**	**184.9 (184.4–185.3)**	**102,614**
	2 days to ≤3 days (***c***)	**79.5 (79.4–80.6)**	**204.4 (204.3–204.6)**	**1,051,646**	**82.4 (82.3–82.5)**	**211.2 (211.1–211.4)**	**962,851**
	3 days to ≤4 days (***d***)	**87.4 (87.2–89.5)**	**214.9 (214.8–215.1)**	**800,721**	**91.1 (90.9–91.2)**	**221.7 (221.5–221.8)**	**728,786**
	4 days to ≤5 days (***e***)	**87.3 (87.0–88.5)**	**216.6 (216.2–216.9)**	**158,645**	**90.6 (90.3–90.9)**	**223.7 (223.4–224.2)**	**143,548**
	5 days to ≤6 days (***f***)	**86.9 (86.6–88.2)**	**222.8 (222.3–223.3)**	**96,537**	**90.8 (90.6–91.2)**	**230.6 (230.0–231.1)**	**87,120**
	6 days to ≤7 days (***f***)	**84.1 (83.6–86.4)**	**225.3 (224.7–225.8)**	**59,428**	**88.4 (88.0–88.9)**	**233.7 (232.8–234.5)**	**53,058**
	7 days to ≤8 days (***f***)	**84.0 (83.2–86.6)**	**226.5 (225.8–227.2)**	**33,775**	**87.7 (87.1–88.2)**	**234.4 (233.8–235.1)**	**29,574**
	8 days to ≤9 days (***f***)	**83.3 (82.5–85.0)**	**224.8 (224.2–225.9)**	**25,296**	**88.3 (87.4–88.8)**	**235.2 (234.2–236.4)**	**22,039**
	9 days to ≤10 days (***f***)	**83.0 (82.3–86.9)**	**225.5 (223.9–226.5)**	**19,771**	**88.4 (87.3–89.4)**	**234.2 (233.4–235.2)**	**17,232**
	10 days to ≤11 days (***g***)	**83.5 (82.7–85.3)**	**222.1 (220.8–222.9)**	**15,300**	**88.5 (87.5–89.4)**	**233.3 (231.8–234.6)**	**13,266**
	11 days to ≤12 days (***g***)	**83.1 (82.1–86.1)**	**220.9 (219.7–222.7)**	**14,075**	**88.5 (87.3–89.6)**	**232.0 (230.7–233.0)**	**12,206**
	12 days to ≤13 days (***fg***)	**83.1 (82.4–86.4)**	**222.5 (220.9–223.9)**	**12,453**	**88.8 (87.8–90.0)**	**234.1 (232.3–235.8)**	**11,089**
	13 days to ≤14 days (***g***)	**83.5 (82.7–85.3)**	**220.5 (219.0–222.2)**	**11,422**	**88.9 (87.7–90.2)**	**231.8 (230.8–233.5)**	**10,063**
**Acylcarnitines**							
C0	0 to ≤1 day (***a***)	**10.44 (10.30–10.61)**	**33.08 (32.85–33.38)**	**10,290**	**10.14 (10.03–10.29)**	**29.97 (29.73–30.26)**	**9,518**
	1 day to ≤2 days (***b***)	**10.30 (10.27–10.35)**	**34.70 (34.62–34.78)**	**115,510**	**9.75 (9.71–9.80)**	**31.88 (31.80–31.97)**	**104,661**
	2 days to ≤3 days (***c***)	**11.30 (11.28–10.31)**	**37.73 (37.70–37.76)**	**1,053,208**	**10.65 (10.64–10.66)**	**34.69 (34.66–34.71)**	**964,639**
	3 days to ≤4 days (***d***)	**11.88 (11.86–11.90)**	**38.59 (38.55–38.62)**	**800,275**	**11.09 (11.07–11.11)**	**35.64 (35.60–35.67)**	**730,370**
	4 days to ≤5 days (***d***)	**11.86 (11.81–11.90)**	**38.23 (38.15–38.30)**	**158,847**	**11.05 (11.01–11.09)**	**35.71 (35.64–35.77)**	**143,909**
	5 days to ≤6 days (***e***)	**11.84 (11.78–11.90)**	**38.62 (38.53–38.68)**	**96,681**	**11.20 (11.12–11.26)**	**36.37 (36.29–36.47)**	**87,461**
	6 days to ≤7 days (***f***)	**11.76 (11.70–11.90)**	**38.68 (38.53–38.80)**	**59,617**	**11.22 (11.13–11.28)**	**36.42 (36.31–36.51)**	**53,315**
	7 days to ≤8 days (***g***)	**11.78 (11.70–11.91)**	**39.12 (38.89–39.30)**	**33,928**	**11.28 (11.17–11.39)**	**37.26 (37.10–37.38)**	**29,758**
	8 days to ≤9 days (***h***)	**12.09 (11.95–11.20)**	**39.70 (39.54–39.86)**	**25,400**	**11.67 (11.56–11.82)**	**37.88 (37.67–38.11)**	**22,199**
	9 days to ≤10 days (***i***)	**12.07 (11.91–11.14)**	**40.35 (40.15–40.59)**	**19,846**	**11.52 (11.36–11.64)**	**38.46 (38.26–38.75)**	**17,339**
	10 days to ≤11 days (***j***)	**12.14 (11.96–12.29)**	**40.88 (40.68–41.12)**	**15,313**	**11.80 (11.60–11.98)**	**39.07 (38.87–39.33)**	**13,330**
	11 days to ≤12 days (***k***)	**12.35 (12.19–12.48)**	**41.02 (40.75–41.22)**	**14,104**	**12.15 (11.96–12.31)**	**39.69 (39.33–39.98)**	**12,284**
	12 days to ≤13 days (***L***)	**12.71 (12.52–12.89)**	**41.39 (41.04–41.69)**	**12,475**	**12.56 (12.27–12.83)**	**39.89 (39.66–40.14)**	**11,122**
	13 days to ≤14 days (***m***)	**12.97 (12.78–13.20)**	**41.95 (41.71–42.33)**	**11,464**	**12.83 (12.65–13.17)**	**40.46 (40.18–40.77)**	**10,097**
C2	0 to ≤1 day (***a***)	**9.38 (9.21–9.53)**	**31.23 (31.00–31.49)**	**10,275**	**8.90 (8.70–9.07)**	**28.44 (28.17–28.74)**	**9,550**
	1 day to ≤2 days (***b***)	**9.72 (9.66–9.77)**	**32.83 (32.77–32.91)**	**115,029**	**8.99 (8.93–9.04)**	**30.43 (30.34–30.51)**	**105,003**
	2 days to ≤3 days (***c***)	**8.78 (8.75–8.80)**	**32.74 (32.71–32.76)**	**1,045,982**	**8.17 (8.15–8.19)**	**30.42 (30.40–30.45)**	**960,681**
	3 days to ≤4 days (***d***)	**8.41 (8.39–8.43)**	**30.77 (30.75–30.80)**	**797,123**	**7.91 (7.89–7.93)**	**28.87 (28.84–28.90)**	**727,829**
	4 days to ≤5 days (***e***)	**7.35 (7.30–7.39)**	**26.91 (26.85–26.97)**	**158,368**	**7.00 (6.96–7.02)**	**25.43 (25.38–25.49)**	**143,392**
	5 days to ≤6 days (***f***)	**6.61 (6.56–6.67)**	**24.29 (24.22–24.34)**	**96,345**	**6.37 (6.31–6.42)**	**23.15 (23.09–23.20)**	**86,967**
	6 days to ≤7 days (***g***)	**5.99 (5.97–5.04)**	**21.82 (21.77–21.90)**	**59,143**	**5.83 (5.77–5.88)**	**20.89 (20.84–20.95)**	**52,725**
	7 days to ≤8 days (***h***)	**5.43 (5.34–5.52)**	**20.25 (20.14–20.32)**	**33,407**	**5.31 (5.25–5.37)**	**19.53 (19.45–19.61)**	**29,210**
	8 days to ≤9 days (***i***)	**5.06 (5.01–5.12)**	**19.00 (18.92–19.08)**	**24,792**	**5.00 (4.94–5.06)**	**18.53 (18.41–18.62)**	**21,616**
	9 days to ≤10 days (***j***)	**4.92 (4.84–5.00)**	**18.43 (18.32–18.54)**	**19,295**	**4.85 (4.78–4.92)**	**17.50 (17.36–17.60)**	**16,762**
	10 days to ≤11 days (***k***)	**4.82 (4.73–4.95)**	**17.67 (17.54–17.80)**	**14,819**	**4.70 (4.59–4.75)**	**17.05 (16.92–17.13)**	**12,811**
	11 days to ≤12 days (***L***)	**4.79 (4.70–4.85)**	**17.38 (17.28–17.52)**	**13,642**	**4.70 (4.61–4.81)**	**16.61 (16.49–16.75)**	**11,785**
	12 days to ≤13 days (***kl***)	**4.92 (4.84–5.03)**	**17.44 (17.33–17.55)**	**12,108**	**4.64 (4.50–4.71)**	**16.43 (16.26–16.57)**	**10,674**
	13 days to ≤14 days (***kl***)	**4.96 (4.90–5.06)**	**17.34 (17.24–17.47)**	**11,127**	**4.78 (4.64–4.91)**	**16.76 (16.65–16.89)**	**9,741**
C3	0 to ≤1 day (***a***)	**0.79 (0.78–0.80)**	**2.82 (2.80–2.84)**	**10,304**	**0.77 (0.76–0.79)**	**2.66 (2.63–2.67)**	**9,551**
	1 day to ≤2 days (***a***)	**0.78 (0.78–0.78)**	**2.88 (2.88–2.89)**	**115,866**	**0.74 (0.73–0.74)**	**2.75 (2.75–2.76)**	**105,326**
	2 days to ≤3 days (***b***)	**0.77 (0.77–0.77)**	**2.95 (2.95–2.95)**	**1,052,696**	**0.74 (0.74–0.74)**	**2.84 (2.83–2.84)**	**964,210**
	3 days to ≤4 days (***c***)	**0.75 (0.75–0.76)**	**2.78 (2.78–2.78)**	**801,609**	**0.72 (0.72–0.72)**	**2.70 (2.70–2.70)**	**730,749**
	4 days to ≤5 days (***d***)	**0.66 (0.66–0.66)**	**2.44 (2.44–2.45)**	**159,178**	**0.64 (0.64–0.65)**	**2.37 (2.36–2.37)**	**143,979**
	5 days to ≤6 days (***e***)	**0.57 (0.57–0.57)**	**2.12 (2.11–2.13)**	**96,549**	**0.55 (0.54–0.55)**	**2.09 (2.09–2.10)**	**87,072**
	6 days to ≤7 days (***f***)	**0.49 (0.48–0.49)**	**1.88 (1.87–1.88)**	**58,757**	**0.48 (0.48–0.49)**	**1.86 (1.85–1.86)**	**52,232**
	7 days to ≤8 days (***g***)	**0.45 (0.45–0.46)**	**1.77 (1.77–1.78)**	**32,929**	**0.43 (0.42–0.43)**	**1.75 (1.74–1.76)**	**28,637**
	8 days to ≤9 days (***h***)	0.41 (0.41–0.41)	1.68 (1.67–1.68)	45,176	0.41 (0.41–0.41)	1.68 (1.67–1.68)	45,176
	9 days to ≤10 days (***i***)	**0.39 (0.38–0.39)**	**1.69 (1.68–1.70)**	**18,657**	**0.39 (0.39–0.40)**	**1.64 (1.62–1.65)**	**16,211**
	10 days to ≤11 days (***ij***)	0.38 (0.38–0.39)	1.67 (1.66–1.68)	26,677	0.38 (0.38–0.39)	1.67 (1.66–1.68)	26,677
	11 days to ≤12 days (***j***)	0.38 (0.37–0.38)	1.66 (1.65–1.66)	24,589	0.38 (0.37–0.38)	1.66 (1.65–1.66)	24,589
	12 days to ≤13 days (***h***)	0.39 (0.39–0.40)	1.71 (1.70–1.72)	22,153	0.39 (0.39–0.40)	1.71 (1.70–1.72)	22,153
	13 days to ≤14 days (***k***)	0.39 (0.38–0.39)	1.74 (1.73–1.75)	20,156	0.39 (0.38–0.39)	1.74 (1.73–1.75)	20,156
C3-DC + C4-OH	0 to ≤1 day (***a***)	**0.04 (0.03–0.04)**	**0.20 (0.19–0.20)**	**10,361**	**0.04 (0.04–0.04)**	**0.18 (0.18–0.19)**	**9,615**
	1 day to ≤2 days (***b***)	**0.04 (0.04–0.04)**	**0.31 (0.31–0.31)**	**115,192**	**0.04 (0.04–0.04)**	**0.32 (0.32–0.32)**	**101,951**
	2 days to ≤3 days (***c***)	**0.05 (0.05–0.05)**	**0.27 (0.27–0.27)**	**1,052,948**	**0.04 (0.04–0.04)**	**0.26 (0.26–0.26)**	**964,832**
	3 days to ≤4 days (***d***)	**0.04 (0.04–0.04)**	**0.23 (0.23–0.23)**	**801,386**	**0.04 (0.04–0.04)**	**0.22 (0.22–0.22)**	**728,632**
	4 days to ≤5 days (***e***)	**0.04 (0.04–0.04)**	**0.17 (0.17–0.17)**	**158,869**	**0.04 (0.04–0.04)**	**0.17 (0.17–0.17)**	**144,283**
	5 days to ≤6 days (***f***)	**0.04 (0.04–0.04)**	**0.15 (0.15–0.15)**	**96,429**	**0.04 (0.04–0.04)**	**0.15 (0.15–0.15)**	**87,606**
	6 days to ≤7 days (***g***)	0.04 (0.04–0.04)	0.15 (0.15–0.15)	112,850	0.04 (0.04–0.04)	0.15 (0.15–0.15)	112,850
	7 days to ≤8 days (***h***)	**0.04 (0.03–0.04)**	**0.13 (0.13–0.13)**	**33,775**	**0.04 (0.04–0.04)**	**0.13 (0.12–0.13)**	**29,628**
	8 days to ≤9 days (***i***)	0.03 (0.03–0.03)	0.13 (0.13–0.13)	47,262	0.03 (0.03–0.03)	0.13 (0.13–0.13)	47,262
	9 days to ≤10 days (***j***)	0.03 (0.03–0.03)	0.13 (0.13–0.13)	36,809	0.03 (0.03–0.03)	0.13 (0.13–0.13)	36,809
	10 days to ≤11 days (***k***)	**0.03 (0.03–0.03)**	**0.11 (0.11–0.11)**	**15,189**	**0.03 (0.03–0.03)**	**0.11 (0.11–0.11)**	**13,177**
	11 days to ≤12 days (***L***)	0.03 (0.03–0.03)	0.11 (0.11–0.11)	26,087	0.03 (0.03–0.03)	0.11 (0.11–0.11)	26,087
	12 days to ≤13 days (***m***)	0.03 (0.03–0.03)	0.11 (0.11–0.11)	23,335	0.03 (0.03–0.03)	0.11 (0.11–0.11)	23,335
	13 days to ≤14 days (***n***)	0.03 (0.03–0.03)	0.10 (0.10–0.10)	21,301	0.03 (0.03–0.03)	0.10 (0.10–0.10)	21,301
C4	0 to ≤1 day (***a***)	**0.11 (0.10–0.11)**	**0.32 (0.32–0.33)**	**10,293**	**0.12 (0.12–0.12)**	**0.33 (0.33–0.34)**	**9,560**
	1 day to ≤2 days (***b***)	**0.07 (0.06–0.07)**	**0.32 (0.32–0.32)**	**111,519**	**0.07 (0.07–0.07)**	**0.33 (0.32–0.33)**	**100,894**
	2 days to ≤3 days (***c***)	**0.10 (0.10–0.10)**	**0.33 (0.33–0.33)**	**1,047,763**	**0.11 (0.11–0.11)**	**0.36 (0.36–0.36)**	**956,213**
	3 days to ≤4 days (***a***)	**0.11 (0.11–0.11)**	**0.32 (0.32–0.32)**	**801,056**	**0.12 (0.12–0.12)**	**0.34 (0.34–0.34)**	**728,565**
	4 days to ≤5 days (***b***)	**0.10 (0.10–0.10)**	**0.30 (0.30–0.30)**	**158,430**	**0.11 (0.11–0.11)**	**0.31 (0.31–0.31)**	**143,525**
	5 days to ≤6 days (***d***)	**0.10 (0.10–0.10)**	**0.28 (0.28–0.28)**	**96,413**	**0.10 (0.10–0.10)**	**0.30 (0.30–0.30)**	**87,284**
	6 days to ≤7 days (***e***)	**0.10 (0.10–0.10)**	**0.28 (0.28–0.28)**	**59,367**	**0.10 (0.10–0.10)**	**0.29 (0.29–0.29)**	**53,174**
	7 days to ≤8 days (***f***)	**0.09 (0.09–0.09)**	**0.27 (0.27–0.27)**	**33,637**	**0.10 (0.10–0.10)**	**0.28 (0.28–0.28)**	**29,614**
	8 days to ≤9 days (***g***)	**0.09 (0.09–0.09)**	**0.27 (0.27–0.27)**	**25,125**	**0.09 (0.09–0.09)**	**0.28 (0.28–0.28)**	**21,984**
	9 days to ≤10 days (***h***)	**0.09 (0.09–0.09)**	**0.27 (0.27–0.28)**	**19,611**	**0.09 (0.09–0.09)**	**0.27 (0.27–0.27)**	**17,173**
	10 days to ≤11 days (***i***)	**0.09 (0.09–0.09)**	**0.26 (0.26–0.26)**	**15,141**	**0.09 (0.09–0.09)**	**0.27 (0.27–0.27)**	**13,189**
	11 days to ≤12 days (***j***)	**0.09 (0.09–0.09)**	**0.26 (0.26–0.26)**	**13,931**	**0.09 (0.09–0.09)**	**0.27 (0.27–0.28)**	**12,155**
	12 days to ≤13 days (***k***)	**0.09 (0.09–0.09)**	**0.26 (0.26–0.27)**	**12,343**	**0.09 (0.09–0.09)**	**0.27 (0.27–0.28)**	**11,034**
	13 days to ≤14 days (***jk***)	**0.09 (0.09–0.09)**	**0.26 (0.26–0.27)**	**11,332**	**0.10 (0.10–0.11)**	**0.26 (0.25–0.26)**	**10,025**
C4-DC + C5-OH	0 to ≤1 day (***a***)	**0.11 (0.11–0.11)**	**0.29 (0.28–0.29)**	**10,303**	**0.10 (0.10–0.10)**	**0.28 (0.28–0.28)**	**9,575**
	1 day to ≤2 days (***b***)	**0.06 (0.06–0.06)**	**0.30 (0.30–0.30)**	**108,787**	**0.07 (0.07–0.07)**	**0.28 (0.28–0.28)**	**100,757**
	2 days to ≤3 days (***c***)	**0.10 (0.10–0.10)**	**0.33 (0.33–0.33)**	**1,049,969**	**0.10 (0.10–0.10)**	**0.30 (0.30–0.30)**	**963,151**
	3 days to ≤4 days (***d***)	**0.11 (0.11–0.11)**	**0.32 (0.32–0.32)**	**801,080**	**0.11 (0.11–0.11)**	**0.30 (0.30–0.30)**	**730,521**
	4 days to ≤5 days (***e***)	**0.11 (0.11–0.11)**	**0.30 (0.30–0.30)**	**158,757**	**0.10 (0.10–0.10)**	**0.28 (0.28–0.28)**	**143,787**
	5 days to ≤6 days (***a***)	**0.10 (0.10–0.11)**	**0.30 (0.30–0.30)**	**96,669**	**0.10 (0.10–0.10)**	**0.28 (0.28–0.28)**	**87,355**
	6 days to ≤7 days (***f***)	**0.10 (0.10–0.10)**	**0.29 (0.29–0.29)**	**59,476**	**0.10 (0.10–0.10)**	**0.28 (0.28–0.28)**	**53,122**
	7 days to ≤8 days (***g***)	**0.10 (0.10–0.10)**	**0.28 (0.28–0.28)**	**33,706**	**0.10 (0.10–0.10)**	**0.27 (0.27–0.27)**	**29,588**
	8 days to ≤9 days (***g***)	**0.10 (0.10–0.10)**	**0.28 (0.28–0.28)**	**25,164**	**0.10 (0.10–0.10)**	**0.27 (0.27–0.27)**	**21,989**
	9 days to ≤10 days (***g***)	**0.10 (0.10–0.10)**	**0.28 (0.28–0.28)**	**19,612**	**0.10 (0.10–0.10)**	**0.27 (0.27–0.27)**	**17,152**
	10 days to ≤11 days (***hi***)	**0.10 (0.10–0.10)**	**0.28 (0.28–0.28)**	**15,168**	**0.10 (0.10–0.10)**	**0.27 (0.27–0.27)**	**13,167**
	11 days to ≤12 days (***h***)	**0.10 (0.10–0.10)**	**0.28 (0.28–0.28)**	**13,969**	**0.10 (0.10–0.10)**	**0.27 (0.27–0.27)**	**12,134**
	12 days to ≤13 days (***i***)	**0.10 (0.10–0.10)**	**0.28 (0.28–0.28)**	**12,350**	**0.10 (0.10–0.10)**	**0.27 (0.27–0.27)**	**10,987**
	13 days to ≤14 days (***hi***)	**0.10 (0.10–0.10)**	**0.28 (0.28–0.28)**	**11,365**	**0.10 (0.10–0.10)**	**0.27 (0.27–0.27)**	**9,939**
C5	0 to ≤1 day (***a***)	**0.04 (0.04–0.04)**	**0.15 (0.15–0.15)**	**10,356**	**0.04 (0.03–0.04)**	**0.15 (0.15–0.15)**	**9,606**
	1 day to ≤2 days (***b***)	0.02 (0.02–0.02)	0.17 (0.17–0.17)	219,252	0.02 (0.02–0.02)	0.18 (0.18–0.18)	219,252
	2 days to ≤3 days (***c***)	**0.05 (0.05–0.05)**	**0.17 (0.17–0.17)**	**1,059,324**	**0.04 (0.04–0.04)**	**0.18 (0.18–0.18)**	**968,680**
	3 days to ≤4 days (***d***)	**0.06 (0.06–0.06)**	**0.17 (0.17–0.17)**	**804,862**	**0.06 (0.06–0.06)**	**0.18 (0.18–0.18)**	**732,769**
	4 days to ≤5 days (***e***)	**0.06 (0.06–0.06)**	**0.18 (0.18–0.18)**	**158,623**	**0.06 (0.06–0.06)**	**0.20 (0.20–0.20)**	**143,687**
	5 days to ≤6 days (***f***)	**0.06 (0.06–0.06)**	**0.20 (0.20–0.20)**	**96,317**	**0.06 (0.06–0.06)**	**0.21 (0.21–0.21)**	**86,996**
	6 days to ≤7 days (***g***)	**0.06 (0.06–0.06)**	**0.22 (0.22–0.22)**	**59,235**	**0.06 (0.06–0.06)**	**0.23 (0.23–0.23)**	**52,971**
	7 days to ≤8 days (***h***)	**0.06 (0.06–0.06)**	**0.23 (0.23–0.23)**	**33,533**	**0.06 (0.06–0.06)**	**0.23 (0.23–0.23)**	**29,408**
	8 days to ≤9 days (***i***)	**0.06 (0.06–0.06)**	**0.23 (0.23–0.23)**	**25,025**	**0.06 (0.06–0.06)**	**0.25 (0.25–0.25)**	**21,907**
	9 days to ≤10 days (***j***)	**0.06 (0.06–0.06)**	**0.25 (0.25–0.25)**	**19,534**	**0.06 (0.05–0.06)**	**0.25 (0.25–0.26)**	**17,081**
	10 days to ≤11 days (***k***)	**0.06 (0.06–0.06)**	**0.25 (0.25–0.25)**	**15,051**	**0.06 (0.06–0.06)**	**0.26 (0.26–0.26)**	**13,089**
	11 days to ≤12 days (***L***)	**0.06 (0.06–0.06)**	**0.26 (0.26–0.26)**	**13,795**	**0.07 (0.07–0.08)**	**0.26 (0.26–0.26)**	**12,014**
	12 days to ≤13 days (***m***)	**0.06 (0.06–0.06)**	**0.26 (0.26–0.26)**	**12,242**	**0.07 (0.07–0.08)**	**0.26 (0.26–0.26)**	**10,889**
	13 days to ≤14 days (***n***)	**0.06 (0.06–0.06)**	**0.26 (0.26–0.26)**	**11,200**	**0.07 (0.07–0.07)**	**0.28 (0.28–0.28)**	**9,884**
C5-DC + C6-OH	0 to ≤1 day (***a***)	**0.06 (0.06–0.06)**	**0.19 (0.19–0.19)**	**10,339**	**0.06 (0.06–0.06)**	**0.18 (0.18–0.18)**	**9,601**
	1 day to ≤2 days (***b***)	**0.06 (0.06–0.06)**	**0.25 (0.25–0.25)**	**114,253**	**0.06 (0.06–0.06)**	**0.24 (0.24–0.24)**	**104,304**
	2 days to ≤3 days (***c***)	**0.06 (0.06–0.06)**	**0.20 (0.20–0.20)**	**1,055,950**	**0.06 (0.06–0.06)**	**0.19 (0.19–0.19)**	**966,691**
	3 days to ≤4 days (***d***)	**0.06 (0.06–0.06)**	**0.18 (0.18–0.18)**	**803,479**	**0.05 (0.05–0.05)**	**0.18 (0.18–0.18)**	**731,895**
	4 days to ≤5 days (***e***)	**0.05 (0.05–0.05)**	**0.15 (0.15–0.15)**	**159,126**	**0.05 (0.05–0.05)**	**0.15 (0.15–0.15)**	**144,036**
	5 days to ≤6 days (***f***)	**0.05 (0.05–0.05)**	**0.14 (0.14–0.14)**	**96,757**	**0.05 (0.04–0.05)**	**0.13 (0.13–0.14)**	**87,385**
	6 days to ≤7 days (***g***)	**0.04 (0.04–0.04)**	**0.13 (0.13–0.13)**	**59,424**	**0.04 (0.04–0.04)**	**0.13 (0.13–0.13)**	**53,039**
	7 days to ≤8 days (***h***)	**0.04 (0.04–0.04)**	**0.14 (0.14–0.14)**	**33,691**	**0.04 (0.04–0.04)**	**0.14 (0.14–0.14)**	**29,509**
	8 days to ≤9 days (***i***)	**0.04 (0.04–0.04)**	**0.12 (0.12–0.13)**	**25,127**	**0.04 (0.04–0.04)**	**0.12 (0.12–0.12)**	**21,916**
	9 days to ≤10 days (***j***)	**0.04 (0.04–0.04)**	**0.12 (0.12–0.12)**	**19,547**	**0.04 (0.04–0.04)**	**0.12 (0.12–0.12)**	**17,068**
	10 days to ≤11 days (***k***)	**0.04 (0.04–0.04)**	**0.12 (0.12–0.12)**	**15,112**	**0.04 (0.04–0.04)**	**0.12 (0.12–0.12)**	**13,100**
	11 days to ≤12 days (***L***)	**0.04 (0.04–0.04)**	**0.12 (0.12–0.12)**	**13,881**	**0.04 (0.04–0.04)**	**0.12 (0.12–0.12)**	**12,072**
	12 days to ≤13 days (***L***)	**0.04 (0.04–0.04)**	**0.12 (0.12–0.12)**	**12,279**	**0.04 (0.04–0.04)**	**0.12 (0.12–0.12)**	**10,905**
	13 days to ≤14 days (***m***)	0.04 (0.04–0.04)	0.12 (0.12–0.12)	21,154	0.04 (0.04–0.04)	0.12 (0.12–0.12)	21,154
C6	0 to ≤1 day (***a***)	**0.02 (0.02–0.02)**	**0.08 (0.08–0.08)**	**10,143**	**0.03 (0.03–0.03)**	**0.06 (0.06–0.06)**	**8,010**
	1 day to ≤2 days (***b***)	**0.02 (0.02–0.02)**	**0.08 (0.08–0.08)**	**113,352**	**0.02 (0.02–0.02)**	**0.08 (0.08–0.08)**	**104,220**
	2 days to ≤3 days (***c***)	**0.02 (0.02–0.02)**	**0.08 (0.08–0.08)**	**1,033,512**	**0.02 (0.02–0.02)**	**0.07 (0.07–0.07)**	**954,201**
	3 days to ≤4 days (***d***)	**0.02 (0.02–0.02)**	**0.07 (0.07–0.07)**	**793,074**	**0.02 (0.02–0.02)**	**0.07 (0.07–0.07)**	**727,096**
	4 days to ≤5 days (***e***)	**0.02 (0.02–0.02)**	**0.05 (0.05–0.05)**	**144,606**	**0.02 (0.02–0.02)**	**0.05 (0.05–0.05)**	**132,721**
	5 days to ≤6 days (***f***)	**0.02 (0.02–0.02)**	**0.05 (0.05–0.05)**	**89,849**	**0.01 (0.01–0.01)**	**0.06 (0.06–0.06)**	**87,561**
	6 days to ≤7 days (***g***)	**0.01 (0.01–0.01)**	**0.06 (0.06–0.06)**	**58,977**	**0.01 (0.01–0.01)**	**0.06 (0.06–0.06)**	**53,389**
	7 days to ≤8 days (***h***)	**0.01 (0.01–0.01)**	**0.06 (0.06–0.06)**	**33,574**	**0.01 (0.01–0.01)**	**0.06 (0.06–0.06)**	**29,805**
	8 days to ≤9 days (***i***)	**0.01 (0.01–0.01)**	**0.06 (0.06–0.06)**	**25,039**	**0.01 (0.01–0.01)**	**0.06 (0.06–0.06)**	**22,213**
	9 days to ≤10 days (***f***)	**0.02 (0.02–0.02)**	**0.05 (0.05–0.05)**	**18,278**	**0.01 (0.01–0.01)**	**0.06 (0.06–0.06)**	**17,179**
	10 days to ≤11 days (***k***)	**0.02 (0.02–0.02)**	**0.05 (0.05–0.05)**	**14,161**	**0.02 (0.02–0.02)**	**0.05 (0.05–0.05)**	**12,347**
	11 days to ≤12 days (***L***)	**0.02 (0.02–0.02)**	**0.05 (0.05–0.05)**	**12,985**	**0.02 (0.02–0.02)**	**0.05 (0.05–0.05)**	**11,326**
	12 days to ≤13 days (***em***)	**0.02 (0.02–0.02)**	**0.05 (0.05–0.05)**	**11,464**	**0.02 (0.02–0.02)**	**0.05 (0.05–0.05)**	**10,311**
	13 days to ≤14 days (***m***)	**0.02 (0.02–0.02)**	**0.05 (0.05–0.05)**	**10,538**	**0.02 (0.02–0.02)**	**0.05 (0.05–0.05)**	**9,339**
C6-DC	0 to ≤1 day (***a***)	**0.04 (0.04–0.04)**	**0.16 (0.16–0.17)**	**10,343**	**0.04 (0.04–0.04)**	**0.18 (0.18–0.19)**	**9,603**
	1 day to ≤2 days (***b***)	**0.04 (0.04–0.05)**	**0.23 (0.23–0.23)**	**116,601**	**0.05 (0.04–0.05)**	**0.24 (0.24–0.24)**	**105,787**
	2 days to ≤3 days (***c***)	**0.04 (0.04–0.04)**	**0.18 (0.18–0.18)**	**1,060,248**	**0.04 (0.04–0.04)**	**0.20 (0.20–0.20)**	**968,910**
	3 days to ≤4 days (***d***)	**0.04 (0.04–0.04)**	**0.19 (0.19–0.19)**	**805,997**	**0.04 (0.04–0.04)**	**0.19 (0.19–0.19)**	**733,592**
	4 days to ≤5 days (***e***)	**0.03 (0.03–0.03)**	**0.16 (0.16–0.16)**	**159,403**	**0.03 (0.03–0.03)**	**0.16 (0.16–0.16)**	**144,247**
	5 days to ≤6 days (***f***)	**0.03 (0.03–0.03)**	**0.14 (0.14–0.14)**	**96,992**	**0.03 (0.03–0.03)**	**0.14 (0.14–0.14)**	**87,562**
	6 days to ≤7 days (***g***)	**0.03 (0.03–0.03)**	**0.14 (0.14–0.14)**	**59,733**	**0.03 (0.03–0.03)**	**0.14 (0.14–0.14)**	**53,346**
	7 days to ≤8 days (***h***)	**0.03 (0.03–0.03)**	**0.13 (0.13–0.13)**	**34,006**	**0.03 (0.03–0.03)**	**0.14 (0.14–0.14)**	**29,801**
	8 days to ≤9 days (***g***)	0.03 (0.03–0.03)	0.14 (0.14–0.14)	47,671	0.03 (0.03–0.03)	0.14 (0.14–0.14)	47,671
	9 days to ≤10 days (***f***)	0.03 (0.03–0.03)	0.14 (0.14–0.14)	37,286	0.03 (0.03–0.03)	0.14 (0.14–0.14)	37,286
	10 days to ≤11 days (***i***)	0.03 (0.03–0.03)	0.14 (0.14–0.14)	28,741	0.03 (0.03–0.03)	0.14 (0.14–0.14)	28,741
	11 days to ≤12 days (***jk***)	0.03 (0.03–0.03)	0.14 (0.14–0.14)	26,452	0.03 (0.03–0.03)	0.14 (0.14–0.14)	26,452
	12 days to ≤13 days (***j***)	0.03 (0.03–0.03)	0.14 (0.14–0.14)	23,708	0.03 (0.03–0.03)	0.14 (0.14–0.14)	23,708
	13 days to ≤14 days (***k***)	0.03 (0.03–0.03)	0.14 (0.14–0.14)	21,671	0.03 (0.03–0.03)	0.14 (0.14–0.14)	21,671
C8	0 to ≤1 day (***a***)	**0.03 (0.03–0.03)**	**0.10 (0.10–0.10)**	**10,324**	**0.03 (0.03–0.03)**	**0.08 (0.08–0.09)**	**9,600**
	1 day to ≤2 days (***b***)	**0.03 (0.03–0.03)**	**0.10 (0.10–0.10)**	**116,325**	**0.03 (0.03–0.03)**	**0.10 (0.10–0.10)**	**105,728**
	2 days to ≤3 days (***c***)	**0.03 (0.03–0.03)**	**0.10 (0.10–0.10)**	**1,057,935**	**0.03 (0.03–0.03)**	**0.10 (0.10–0.10)**	**968,607**
	3 days to ≤4 days (***d***)	**0.03 (0.03–0.03)**	**0.10 (0.10–0.10)**	**803,824**	**0.02 (0.02–0.02)**	**0.09 (0.09–0.09)**	**732,597**
	4 days to ≤5 days (***e***)	**0.02 (0.02–0.02)**	**0.08 (0.08–0.08)**	**159,554**	**0.02 (0.02–0.02)**	**0.08 (0.08–0.09)**	**144,322**
	5 days to ≤6 days (***f***)	**0.02 (0.02–0.02)**	**0.07 (0.07–0.07)**	**97,166**	**0.02 (0.02–0.02)**	**0.07 (0.07–0.07)**	**87,734**
	6 days to ≤7 days (***g***)	**0.02 (0.02–0.02)**	**0.07 (0.07–0.07)**	**59,908**	**0.02 (0.02–0.02)**	**0.07 (0.07–0.07)**	**53,429**
	7 days to ≤8 days (***g***)	**0.02 (0.02–0.02)**	**0.07 (0.07–0.07)**	**34,094**	**0.02 (0.02–0.02)**	**0.07 (0.07–0.07)**	**29,832**
	8 days to ≤9 days (***g***)	**0.02 (0.02–0.02)**	**0.07 (0.07–0.07)**	**25,516**	**0.02 (0.02–0.02)**	**0.07 (0.07–0.07)**	**22,257**
	9 days to ≤10 days (*h*)	**0.02 (0.02–0.02)**	**0.07 (0.07–0.07)**	**19,960**	**0.02 (0.02–0.02)**	**0.07 (0.07–0.07)**	**17,392**
	10 days to ≤11 days (***h***)	**0.02 (0.02–0.02)**	**0.07 (0.07–0.07)**	**15,406**	**0.02 (0.02–0.02)**	**0.07 (0.07–0.07)**	**13,368**
	11 days to ≤12 days (***h***)	**0.02 (0.02–0.02)**	**0.07 (0.07–0.07)**	**14,163**	**0.02 (0.02–0.02)**	**0.07 (0.07–0.07)**	**12,303**
	12 days to ≤13 days (***h***)	**0.02 (0.02–0.02)**	**0.07 (0.07–0.07)**	**12,542**	**0.02 (0.02–0.02)**	**0.07 (0.07–0.07)**	**11,145**
	13 days to ≤14 days (***h***)	**0.02 (0.02–0.02)**	**0.07 (0.07–0.07)**	**11,526**	**0.02 (0.02–0.02)**	**0.07 (0.07–0.07)**	**10,134**
C8:1	0 to ≤1 day (***a***)	**0.05 (0.05–0.05)**	**0.21 (0.21–0.21)**	**10,269**	**0.05 (0.05–0.05)**	**0.21 (0.21–0.21)**	**9,523**
	1 day to ≤2 days (***b***)	**0.04 (0.04–0.04)**	**0.24 (0.24–0.24)**	**115,199**	**0.04 (0.04–0.04)**	**0.22 (0.22–0.22)**	**104,687**
	2 days to ≤3 days (***c***)	**0.05 (0.05–0.05)**	**0.23 (0.23–0.23)**	**1,055,471**	**0.05 (0.05–0.05)**	**0.24 (0.24–0.25)**	**965,965**
	3 days to ≤4 days (***d***)	**0.06 (0.06–0.06)**	**0.24 (0.23–0.24)**	**802,066**	**0.06 (0.06–0.06)**	**0.23 (0.23–0.23)**	**730,911**
	4 days to ≤5 days (***e***)	**0.05 (0.05–0.05)**	**0.23 (0.23–0.23)**	**158,586**	**0.05 (0.05–0.05)**	**0.22 (0.22–0.22)**	**143,623**
	5 days to ≤6 days (***f***)	0.04 (0.04–0.04)	0.23 (0.23–0.24)	182,932	0.04 (0.04–0.04)	0.23 (0.23–0.24)	182,932
	6 days to ≤7 days (***a***)	0.04 (0.04–0.04)	0.22 (0.21–0.22)	111,825	0.04 (0.04–0.04)	0.22 (0.21–0.22)	111,825
	7 days to ≤8 days (***a***)	0.04 (0.04–0.04)	0.23 (0.23–0.23)	62,787	0.04 (0.04–0.04)	0.23 (0.23–0.23)	62,787
	8 days to ≤9 days (***f***)	0.04 (0.04–0.04)	0.23 (0.23–0.23)	46,869	0.04 (0.04–0.04)	0.23 (0.23–0.23)	46,869
	9 days to ≤10 days (***b***)	**0.04 (0.04–0.04)**	**0.25 (0.25–0.25)**	**19,554**	**0.04 (0.04–0.04)**	**0.25 (0.25–0.25)**	**17,054**
	10 days to ≤11 days (***g***)	0.04 (0.04–0.04)	0.25 (0.25–0.26)	28,200	0.04 (0.04–0.04)	0.25 (0.25–0.26)	28,200
	11 days to ≤12 days (***g***)	**0.04 (0.04–0.04)**	**0.25 (0.24–0.25)**	**13,877**	**0.04 (0.04–0.04)**	**0.26 (0.26–0.26)**	**12,012**
	12 days to ≤13 days (***h***)	0.04 (0.04–0.04)	0.26 (0.26–0.26)	23,097	0.04 (0.04–0.04)	0.26 (0.26–0.26)	23,097
	13 days to ≤14 days (***i***)	0.04 (0.04–0.04)	0.26 (0.25–0.26)	21,109	0.04 (0.04–0.04)	0.26 (0.25–0.26)	21,109
C10	0 to ≤1 day (***a***)	**0.04 (0.04–0.04)**	**0.15 (0.15–0.15)**	**10,299**	**0.04 (0.04–0.05)**	**0.13 (0.13–0.14)**	**9,576**
	1 day to ≤2 days (***b***)	**0.03 (0.03–0.03)**	**0.15 (0.15–0.15)**	**115,637**	**0.03 (0.03–0.03)**	**0.15 (0.15–0.15)**	**105,020**
	2 days to ≤3 days (***c***)	**0.03 (0.03–0.03)**	**0.15 (0.15–0.15)**	**1,049,789**	**0.03 (0.03–0.03)**	**0.14 (0.14–0.14)**	**960,217**
	3 days to ≤4 days (***d***)	**0.03 (0.03–0.03)**	**0.14 (0.14–0.14)**	**796,974**	**0.03 (0.03–0.03)**	**0.14 (0.14–0.14)**	**725,189**
	4 days to ≤5 days (***e***)	**0.03 (0.03–0.03)**	**0.11 (0.10–0.11)**	**157,236**	**0.03 (0.03–0.03)**	**0.10 (0.10–0.10)**	**141,458**
	5 days to ≤6 days (***f***)	**0.03 (0.03–0.03)**	**0.10 (0.10–0.10)**	**95,382**	**0.02 (0.02–0.03)**	**0.09 (0.09–0.09)**	**85,776**
	6 days to ≤7 days (***g***)	**0.03 (0.03–0.03)**	**0.09 (0.09–0.09)**	**58,696**	**0.02 (0.02–0.02)**	**0.09 (0.09–0.09)**	**52,091**
	7 days to ≤8 days (***hi***)	**0.02 (0.02–0.03)**	**0.09 (0.09–0.09)**	**33,339**	**0.02 (0.02–0.02)**	**0.09 (0.09–0.09)**	**29,119**
	8 days to ≤9 days (***ij***)	**0.03 (0.02–0.03)**	**0.09 (0.09–0.09)**	**24,960**	**0.02 (0.02–0.02)**	**0.09 (0.09–0.09)**	**21,633**
	9 days to ≤10 days (***h***)	**0.03 (0.02–0.03)**	**0.09 (0.09–0.09)**	**19,521**	**0.02 (0.02–0.02)**	**0.09 (0.09–0.09)**	**16,947**
	10 days to ≤11 days (***hi***)	**0.03 (0.02–0.03)**	**0.09 (0.09–0.09)**	**15,069**	**0.03 (0.02–0.03)**	**0.09 (0.09–0.10)**	**13,070**
	11 days to ≤12 days (***hi***)	**0.02 (0.02–0.02)**	**0.09 (0.09–0.09)**	**13,822**	**0.02 (0.02–0.02)**	**0.09 (0.09–0.10)**	**11,974**
	12 days to ≤13 days (***hij***)	**0.03 (0.03–0.03)**	**0.09 (0.09–0.09)**	**12,301**	**0.02 (0.02–0.02)**	**0.09 (0.08–0.09)**	**10,882**
	13 days to ≤14 days (***j***)	**0.03 (0.02–0.03)**	**0.09 (0.09–0.10)**	**11,279**	**0.03 (0.02–0.03)**	**0.09 (0.09–0.10)**	**9,930**
C10:1	0 to ≤1 day (***a***)	**0.04 (0.04–0.05)**	**0.11 (0.11–0.11)**	**10,349**	**0.03 (0.03–0.03)**	**0.11 (0.11–0.11)**	**9,616**
	1 day to ≤2 days (***b***)	**0.03 (0.03–0.03)**	**0.13 (0.13–0.13)**	**112,727**	**0.03 (0.03–0.03)**	**0.14 (0.14–0.14)**	**105,727**
	2 days to ≤3 days (***c***)	**0.03 (0.03–0.03)**	**0.13 (0.13-0.13)**	**1,051,125**	**0.03 (0.03-0.03)**	**0.13 (0.13-0.13)**	**970,613**
	3 days to ≤4 days (***d***)	**0.03 (0.03-0.03)**	**0.14 (0.14-0.14)**	**802,987**	**0.03 (0.03-0.03)**	**0.14 (0.14-0.14)**	**733,977**
	4 days to ≤5 days (***e***)	**0.03 (0.03-0.03)**	**0.13 (0.13-0.13)**	**158,657**	**0.03 (0.03-0.03)**	**0.11 (0.11-0.11)**	**144,566**
	5 days to ≤6 days (***f***)	**0.03 (0.03-0.03)**	**0.11 (0.11-0.11)**	**96,022**	**0.03 (0.03-0.03)**	**0.12 (0.12-0.12)**	**87,892**
	6 days to ≤7 days (***g***)	**0.03 (0.03-0.03)**	**0.12 (0.12-0.12)**	**59,493**	**0.02 (0.02-0.02)**	**0.11 (0.11-0.12)**	**53,565**
	7 days to ≤8 days (***hi***)	**0.02 (0.02-0.02)**	**0.10 (0.10-0.10)**	**33,671**	**0.02 (0.02-0.02)**	**0.10 (0.10-0.10)**	**29,919**
	8 days to ≤9 days (***h***)	**0.02 (0.02-0.02)**	**0.10 (0.10-0.10)**	**24,756**	**0.02 (0.02-0.02)**	**0.10 (0.10-0.10)**	**22,303**
	9 days to ≤10 days (***hi***)	**0.02 (0.02-0.02)**	**0.10 (0.10-0.10)**	**19,493**	**0.02 (0.02-0.02)**	**0.10 (0.10-0.10)**	**17,439**
	10 days to ≤11 days (***ij***)	**0.02 (0.02-0.02)**	**0.10 (0.10-0.10)**	**14,935**	**0.02 (0.02-0.02)**	**0.10 (0.09-0.10)**	**13,399**
	11 days to ≤12 days (***j***)	**0.02 (0.02-0.03)**	**0.10 (0.10-0.10)**	**13,697**	**0.02 (0.02-0.02)**	**0.10 (0.10-0.10)**	**12,332**
	12 days to ≤13 days (***j***)	**0.03 (0.03-0.03)**	**0.10 (0.10-0.10)**	**12,566**	**0.03 (0.02-0.03)**	**0.10 (0.10-0.10)**	**11,189**
	13 days to ≤14 days (***j***)	0.02 (0.02–0.02)	0.10 (0.10–0.10)	21,706	0.02 (0.02–0.02)	0.10 (0.10–0.10)	21,706
C12	0 to ≤1 day (***a***)	**0.04 (0.04-0.04)**	**0.19 (0.18-0.19)**	**10,341**	**0.04 (0.04-0.04)**	**0.15 (0.14-0.15)**	**9,587**
	1 day to ≤2 days (***b***)	**0.04 (0.04-0.04)**	**0.18 (0.18-0.18)**	**116,230**	**0.04 (0.04-0.04)**	**0.17 (0.17-0.17)**	**105,548**
	2 days to ≤3 days (***c***)	**0.04 (0.04-0.04)**	**0.17 (0.17-0.17)**	**1,055,419**	**0.03 (0.03-0.03)**	**0.15 (0.15-0.15)**	**965,316**
	3 days to ≤4 days (***d***)	**0.03 (0.03-0.03)**	**0.13 (0.13-0.13)**	**801,741**	**0.03 (0.03-0.03)**	**0.14 (0.14-0.14)**	**729,392**
	4 days to ≤5 days (***e***)	**0.03 (0.03-0.03)**	**0.10 (0.10-0.10)**	**157,447**	**0.03 (0.03-0.03)**	**0.10 (0.10-0.10)**	**141,833**
	5 days to ≤6 days (***f***)	**0.03 (0.03-0.03)**	**0.09 (0.09-0.09)**	**95,410**	**0.03 (0.02-0.03)**	**0.09 (0.09-0.09)**	**85,841**
	6 days to ≤7 days (***g***)	**0.03 (0.03-0.03)**	**0.09 (0.09-0.09)**	**58,559**	**0.02 (0.02-0.02)**	**0.08 (0.08-0.08)**	**52,068**
	7 days to ≤8 days (***h***)	**0.02 (0.02-0.02)**	**0.09 (0.08-0.09)**	**33,098**	**0.02 (0.02-0.02)**	**0.08 (0.08-0.08)**	**28,948**
	8 days to ≤9 days (***i***)	**0.02 (0.02-0.02)**	**0.08 (0.08-0.08)**	**24,736**	**0.02 (0.02-0.02)**	**0.08 (0.08-0.08)**	**21,518**
	9 days to ≤10 days (***j***)	**0.02 (0.02-0.02)**	**0.08 (0.08-0.08)**	**19,303**	**0.02 (0.02-0.02)**	**0.08 (0.08-0.08)**	**16,793**
	10 days to ≤11 days (***k***)	**0.02 (0.02-0.02)**	**0.08 (0.08-0.08)**	**14,861**	**0.02 (0.02-0.02)**	**0.08 (0.08-0.08)**	**12,930**
	11 days to ≤12 days (***L***)	**0.02 (0.02-0.02)**	**0.08 (0.08-0.08)**	**13,662**	**0.02 (0.02-0.02)**	**0.08 (0.08-0.08)**	**11,878**
	12 days to ≤13 days (***m***)	**0.02 (0.02-0.02)**	**0.08 (0.08-0.08)**	**12,142**	**0.03 (0.03-0.03)**	**0.06 (0.06-0.06)**	**10,745**
	13 days to ≤14 days (***n***)	**0.02 (0.02-0.02)**	**0.08 (0.08-0.08)**	**11,085**	**0.03 (0.03-0.03)**	**0.06 (0.06-0.06)**	**9,747**
C12:1	0 to ≤1 day (***a***)	**0.03 (0.03-0.03)**	**0.20 (0.19-0.20)**	**10,288**	**0.03 (0.03-0.04)**	**0.18 (0.17-0.18)**	**9,496**
	1 day to ≤2 days (***b***)	**0.02 (0.02-0.02)**	**0.17 (0.17-0.17)**	**112,482**	**0.02 (0.02-0.02)**	**0.15 (0.15-0.15)**	**100,630**
	2 days to ≤3 days (***c***)	**0.02 (0.02-0.02)**	**0.15 (0.15-0.15)**	**1,010,273**	**0.02 (0.02-0.02)**	**0.13 (0.13-0.13)**	**908,710**
	3 days to ≤4 days (***d***)	**0.02 (0.02-0.02)**	**0.11 (0.11-0.11)**	**724,065**	**0.02 (0.02-0.02)**	**0.11 (0.11-0.11)**	**667,570**
	4 days to ≤5 days (***e***)	**0.01 (0.01-0.01)**	**0.08 (0.08-0.08)**	**145,737**	**0.01 (0.01-0.01)**	**0.08 (0.08-0.08)**	**133,250**
	5 days to ≤6 days (***f***)	**0.01 (0.01-0.01)**	**0.08 (0.08-0.08)**	**92,154**	**0.01 (0.01-0.01)**	**0.08 (0.08-0.08)**	**83,848**
	6 days to ≤7 days (***g***)	**0.01 (0.01-0.01)**	**0.08 (0.08-0.08)**	**58,635**	**0.01 (0.01-0.01)**	**0.07 (0.07-0.07)**	**50,920**
	7 days to ≤8 days (***h***)	**0.01 (0.01-0.01)**	**0.07 (0.07-0.07)**	**32,510**	**0.01 (0.01-0.01)**	**0.07 (0.07-0.07)**	**28,700**
	8 days to ≤9 days (***i***)	**0.01 (0.01-0.01)**	**0.07 (0.07-0.07)**	**24,442**	**0.01 (0.01-0.01)**	**0.07 (0.07-0.07)**	**21,474**
	9 days to ≤10 days (***j***)	**0.01 (0.01-0.01)**	**0.07 (0.07-0.07)**	**19,257**	**0.01 (0.01-0.01)**	**0.07 (0.07-0.07)**	**16,842**
	10 days to ≤11 days (***k***)	0.01 (0.01–0.01)	0.07 (0.07–0.08)	27,891	0.01 (0.01–0.01)	0.07 (0.07–0.08)	27,891
	11 days to ≤12 days (***L***)	**0.01 (0.01-0.01)**	**0.07 (0.07-0.08)**	**13,775**	**0.01 (0.01-0.01)**	**0.06 (0.06-0.06)**	**12,010**
	12 days to ≤13 days (***m***)	0.01 (0.01–0.01)	0.06 (0.06–0.06)	23,078	0.01 (0.01–0.01)	0.06 (0.06–0.06)	23,078
	13 days to ≤14 days (***n***)	0.01 (0.01–0.01)	0.06 (0.06–0.06)	21,236	0.01 (0.01–0.01)	0.06 (0.06–0.06)	21,236
C14	0 to ≤1 day (***a***)	**0.10 (0.09-0.10)**	**0.31 (0.31-0.31)**	**10,276**	**0.10 (0.10-0.11)**	**0.29 (0.29-0.30)**	**9,576**
	1 day to ≤2 days (***b***)	**0.10 (0.10-0.10)**	**0.32 (0.32-0.32)**	**115,584**	**0.09 (0.09-0.09)**	**0.29 (0.29-0.29)**	**105,485**
	2 days to ≤3 days (***c***)	**0.10 (0.10-0.10)**	**0.32 (0.32-0.32)**	**1,051,129**	**0.09 (0.09-0.09)**	**0.27 (0.27-0.27)**	**966,553**
	3 days to ≤4 days (***d***)	**0.10 (0.10-0.10)**	**0.30 (0.30-0.30)**	**802,286**	**0.09 (0.09-0.09)**	**0.26 (0.26-0.26)**	**732,834**
	4 days to ≤5 days (***e***)	**0.08 (0.08-0.08)**	**0.26 (0.26-0.26)**	**159,413**	**0.07 (0.07-0.07)**	**0.24 (0.24-0.24)**	**144,235**
	5 days to ≤6 days (***f***)	**0.08 (0.08-0.08)**	**0.27 (0.27-0.28)**	**96,965**	**0.07 (0.07-0.07)**	**0.24 (0.24-0.24)**	**87,569**
	6 days to ≤7 days (***g***)	**0.07 (0.07-0.07)**	**0.26 (0.26-0.27)**	**59,675**	**0.07 (0.07-0.08)**	**0.23 (0.23-0.23)**	**53,205**
	7 days to ≤8 days (***h***)	**0.06 (0.06-0.07)**	**0.24 (0.24-0.24)**	**33,875**	**0.06 (0.06-0.06)**	**0.21 (0.21-0.21)**	**29,593**
	8 days to ≤9 days (***i***)	**0.06 (0.06-0.06)**	**0.23 (0.23-0.23)**	**25,204**	**0.06 (0.06-0.06)**	**0.20 (0.20-0.20)**	**21,898**
	9 days to ≤10 days (***j***)	**0.06 (0.06-0.06)**	**0.22 (0.22-0.22)**	**19,618**	**0.05 (0.05-0.05)**	**0.19 (0.18-0.19)**	**16,947**
	10 days to ≤11 days (***k***)	**0.05 (0.05-0.05)**	**0.20 (0.20-0.20)**	**15,033**	**0.05 (0.05-0.05)**	**0.19 (0.19-0.20)**	**12,891**
	11 days to ≤12 days (***L***)	**0.05 (0.05-0.05)**	**0.19 (0.19-0.19)**	**13,681**	**0.05 (0.05-0.05)**	**0.17 (0.17-0.17)**	**11,719**
	12 days to ≤13 days (***m***)	**0.05 (0.05-0.05)**	**0.17 (0.17-0.17)**	**12,020**	**0.05 (0.05-0.06)**	**0.16 (0.16-0.16)**	**10,433**
	13 days to ≤14 days (***n***)	**0.05 (0.05-0.05)**	**0.17 (0.16-0.17)**	**10,875**	**0.04 (0.04-0.04)**	**0.15 (0.15-0.15)**	**9,310**
C14:1	0 to ≤1 day (***a***)	**0.05 (0.05-0.05)**	**0.19 (0.19-0.20)**	**10,090**	**0.05 (0.05-0.05)**	**0.18 (0.17-0.18)**	**9,410**
	1 day to ≤2 days (***b***)	**0.04 (0.04-0.04)**	**0.19 (0.19-0.19)**	**112,866**	**0.04 (0.04-0.04)**	**0.18 (0.18-0.18)**	**103,385**
	2 days to ≤3 days (***c***)	**0.04 (0.04-0.04)**	**0.15 (0.15-0.15)**	**1,034,450**	**0.04 (0.04-0.04)**	**0.17 (0.17-0.17)**	**950,964**
	3 days to ≤4 days (***d***)	**0.04 (0.04-0.04)**	**0.15 (0.15-0.15)**	**795,493**	**0.03 (0.03-0.03)**	**0.13 (0.13-0.13)**	**725,717**
	4 days to ≤5 days (***e***)	**0.03 (0.03-0.03)**	**0.10 (0.10-0.10)**	**158,635**	**0.03 (0.03-0.03)**	**0.10 (0.10-0.10)**	**143,346**
	5 days to ≤6 days (***f***)	**0.03 (0.03-0.03)**	**0.09 (0.09-0.09)**	**96,220**	**0.03 (0.03-0.03)**	**0.09 (0.09-0.09)**	**86,643**
	6 days to ≤7 days (***g***)	**0.03 (0.03-0.03)**	**0.08 (0.08-0.08)**	**59,052**	**0.03 (0.03-0.03)**	**0.08 (0.08-0.08)**	**52,528**
	7 days to ≤8 days (***h***)	**0.03 (0.03-0.03)**	**0.08 (0.08-0.08)**	**33,435**	**0.02 (0.02-0.03)**	**0.08 (0.08-0.08)**	**29,189**
	8 days to ≤9 days (***i***)	**0.02 (0.02-0.03)**	**0.08 (0.08-0.08)**	**24,906**	**0.02 (0.02-0.02)**	**0.08 (0.08-0.08)**	**21,667**
	9 days to ≤10 days (***j***)	**0.02 (0.02-0.02)**	**0.08 (0.08-0.08)**	**19,448**	**0.02 (0.02-0.02)**	**0.06 (0.06-0.06)**	**16,852**
	10 days to ≤11 days (***k***)	**0.03 (0.03-0.03)**	**0.06 (0.06-0.06)**	**14,943**	**0.02 (0.02-0.02)**	**0.06 (0.06-0.06)**	**12,905**
	11 days to ≤12 days (***kl***)	**0.03 (0.03-0.03)**	**0.06 (0.06-0.06)**	**13,685**	**0.02 (0.02-0.02)**	**0.07 (0.07-0.07)**	**11,850**
	12 days to ≤13 days (***k***)	**0.02 (0.02-0.02)**	**0.06 (0.06-0.06)**	**12,109**	**0.02 (0.02-0.02)**	**0.07 (0.07-0.07)**	**10,701**
	13 days to ≤14 days (***L***)	0.02 (0.02–0.02)	0.07 (0.07–0.07)	20,766	0.02 (0.02–0.02)	0.07 (0.07–0.07)	20,766
C16	0 to ≤1 day (***a***)	**1.55 (1.53-1.57)**	**4.97 (4.93-5.00)**	**10,319**	**1.50 (1.48-1.53)**	**4.75 (4.71-4.79)**	**9,593**
	1 day to ≤2 days (***b***)	**1.47 (1.46-1.48)**	**5.31 (5.30-5.33)**	**116,204**	**1.38 (1.37-1.39)**	**4.96 (4.95-4.97)**	**105,744**
	2 days to ≤3 days (***c***)	**1.45 (1.45-1.45)**	**5.51 (5.51-5.51)**	**1,054,703**	**1.35 (1.35-1.35)**	**5.14 (5.13-5.14)**	**968,021**
	3 days to ≤4 days (***d***)	**1.33 (1.32-1.33)**	**5.28 (5.27-5.28)**	**802,507**	**1.25 (1.25-1.26)**	**4.94 (4.94-4.95)**	**732,751**
	4 days to ≤5 days (***e***)	**1.09 (1.08-1.09)**	**4.50 (4.49-4.50)**	**159,501**	**1.02 (1.02-1.03)**	**4.20 (4.19-4.21)**	**144,473**
	5 days to ≤6 days (***f***)	**0.97 (0.96-0.97)**	**3.86 (3.85-3.87)**	**97,154**	**0.91 (0.90-0.91)**	**3.61 (3.60-3.62)**	**87,762**
	6 days to ≤7 days (***g***)	**0.87 (0.87-0.88)**	**3.34 (3.33-3.35)**	**59,798**	**0.80 (0.79-0.80)**	**3.10 (3.10-3.12)**	**53,291**
	7 days to ≤8 days (***h***)	**0.77 (0.76-0.78)**	**2.98 (2.97-2.99)**	**33,940**	**0.72 (0.71-0.72)**	**2.76 (2.75-2.77)**	**29,596**
	8 days to ≤9 days (***i***)	**0.70 (0.69-0.70)**	**2.70 (2.69-2.72)**	**25,243**	**0.67 (0.66-0.68)**	**2.50 (2.48-2.52)**	**21,916**
	9 days to ≤10 days (***j***)	**0.64 (0.62-0.64)**	**2.49 (2.48-2.51)**	**19,549**	**0.60 (0.59-0.61)**	**2.27 (2.26-2.28)**	**16,879**
	10 days to ≤11 days (***k***)	**0.59 (0.58-0.60)**	**2.23 (2.22-2.25)**	**14,862**	**0.56 (0.55-0.57)**	**2.06 (2.05-2.07)**	**12,735**
	11 days to ≤12 days (***L***)	**0.55 (0.55-0.56)**	**2.03 (2.01-2.04)**	**13,404**	**0.52 (0.51-0.52)**	**1.86 (1.85-1.88)**	**11,440**
	12 days to ≤13 days (***m***)	**0.51 (0.50-0.52)**	**1.88 (1.87-1.90)**	**11,545**	**0.49 (0.49-0.50)**	**1.71 (1.70-1.72)**	**9,973**
	13 days to ≤14 days (***n***)	**0.48 (0.48-0.49)**	**1.71 (1.70-1.73)**	**10,263**	**0.46 (0.45-0.47)**	**1.56 (1.54-1.58)**	**8,624**
C16:1	0 to ≤1 day (***a***)	**0.07 (0.06-0.07)**	**0.36 (0.36-0.37)**	**10,346**	**0.07 (0.07-0.07)**	**0.33 (0.33-0.33)**	**9,602**
	1 day to ≤2 days (***b***)	**0.07 (0.07-0.07)**	**0.36 (0.36-0.36)**	**116,328**	**0.07 (0.07-0.07)**	**0.34 (0.34-0.34)**	**105,855**
	2 days to ≤3 days (***c***)	**0.07 (0.07-0.07)**	**0.36 (0.36-0.36)**	**1,057,815**	**0.06 (0.06-0.06)**	**0.33 (0.33-0.33)**	**969,526**
	3 days to ≤4 days (***d***)	**0.06 (0.06-0.06)**	**0.32 (0.32-0.32)**	**804,706**	**0.06 (0.06-0.06)**	**0.30 (0.30-0.30)**	**733,932**
	4 days to ≤5 days (***e***)	**0.05 (0.05-0.05)**	**0.25 (0.25-0.25)**	**159,884**	**0.04 (0.04-0.04)**	**0.22 (0.22-0.22)**	**144,775**
	5 days to ≤6 days (***f***)	**0.04 (0.04-0.04)**	**0.18 (0.18-0.18)**	**97,437**	**0.04 (0.04-0.04)**	**0.17 (0.17-0.17)**	**88,045**
	6 days to ≤7 days (***g***)	**0.04 (0.03-0.04)**	**0.15 (0.15-0.15)**	**60,085**	**0.03 (0.03-0.03)**	**0.14 (0.14-0.14)**	**53,651**
	7 days to ≤8 days (***h***)	**0.03 (0.03-0.03)**	**0.14 (0.14-0.14)**	**34,226**	**0.03 (0.03-0.03)**	**0.12 (0.12-0.12)**	**29,973**
	8 days to ≤9 days (***i***)	**0.03 (0.03-0.03)**	**0.12 (0.12-0.12)**	**25,596**	**0.03 (0.03-0.03)**	**0.11 (0.11-0.11)**	**22,347**
	9 days to ≤10 days (***j***)	**0.03 (0.03-0.03)**	**0.11 (0.11-0.11)**	**20,021**	**0.03 (0.03-0.03)**	**0.10 (0.10-0.10)**	**17,461**
	10 days to ≤11 days (***k***)	**0.03 (0.03-0.03)**	**0.10 (0.10-0.10)**	**15,462**	**0.02 (0.02-0.02)**	**0.09 (0.09-0.09)**	**13,416**
	11 days to ≤12 days (***L***)	**0.02 (0.02-0.02)**	**0.09 (0.09-0.09)**	**14,205**	**0.02 (0.02-0.02)**	**0.08 (0.08-0.08)**	**12,341**
	12 days to ≤13 days (***m***)	**0.02 (0.02-0.02)**	**0.09 (0.09-0.10)**	**12,566**	**0.02 (0.02-0.02)**	**0.08 (0.08-0.08)**	**11,183**
	13 days to ≤14 days (***n***)	**0.02 (0.02-0.02)**	**0.08 (0.08-0.08)**	**11,545**	**0.02 (0.02-0.02)**	**0.07 (0.07-0.07)**	**10,146**
C16:1-OH	0 to ≤1 day (***a***)	**0.02 (0.02-0.02)**	**0.05 (0.05-0.05)**	**9,569**	**0.02 (0.02-0.02)**	**0.05 (0.05-0.05)**	**9,225**
	1 day to ≤2 days (***b***)	**0.02 (0.02-0.02)**	**0.07 (0.07-0.07)**	**115,989**	**0.02 (0.02-0.02)**	**0.05 (0.05-0.05)**	**99,821**
	2 days to ≤3 days (***c***)	**0.02 (0.02-0.02)**	**0.05 (0.05-0.05)**	**980,322**	**0.02 (0.02-0.02)**	**0.05 (0.05-0.05)**	**928,315**
	3 days to ≤4 days (***d***)	**0.02 (0.02-0.02)**	**0.05 (0.05-0.05)**	**754,726**	**0.02 (0.02-0.02)**	**0.05 (0.05-0.05)**	**705,462**
	4 days to ≤5 days (***e***)	**0.02 (0.02-0.02)**	**0.05 (0.05-0.05)**	**149,642**	**0.02 (0.02-0.02)**	**0.06 (0.06-0.06)**	**144,347**
	5 days to ≤6 days (***f***)	**0.02 (0.02-0.02)**	**0.05 (0.05-0.05)**	**90,618**	**0.01 (0.01-0.01)**	**0.06 (0.06-0.06)**	**87,648**
	6 days to ≤7 days (***g***)	**0.01 (0.01-0.01)**	**0.06 (0.06-0.06)**	**59,576**	**0.01 (0.01-0.01)**	**0.05 (0.05-0.06)**	**53,456**
	7 days to ≤8 days (***h***)	**0.01 (0.01-0.01)**	**0.06 (0.06-0.06)**	**33,962**	**0.01 (0.01-0.01)**	**0.04 (0.04-0.04)**	**27,784**
	8 days to ≤9 days (***i***)	**0.01 (0.01-0.01)**	**0.04 (0.04-0.04)**	**23,435**	**0.01 (0.01-0.01)**	**0.04 (0.04-0.04)**	**20,989**
	9 days to ≤10 days (***j***)	**0.01 (0.01-0.01)**	**0.04 (0.04-0.04)**	**18,652**	**0.01 (0.01-0.01)**	**0.04 (0.04–0.04)**	**16,597**
	10 days to ≤11 days (***k***)	**0.01 (0.01-0.01)**	**0.04 (0.04-0.04)**	**14,641**	**0.01 (0.01-0.01)**	**0.04 (0.04-0.04)**	**12,863**
	11 days to ≤12 days (***L***)	**0.01 (0.01-0.01)**	**0.04 (0.04-0.04)**	**13,595**	**0.01 (0.01-0.01)**	**0.04 (0.04-0.04)**	**11,999**
	12 days to ≤13 days (***m***)	**0.01 (0.01-0.01)**	**0.04 (0.04-0.04)**	**12,103**	**0.01 (0.01-0.01)**	**0.04 (0.04-0.04)**	**10,907**
	13 days to ≤14 days (***n***)	**0.01 (0.01-0.01)**	**0.04 (0.04-0.04)**	**11,202**	**0.01 (0.01-0.01)**	**0.04 (0.04-0.04)**	**9,936**
C18	0 to ≤1 day (***a***)	**0.47 (0.46-0.48)**	**1.34 (1.33-1.35)**	**10,309**	**0.47 (0.47-0.48)**	**1.31 (1.30-1.32)**	**9,580**
	1 day to ≤2 days (***b***)	**0.46 (0.46-0.46)**	**1.42 (1.42-1.43)**	**115,826**	**0.45 (0.45-0.45)**	**1.37 (1.37-1.37)**	**105,360**
	2 days to ≤3 days (***c***)	**0.46 (0.46-0.46)**	**1.44 (1.44-1.44)**	**1,052,773**	**0.44 (0.43-0.44)**	**1.40 (1.40-1.40)**	**964,779**
	3 days to ≤4 days (***d***)	**0.43 (0.43-0.43)**	**1.39 (1.39-1.39)**	**801,080**	**0.42 (0.42-0.42)**	**1.34 (1.34-1.34)**	**730,502**
	4 days to ≤5 days (***e***)	**0.37 (0.37-0.37)**	**1.24 (1.24-1.24)**	**158,855**	**0.37 (0.37-0.38)**	**1.21 (1.21-1.22)**	**143,811**
	5 days to ≤6 days (***f***)	**0.34 (0.33-0.34)**	**1.16 (1.16-1.17)**	**96,492**	**0.34 (0.34-0.34)**	**1.12 (1.11-1.12)**	**87,141**
	6 days to ≤7 days (***g***)	**0.32 (0.32-0.32)**	**1.08 (1.08-1.08)**	**59,184**	**0.31 (0.30-0.31)**	**1.06 (1.06-1.07)**	**52,787**
	7 days to ≤8 days (***h***)	**0.30 (0.30-0.30)**	**1.01 (1.01-1.02)**	**33,424**	**0.29 (0.28-0.29)**	**0.98 (0.98-0.99)**	**29,241**
	8 days to ≤9 days (***i***)	**0.28 (0.28-0.28)**	**0.95 (0.95-0.95)**	**24,755**	**0.28 (0.28-0.29)**	**0.94 (0.94-0.95)**	**21,557**
	9 days to ≤10 days (***j***)	**0.27 (0.27-0.27)**	**0.91 (0.91-0.92)**	**19,096**	**0.26 (0.25-0.26)**	**0.91 (0.90-0.91)**	**16,643**
	10 days to ≤11 days (***k***)	0.25 (0.24–0.25)	0.87 (0.86–0.87)	27,104	0.25 (0.24–0.25)	0.87 (0.86–0.87)	27,104
	11 days to ≤12 days (***L***)	0.24 (0.24–0.24)	0.83 (0.82–0.83)	24,437	0.24 (0.24–0.24)	0.83 (0.82–0.83)	24,437
	12 days to ≤13 days (***m***)	0.23 (0.23–0.23)	0.79 (0.79–0.79)	21,305	0.23 (0.23–0.23)	0.79 (0.79–0.79)	21,305
	13 days to ≤14 days (***n***)	0.22 (0.22–0.22)	0.77 (0.77–0.78)	18,900	0.22 (0.22–0.22)	0.77 (0.77–0.78)	18,900
C18:1	0 to ≤1 day (***a***)	**0.71 (0.70-0.73)**	**2.01 (2.00-2.02)**	**10,304**	**0.68 (0.67-0.68)**	**1.90 (1.89-1.92)**	**9,577**
	1 day to ≤2 days (***b***)	**0.79 (0.79-0.79)**	**2.20 (2.20-2.21)**	**115,614**	**0.74 (0.73-0.74)**	**2.06 (2.06-2.07)**	**105,331**
	2 days to ≤3 days (***c***)	**0.83 (0.82-0.83)**	**2.34 (2.34-2.34)**	**1,045,139**	**0.78 (0.78-0.78)**	**2.18 (2.18-2.18)**	**962,034**
	3 days to ≤4 days (***d***)	**0.84 (0.84-0.84)**	**2.35 (2.34-2.35)**	**792,953**	**0.78 (0.78-0.78)**	**2.20 (2.20-2.20)**	**727,544**
	4 days to ≤5 days (***e***)	**0.78 (0.77-0.78)**	**2.26 (2.26-2.26)**	**157,938**	**0.72 (0.71-0.72)**	**2.09 (2.08-2.09)**	**143,612**
	5 days to ≤6 days (***f***)	**0.73 (0.73-0.73)**	**2.17 (2.16-2.17)**	**96,355**	**0.68 (0.67-0.68)**	**2.01 (2.01-2.02)**	**87,431**
	6 days to ≤7 days (***g***)	**0.68 (0.67-0.68)**	**2.05 (2.05-2.06)**	**59,614**	**0.63 (0.62-0.63)**	**1.87 (1.86-1.87)**	**53,262**
	7 days to ≤8 days (***h***)	**0.63 (0.62-0.63)**	**1.94 (1.94-1.95)**	**33,926**	**0.59 (0.59-0.60)**	**1.78 (1.78-1.79)**	**29,696**
	8 days to ≤9 days (***i***)	**0.58 (0.58-0.59)**	**1.81 (1.81-1.82)**	**25,292**	**0.54 (0.54-0.55)**	**1.67 (1.67-1.68)**	**21,961**
	9 days to ≤10 days (***j***)	**0.53 (0.52-0.53)**	**1.72 (1.71-1.73)**	**19,654**	**0.50 (0.50-0.51)**	**1.57 (1.56-1.58)**	**16,968**
	10 days to ≤11 days (***k***)	**0.48 (0.47-0.48)**	**1.57 (1.56-1.58)**	**14,968**	**0.45 (0.44-0.45)**	**1.47 (1.46-1.48)**	**12,776**
	11 days to ≤12 days (***L***)	**0.45 (0.44-0.46)**	**1.49 (1.49-1.50)**	**13,496**	**0.43 (0.42-0.43)**	**1.37 (1.36-1.38)**	**11,475**
	12 days to ≤13 days (***m***)	**0.43 (0.42-0.43)**	**1.38 (1.37-1.39)**	**11,700**	**0.40 (0.39-0.40)**	**1.30 (1.29-1.31)**	**10,078**
	13 days to ≤14 days (***n***)	**0.41 (0.41-0.42)**	**1.31 (1.30-1.32)**	**10,503**	**0.38 (0.38-0.39)**	**1.22 (1.21-1.23)**	**8,831**
C18:2	0 to ≤1 day (***a***)	**0.07 (0.07-0.07)**	**0.36 (0.36-0.36)**	**10,352**	**0.06 (0.05-0.06)**	**0.34 (0.34-0.35)**	**9,600**
	1 day to ≤2 days (***b***)	**0.08 (0.08-0.08)**	**0.42 (0.42-0.42)**	**116,622**	**0.07 (0.07-0.07)**	**0.38 (0.38-0.38)**	**105,882**
	2 days to ≤3 days (***c***)	**0.08 (0.08-0.08)**	**0.43 (0.43-0.43)**	**1,060,123**	**0.07 (0.07-0.07)**	**0.40 (0.40-0.40)**	**969,624**
	3 days to ≤4 days (***d***)	**0.09 (0.09-0.09)**	**0.49 (0.48-0.49)**	**804,682**	**0.08 (0.08-0.08)**	**0.44 (0.44-0.44)**	**733,626**
	4 days to ≤5 days (***e***)	**0.12 (0.12-0.12)**	**0.59 (0.58-0.59)**	**159,056**	**0.10 (0.10-0.10)**	**0.52 (0.52-0.52)**	**144,427**
	5 days to ≤6 days (***f***)	**0.13 (0.12-0.13)**	**0.62 (0.61-0.62)**	**96,614**	**0.12 (0.12-0.12)**	**0.55 (0.54-0.55)**	**87,776**
	6 days to ≤7 days (***g***)	**0.14 (0.14-0.14)**	**0.59 (0.59-0.59)**	**59,706**	**0.12 (0.12-0.12)**	**0.54 (0.54-0.54)**	**53,516**
	7 days to ≤8 days (***h***)	**0.14 (0.13-0.14)**	**0.57 (0.57-0.57)**	**34,021**	**0.12 (0.12-0.12)**	**0.52 (0.52-0.52)**	**29,899**
	8 days to ≤9 days (***i***)	**0.13 (0.13-0.13)**	**0.55 (0.55-0.56)**	**25,492**	**0.12 (0.12-0.13)**	**0.50 (0.50-0.50)**	**22,309**
	9 days to ≤10 days (***j***)	**0.12 (0.11-0.12)**	**0.53 (0.52-0.53)**	**19,955**	**0.11 (0.11-0.11)**	**0.48 (0.47-0.48)**	**17,440**
	10 days to ≤11 days (***k***)	**0.12 (0.12-0.12)**	**0.50 (0.50-0.50)**	**15,427**	**0.11 (0.11-0.11)**	**0.47 (0.47-0.48)**	**13,408**
	11 days to ≤12 days (***L***)	**0.12 (0.12-0.13)**	**0.48 (0.47-0.48)**	**14,181**	**0.11 (0.11-0.12)**	**0.46 (0.46-0.47)**	**12,341**
	12 days to ≤13 days (***m***)	**0.11 (0.10-0.11)**	**0.48 (0.48-0.49)**	**12,559**	**0.10 (0.09-0.10)**	**0.43 (0.43-0.44)**	**11,185**
	13 days to ≤14 days (***m***)	**0.11 (0.10-0.11)**	**0.46 (0.46-0.46)**	**11,542**	**0.10 (0.09-0.10)**	**0.44 (0.44-0.45)**	**10,155**

a For each analyte, the bold and italic letter in the blanket indicates the difference between any two age partitions; same letter or letters shared in common means no significant difference and vice versa.

b Boldfaced partitions indicate sex-specific difference within the age partitions and vice versa. If no statistical difference was found between males and females with the age partitions, data were combined and RIs, re-estimated.

c Abbreviations: ALA, alanine; ARG, arginine; CIT, citrulline; GLY, glycine; LEU, leucine; ILE, isoleucine; ALLO-ILE, alloisoleucine; PRO-OH, hydroxyproline; MET, methionine; ORN, ornithine; PHE, phenylalanine; PRO, proline; TYR, tyrosine; VAL, valine; C0, free carnitine; C2, acetylcarnitine; C3, propionylcarnitine; C3-DC + C4-OH, malonylcarnitine+3-hydroxybutyrylcarnitine; C4, butyrylcarnitine + isobutyrylcarnitine; C4-DC + C5-OH, methylmalonylcarnitine+3-hydroxyisovalerylcarnitine; C5, isovalerylcarnitine + methylbutyrylcarnitine; C5-DC + C6-OH, glutarylcarnitine+3-hydroxyhexanoylcarnitine; C6, hexanoylcarnitine; C6-DC, methylglutarylcarnitine; C8, octanoylcarnitine; C8:1, octenoylcarnitine; C10, decanoylcarnitine; C10:1, decenoylcarnitine; C12, dodecanoylcarnitine; C12:1, dodecenoylcarnitine; C14, tetradecanoylcarnitine; C14:1, tetradecenoylcarnitine; C16, palmitoylcarnitine; C16:1, palmitoleylcarnitine; C16:1-OH, 3-hydroxypalmitoleylcarnitine; C18, stearoylcarnitine; C18:1, oleoylcarnitine; C18:2, linoleoylcarnitine.

**FIGURE 3:**
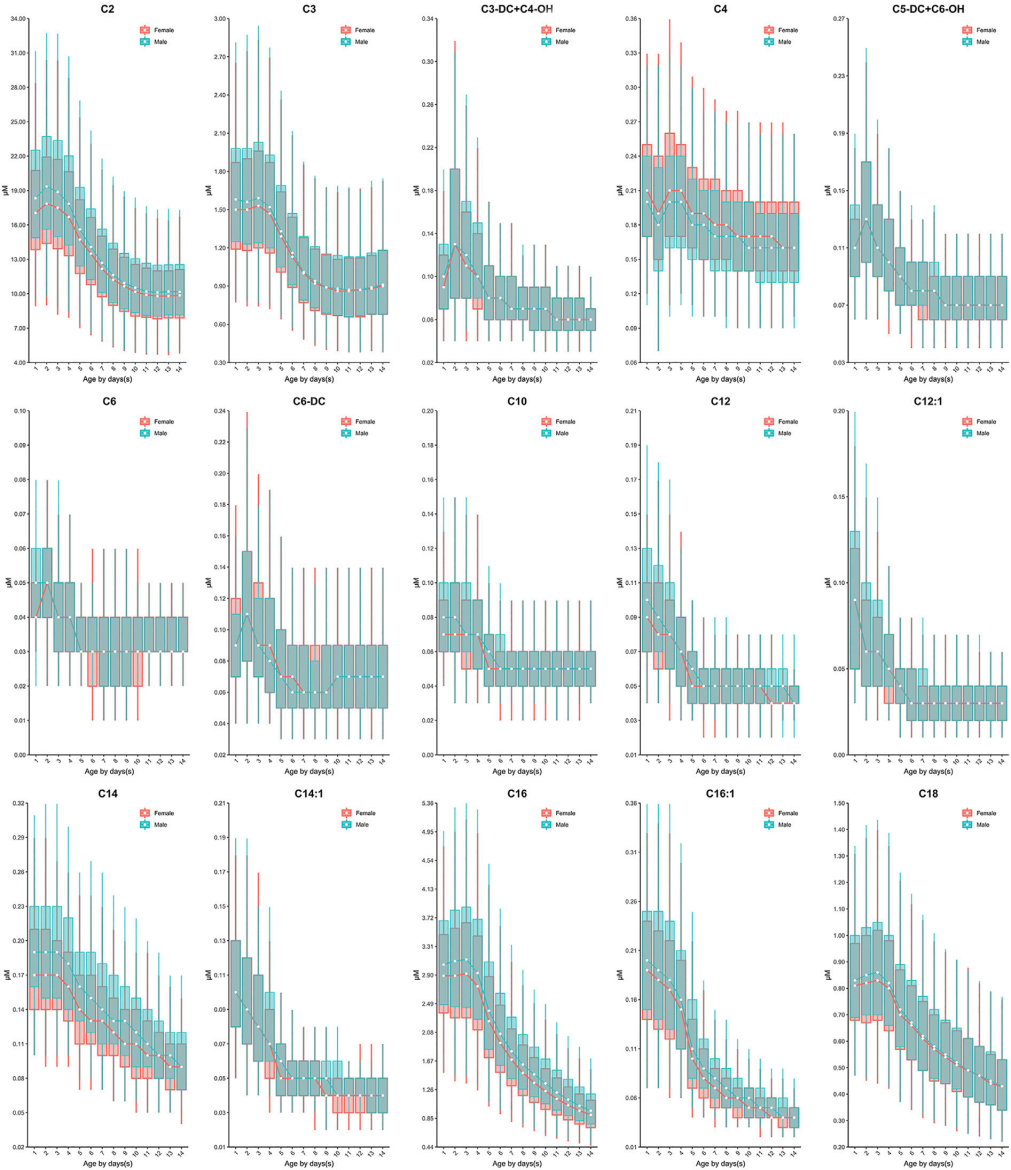
The dynamic change of C2, C3, C3-DC + C4-OH, C4, C5-DC + C6-OH, C6, C6-DC, C10, C12, C12:1, C14, C14:1, C16, C16:1 and C18 over age and sex. The data of male and female partitions are shown in green and red boxes with whiskers, respectively. The boxes extend from the 25^th^ to the 75^th^ percentile, with whiskers extending to the 2.5^th^ or 97.5^th^ percentile. The medians are shown as white circles in the body of the boxes, and are linked with green (male) or red (female) line to shown the dynamic trends over age. Abbreviations are listed in the legend of Table 2.

**FIGURE 4:**
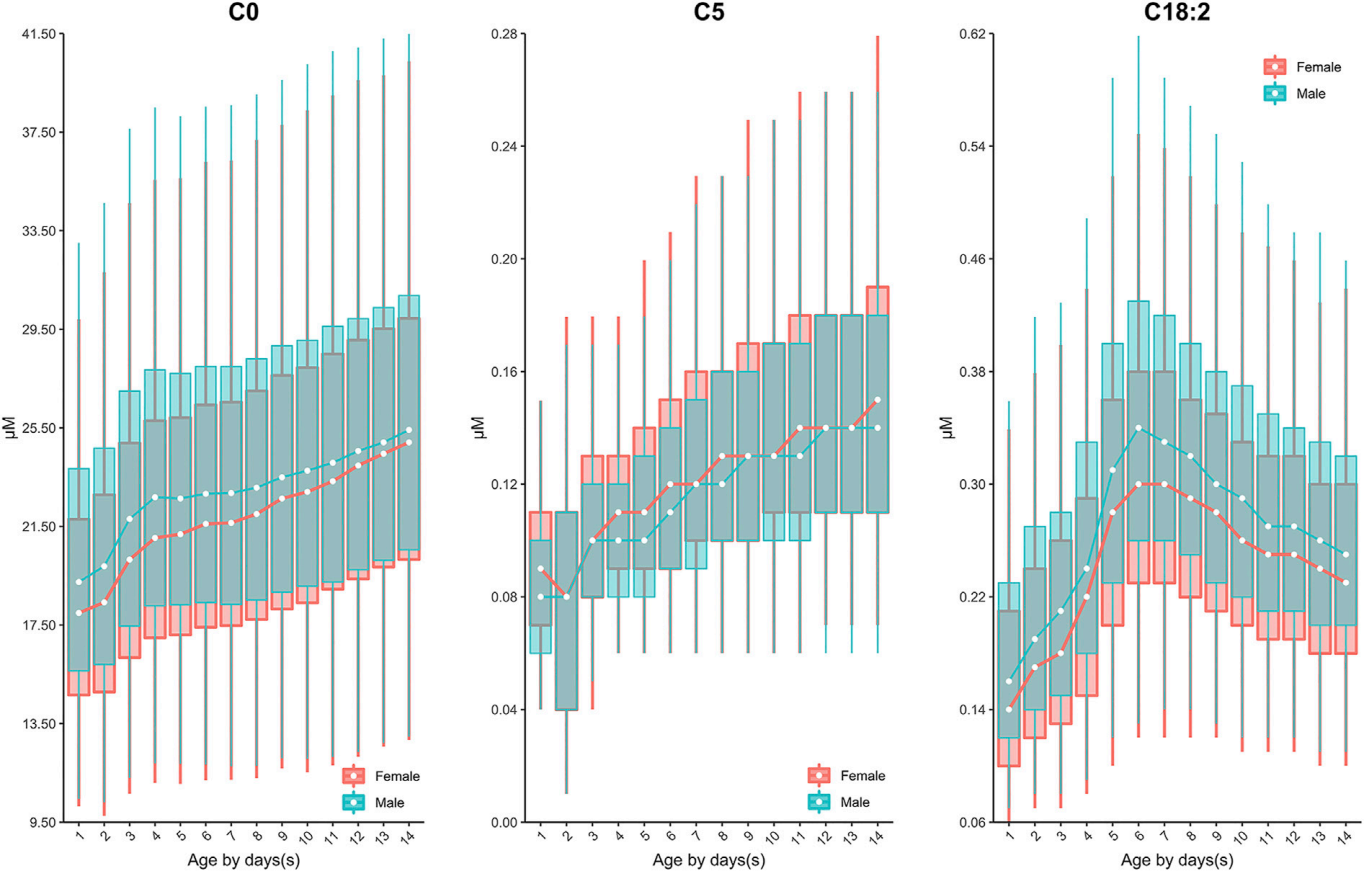
The dynamic change of C0, C5, C18:2 over age and sex. The data of male and female partitions are shown in green and red boxes with whiskers, respectively. The boxes extend from the 25^th^ to the 75^th^ percentile, with whiskers extending to the 2.5^th^ or 97.5^th^ percentile. The medians are shown as white circles in the body of the boxes, and are linked with green (male) or red (female) line to shown the dynamic trends over age. Abbreviations are listed in the legend of Table 2.

Similar to the dynamics of amino acids, the evident fluctuation was observed for most acylcarnitines in the first week of life, followed by relatively constant values from 7 to 14 days. Specifically, the levels of acetylcarnitine (C2), propionylcarnitine (C3), malonylcarnitine+3-hydroxybutyrylcarnitine (C3-DC + C4-OH), butyrylcarnitine/isobutyrylcarnitine (C4), glutarylcarnitine+3-hydroxyhexanoylcarnitine (C5-DC + C6-OH), hexanoylcarnitine (C6), methylglutarylcarnitine (C6-DC), decanoylcarnitine (C10), dodecanoylcarnitine (C12), dodecenoylcarnitine (C12:1), tetradecanoylcarnitine (C14), tetradecenoylcarnitine (C14:1), palmitoylcarnitine (C16), palmitoleylcarnitine (C16:1) and stearoylcarnitine (C18) presented overall downward trend from birth to 7 days of life (Figure 3). In contrast, free carnitine (C0), sovalerylcarnitine + methylbutyrylcarnitine (C5) and linoleoylcarnitine (C18:2) demonstrated increasing concentrations throughout the first week of life (Figure 4). Males demonstrated a higher 2.5th and 97.5th percentile values when compared to females in most acylcarnitines (Table 2). In addition, the lower limits of most analytes in our RIs were smaller than those proposed by R4S (Table 3). The dynamic trends of methionine and other acylcarnitines are shown in Supplementary Figures S1,S2, respectively.

**TABLE 3:** The RIs of R4S versus the RIs developed in this study.

Analyte	Lower limits of RIs (mean ± SD) (μM)			Upper limits of RIs (mean ± SD) (μM)		
	R4S	This study (male)	This study (female)	R4S	This study (male)	This study (female)
ALA	117 ± 26	179.4 ± 6.8	187.8 ± 8.6	507 ± 112	479.1 ± 32.2	491.9 ± 36.7
ARG	2.3 ± 1.1	1.7 ± 0.2	1.7 ± 0.2	32 ± 12	31.3 ± 5.4	29.5 ± 5.1
CIT	6.0 ± 1.7	7.9 ± 0.4	8.2 ± 0.4	28 ± 6.2	22.2 ± 1.8	22.9 ± 1.9
GLY	185 ± 74	233.3 ± 39.2	242.1 ± 40.8	767 ± 230	587.1 ± 103.9	598.8 ± 105.5
LEU/ILE/ALLO-ILE/PRO-OH	—	94.9 ± 11.8	97.8 ± 12.9	—	258.0 ± 27.5	263.4 ± 29.0
MET	11 ± 3.2	9.5 ± 0.9	9.8 ± 0.8	44 ± 11	31.8 ± 1.6	32.6 ± 1.4
ORN	—	61.5 ± 6.9	62.0 ± 7.1	—	196.7 ± 17.5	195.5 ± 18.2
PHE	33 ± 5.9	31.6 ± 2.8	31.9 ± 2.4	97 ± 15	72.2 ± 3.4	72.7 ± 2.9
PRO	—	117.2 ± 4.0	119.9 ± 5.0	—	298.0 ± 22.2	300.1 ± 24.4
TYR	—	46.9 ± 2.5	49.1 ± 2.5	—	168.3 ± 14.5	174.9 ± 14.5
VAL	—	80.7 ± 8.9	84.6 ± 10.3	—	213.4 ± 19.5	221.6 ± 22.1
C0	11 ± 3.1	11.82 ± 0.74	11.35 ± 0.85	59 ± 15	38.86 ± 2.48	36.67 ± 3.02
C2	10 ± 3.2	6.51 ± 1.86	6.22 ± 1.66	52 ± 8.8	23.44 ± 6.22	22.15 ± 5.51
C3	0.57 ± 0.16	0.54 ± 0.17	0.53 ± 0.16	4.74 ± 0.95	2.13 ± 0.52	2.08 ± 0.48
C3-DC + C4-OH	0.040 ± 0.021	0.04 ± 0.01	0.04 ± 0.01	0.33 ± 0.22	0.16 ± 0.06	0.16 ± 0.06
C4	0.080 ± 0.023	0.09 ± 0.01	0.10 ± 0.01	0.75 ± 0.11	0.29 ± 0.03	0.30 ± 0.03
C4-DC + C5-OH	0.090 ± 0.041	0.10 ± 0.01	0.10 ± 0.01	0.45 ± 0.14	0.29 ± 0.02	0.28 ± 0.01
C5	0.050 ± 0.017	0.05 ± 0.01	0.06 ± 0.02	0.39 ± 0.08	0.21 ± 0.04	0.22 ± 0.04
C5-DC + C6-OH	0.020 ± 0.017	0.05 ± 0.01	0.05 ± 0.01	0.25 ± 0.12	0.15 ± 0.04	0.15 ± 0.04
C6	0.020 ± 0.017	0.02 ± 0.00	0.02 ± 0.01	0.18 ± 0.06	0.06 ± 0.01	0.06 ± 0.01
C6-DC	0.022 ± 0.019	0.03 ± 0.01	0.03 ± 0.01	0.17 ± 0.08	0.16 ± 0.03	0.16 ± 0.03
C8	0.020 ± 0.016	0.02 ± 0.00	0.02 ± 0.00	0.21 ± 0.05	0.08 ± 0.01	0.08 ± 0.01
C8:1	—	0.04 ± 0.01	0.04 ± 0.01	—	0.24 ± 0.02	0.24 ± 0.02
C10	0.022 ± 0.006	0.03 ± 0.00	0.03 ± 0.01	0.26 ± 0.06	0.11 ± 0.03	0.10 ± 0.02
C10:1	0.020 ± 0.015	0.03 ± 0.01	0.02 ± 0.00	0.18 ± 0.05	0.11 ± 0.01	0.11 ± 0.02
C12	0.040 ± 0.016	0.03 ± 0.01	0.03 ± 0.01	0.41 ± 0.15	0.11 ± 0.04	0.10 ± 0.04
C12:1	0.010 ± 0.009	0.01 ± 0.01	0.01 ± 0.01	0.27 ± 0.08	0.10 ± 0.04	0.09 ± 0.04
C14	0.071 ± 0.024	0.07 ± 0.02	0.07 ± 0.02	0.50 ± 0.09	0.25 ± 0.05	0.22 ± 0.05
C14:1	0.030 ± 0.010	0.03 ± 0.01	0.03 ± 0.01	0.37 ± 0.07	0.10 ± 0.05	0.10 ± 0.04
C16	0.80 ± 0.33	0.93 ± 0.39	0.87 ± 0.36	6.0 ± 0.9	3.49 ± 1.40	3.24 ± 1.33
C16:1	—	0.04 ± 0.02	0.04 ± 0.02	—	0.19 ± 0.11	0.18 ± 0.10
C16:1-OH	0.011 ± 0.009	0.01 ± 0.01	0.01 ± 0.00	0.13 ± 0.04	0.05 ± 0.01	0.05 ± 0.01
C18	0.31 ± 0.09	0.33 ± 0.09	0.33 ± 0.09	1.7 ± 0.26	1.09 ± 0.24	1.06 ± 0.23
C18:1	0.49 ± 0.11	0.63 ± 0.15	0.59 ± 0.14	2.5 ± 0.30	1.90 ± 0.36	1.76 ± 0.33
C18:2	0.060 ± 0.020	0.11 ± 0.02	0.10 ± 0.02	0.60 ± 0.020	0.50 ± 0.07	0.46 ± 0.06

Abbreviations are listed in the legend of Table 2.

The performance validation of RIs was accomplished in participating centers/laboratories where the level of target biomarkers in 860 true-positives (containing 15 types of conditions), 1,388 false-positives (621 phenylketonuria, 429 methylmalonic acidemia, 224 isovaleric acidemia, 83 maple syrup urine disease and 31 hyperprolinemia) and 10 false-negatives of citrin deficiency was compared to the established RIs. The data suggested that the target biomarker level fell outside the established RIs in 254 false-positives of phenylketonuria (254/621, 40.9%), 136 false-positives of methylmalonic acidemia (136/429, 31.7%), 81 false-positives of isovaleric acidemia (81/224, 36.1%), 22 false positives of maple syrup urine disease (22/82, 26.8%), 11 false-positives of hyperprolinemia (11/31, 35.5%) and 10 false negatives of citrin deficiency (10/10, 100%). In addition, the level of target biomarkers in all true-positives fell outside the proposed reference ranges (Supplementary Table S12).

## Discussion

Currently, dozens of countries have published their recommendations on sampling age for NBS programs, e.g. the United States (>24 h) (CLSI, 2013), Germany (48–72 h) (Lindner et al., 2011), the United Kingdom (120–192h) (Loeber et al., 2012), Australia (48–72 h) (Wilcken and Wiley, 2008) and the United Arab Emirates (>120 h) (al-Hosani et al., 2003). Less information regarding the rationale for setting such a sampling interval is available. In NNSCP, study centers/laboratories were allowed to determine their own sampling time post birth. Although this is not ideal clinically, it enabled us to explore the dynamic changes of biomarkers across a broader age range and determine an ideal sampling age more reasonably. Based on our data, most analytes fluctuated during the first week of life, particularly during the first 5 days following birth (Figure 2 and Figure 3). Such rapid remarkable changes might indicate a response to the postnatal environment. One of the significant physiological differences between the intrauterine and extrauterine environment is that infants must intake nutrients through their own digestive system. In neonatal pigs, arginine has been observed to be synthesized by the enteric mucosal cells with three- to 4-fold higher concentrations in 0–2 day-old pigs than in 7 day-old pigs (Wu and Knabe, 1995). This is consistent with our findings in human neonates, as we observed increasing arginine concentrations with age (Figure 2). In addition, studies have demonstrated the capacity to convert phenylalanine to tyrosine in neonates (Hogewind-Schoonenboom et al., 2015). In the current investigation, increasing concentrations of tyrosine coincided with decreasing concentrations of phenylalanine in the early neonatal period (0–5 days of age), with the exception of 24–48 h of age (1 to ≤2 days) (Figure 2). This may be a result of potential increased conversion of phenylalanine throughout the neonatal period. Similar to the amino acids, most acylcarnitines presented dynamic reference value distributions during the first week after life (Figure 3). For example, increasing concentrations of free carnitine (C0) were observed throughout the testing neonatal age range (Figure 3), in alignment with previous findings (Campoy et al., 1998). However, most other acylcarnitines were determined to decrease in concentration from 0 to 7 days of age in the present study (Figure 3). This is in contrast to a previous publication which reported significant increases in acylcarnitine concentration in the cord blood of newborns (day 0) relative to elder neonates (day 5) (Meyburg et al., 2001). These varied results can likely be attributed to the type of samples examined, since we assessed only the heel blood samples. Although we cannot fully conclude the mechanism behind the changes of analytes, these data provide important information regarding the sampling age. Given the evident fluctuation of most biomarkers during the first 5 days post-birth, sampling after 5 days (120 h) of age would be ideal physiologically. However, as early diagnosis and management can ease or even reverse the course of some metabolic disorders clinically (Leonard and Morris, 2000; Leonard and Morris, 2006), we recommend the MS/MS NBS laboratories/centers to collect blood samples as soon as possible after 2 days (48 h) of birth, when the changes in biomarker concentrations begin to minimize.

In addition to investigating the optimal sampling age, this study aimed to establish accurate and robust RIs for 35 MS/MS biomarkers in healthy Chinese newborns. Both age and sex have been identified as important covariates affecting the children and adolescents associated reference values in whole blood or serum samples (Rauchenzauner et al., 2007; Colantonio et al., 2012). However, age is commonly the only factor that is taken into consideration when interpreting the MS/MS NBS results. This is likely due to small sample sizes that do not allow for partitioning by both age and sex. For example, reports from Thailand (Uaariyapanichkul et al., 2018) and Colombia (Céspedes et al., 2017) have adopted only age-dependent neonatal RIs. In our study, most analytes demonstrated a significant statistical difference between males and females (Table 2), suggesting that neonatal RIs partitioned solely by age may not be suitable. Therefore, we established age and/or sex specific RIs for all testing biomarkers. The RIs partitioned by each day and sex not only enable us to monitor the continuous change of a given analyte over age, but provided detailed information about the variance between males and females within a specific age group (Table 2 **)**. The level of target biomarkers in 860 true-positives, 1,388 false-positives and 10 false-negatives was compared to the reference ranges with the aim of validating the performance of the RIs we proposed. The resulting data suggested that the level of target biomarkers in all true-positive or false-negative cases fell outside the reference ranges, indicating the high sensitivity of the RIs. In addition, the age and sex-stratified RIs presented improved specificity over the RIs routinely used in participating centers/laboratories, as more false-positive cases were identified. Regardless of the recommendation for ideal sampling age, we provide age- and sex-specific RIs for neonates aged 0–14 days, covering most of the neonatal sampling age.

Although there has been great progress in the field of pediatric RIs in recent years, most pediatric initiatives do not focus on young children, particularly neonates, due to challenges with recruitment. For example, the Canadian Laboratory Initiative on Pediatric Reference Intervals (CALIPER) is a national initiative that has established accurate and robust pediatric RIs for over 170 biomarkers, including serum acylcarntinines and amino acids (Lepage et al., 2006; Teodoro-Morrison et al., 2015; Adeli et al., 2017). However, similar to other pediatric RI initiatives, CALIPER has a limited sample size in the first month of life, restricting the establishment of comprehensive RIs for this critical population. There is some existent literature regarding RIs for amino acids and acylcarnitines in healthy newborns. In 2001, Cavedon and colleagues established RIs for acylcarnitine profiles obtained from DBS samples of approximately 10,000 neonates (Cavedon et al., 2005). Their investigation, however, did not partition RIs by age and/or sex throughout the neonatal age period and thus did not capture dynamic patterns. Similarly, Zytkovicz and colleagues calculated the mean and standard deviation of amino acids in healthy newborns, but the impact of age and sex on analyte levels was not further assessed (Zytkovicz et al., 2001). A more recent study by Dietzen and colleagues also sought to establish RIs for amino acids in the neonatal age range (0–4 days, *n* = 310) (Dietzen et al., 2016). They concluded that there was little correlation between age and amino acid concentration from 0 to 4 days of life (Dietzen et al., 2016). While this is in contrast to our findings, differential results could be due to their limited sample size in comparison to our study of millions of neonates.

Clinically, recalling a patient to collect a second DBS sample for testing is not uncommon. In this case, the original NBS RIs are likely no longer applicable due to the rapid growth and development associated with early life, increasing the potential for false positive or negative results. The established RIs in the present study extend to the first 2 weeks following birth, providing an extremely valuable reference for the recall testing.

It is important to note the limitations of the current study. Due to the robustness of our study, it was barely possible to follow subjects for greater than 1 year post-birth. As patients could present with a delayed onset of disorder after 1 year of birth, it is likely that their inclusion in our study could impair the accuracy of biomarker-associated RIs. However, as our study tested more than four million neonatal DBS samples, such error can be largely ignored. The RIs that we proposed might be unsuitable for the low- or high birth weight neonates because the level of amino acids in those newborns was varied from the normal weight babies (Yang et al., 2018). Furthermore, while our study reports statistically significant age- and sex-specific differences in NBS biomarker concentration, some differences could be a result of large sample size and not of clinically relevant. Other limitations are that we did not address whether additional cofactors would affect the profiles of MS/MS NBS biomarker, such as ethnicity, climate, and season change. Because the submitted data from participating centers/centers either not include more detailed information or provide only limited information which would not allow us to make a broad conclusion on the impact of other cofactors. Also, the blood spot samples cannot replace the plasma or serum specimens to confirm the diagnosis of inborn errors. The concentration of biomarkers outside of the RIs does not necessarily indicate the presence of disease.

## Conclusion

In conclusion, this study established age- and sex-stratified RIs for 35 MS/MS NBS biomarkers, including 11 amino acids and 24 acylcarnitines. Most importantly, our data provides strong evidence regarding the age- and sex-specific trends in MS/MS NBS biomarkers based on a huge number of observations. Thus, these data valuably contributed to the current literature and report potential physiologically relevant age- and sex-specific trends in analyte concentrations that could be important for evaluating disease in neonatal populations as early as possible.

## Data Availability

The original contributions presented in the study are included in the article/Supplementary Material, further inquiries can be directed to the corresponding authors.

## References

[B1] Adeli K. Higgins V. Trajcevski K. White-Al Habeeb N. (2017). The canadian Laboratory Initiative on Pediatric Reference Intervals: A Caliper white Paper. Crit. Rev. Clin. Lab. Sci. 54, 358–413. 10.1080/10408363.2017.1379945 29017389

[B2] al-Hosani H. Salah M. Saade D. Osman H. al-Zahid J. (2003). United arab emirates National Newborn Screening Programme: An Evaluation 1998-2000. East. Mediterr. Health J. 9, 324–332. 10.26719/2003.9.1-2.123 15751925

[B3] Campoy C. Bayés R. Peinado J. M. Rivero M. López C. Molina-Font J. A. (1998). Evaluation of Carnitine Nutritional Status in Full-Term Newborn Infants. Early Hum. Development 53, S149–S164. 10.1016/s0378-3782(98)00072-3 10102662

[B4] Cavedon C. T. Bourdoux P. Mertens K. Van Thi H. V. Herremans N. de Laet C. (2005). Age-related Variations in Acylcarnitine and Free Carnitine Concentrations Measured by Tandem Mass Spectrometry. Clin. Chem. 51, 745–752. 10.1373/clinchem.2004.043646 15708951

[B5] Céspedes N. Valencia A. Echeverry C. A. Arce-Plata M. I. Colón C. Castiñeiras D. E. (2017). Reference Values of Amino Acids, Acylcarnitines and Succinylacetone by Tandem Mass Spectrometry for Use in Newborn Screening in Southwest colombia. cm v48, 113–119. 10.25100/cm.v48i3.2180 PMC5687862 29213153

[B6] CLSI (2013). Blood Collection on Filter Paper for Newborn Screening Programs. 6th ed.. Wayne, Pennsylvania: Clinical and Laboratory Standards Institute.

[B7] CLSI (2010). Defining, Establishing, and Verifying Reference Intervals in the Clinical Laboratory. 3rd ed.. Wayne, Pennsylvania: Clinical and Laboratory Standards Institute.

[B8] CLSI (2017). Newborn Screening by Tandem Mass Spectrometry. 2nd ed.. Wayne, Pennsylvania: Clinical and Laboratory Standards Institute.

[B9] Colantonio D. A. Kyriakopoulou L. Chan M. K. Daly C. H. Brinc D. Venner A. A. (2012). Closing the Gaps in Pediatric Laboratory Reference Intervals: A Caliper Database of 40 Biochemical Markers in a Healthy and Multiethnic Population of Children. Clin. Chem. 58, 854–868. 10.1373/clinchem.2011.177741 22371482

[B10] Dietzen D. J. Bennett M. J. Lo S. F. Grey V. L. Jones P. M. (2016). Dried Blood Spot Reference Intervals for Steroids and Amino Acids in a Neonatal Cohort of the National Children's Study. Clin. Chem. 62, 1658–1667. 10.1373/clinchem.2016.263434 27784706

[B11] FDA (2010). “510(k) Substantial Equivalence Determination Decision Summary Assay and Instrument Combination Template,” in NeoBase Non-derivatized MSMS Kit (K093916) (FDA). Available at: https://www.accessdata.fda.gov/cdrh_docs/reviews/K093916.pdf .

[B12] Hogewind-Schoonenboom J. E. Zhu L. Zhu L. Ackermans E. C. Mulders R. Te Boekhorst B. (2015). Phenylalanine Requirements of Enterally Fed Term and Preterm Neonates. Am. J. Clin. Nutr. 101, 1155–1162. 10.3945/ajcn.114.089664 25926506

[B13] Leonard J. Morris A. (2006). Diagnosis and Early Management of Inborn Errors of Metabolism Presenting Around the Time of Birth1. Acta Paediatr. 95, 6–14. 10.1080/08035250500349413 16373289

[B14] Leonard J. Morris A. (2000). Inborn Errors of Metabolism Around Time of Birth. The Lancet 356, 583–587. 10.1016/s0140-6736(00)02591-5 10950248

[B15] Lepage N. Li D. Kavsak P. A. Bamforth F. Callahan J. Dooley K. (2006). Incomplete Pediatric Reference Intervals for the Management of Patients with Inborn Errors of Metabolism. Clin. Biochem. 39, 595–599. 10.1016/j.clinbiochem.2006.02.011 16595129

[B16] Lindner M. Gramer G. Haege G. Fang-Hoffmann J. Schwab K. O. Tacke U. (2011). Efficacy and Outcome of Expanded Newborn Screening for Metabolic Diseases - Report of 10 Years from South-West Germany *. Orphanet J. Rare Dis. 6, 44. 10.1186/1750-1172-6-44 21689452 PMC3141366

[B17] Loeber J. G. Burgard P. Cornel M. C. Rigter T. Weinreich S. S. Rupp K. (2012). Newborn Screening Programmes in Europe; Arguments and Efforts Regarding Harmonization. Part 1 - from Blood Spot to Screening Result. J. Inherit. Metab. Dis. 35, 603–611. 10.1007/s10545-012-9483-0 22552820

[B18] McHugh D. Cameron C. A. Abdenur J. E. Abdulrahman M. Adair O. Al Nuaimi S. A. (2011). Clinical Validation of Cutoff Target Ranges in Newborn Screening of Metabolic Disorders by Tandem Mass Spectrometry: A Worldwide Collaborative Project. Genet. Med. 13, 230–254. 10.1097/GIM.0b013e31820d5e67 21325949

[B19] Meyburg J. Schulze A. Kohlmueller D. Linderkamp O. Mayatepek E. (2001). Postnatal Changes in Neonatal Acylcarnitine Profile. Pediatr. Res. 49, 125–129. 10.1203/00006450-200101000-00024 11134502

[B20] Ogunkeye O. O. Roluga A. I. Khan F. A. (2007). Resetting the Detection Level of Cord Blood Thyroid Stimulating Hormone (Tsh) for the Diagnosis of Congenital Hypothyroidism. J. Trop. Pediatr. 54, 74–77. 10.1093/tropej/fmm082 17878179

[B21] Rauchenzauner M. Schmid A. Heinz-Erian P. Kapelari K. Falkensammer G. Griesmacher A. (2007). Sex- and Age-specific Reference Curves for Serum Markers of Bone Turnover in Healthy Children from 2 Months to 18 Years. J. Clin. Endocrinol. Metab. 92, 443–449. 10.1210/jc.2006-1706 17105843

[B22] Storey J. D. (2003). The Positive False Discovery Rate: A Bayesian Interpretation and the Q-Value. Ann. Stat. 31, 2013–2035. 10.1214/aos/1074290335

[B23] Teodoro-Morrison T. Kyriakopoulou L. Chen Y. K. Raizman J. E. Bevilacqua V. Chan M. K. (2015). Dynamic Biological Changes in Metabolic Disease Biomarkers in Childhood and Adolescence: A Caliper Study of Healthy Community Children. Clin. Biochem. 48, 828–836. 10.1016/j.clinbiochem.2015.05.005 25977068

[B24] Uaariyapanichkul J. Chomtho S. Suphapeetiporn K. Shotelersuk V. Punnahitananda S. Chinjarernpan P. (2018). Age-related Reference Intervals for Blood Amino Acids in Thai Pediatric Population Measured by Liquid Chromatography Tandem Mass Spectrometry. J. Nutr. Metab. 2018, 5124035. 10.1155/2018/5124035 29854440 PMC5960525

[B25] Wilcken B. Wiley V. Hammond J. Carpenter K. (2003). Screening Newborns for Inborn Errors of Metabolism by Tandem Mass Spectrometry. N. Engl. J. Med. 348, 2304–2312. 10.1056/nejmoa025225 12788994

[B26] Wilcken B. Wiley V. (2008). Newborn Screening. Pathology 40, 104–115. 10.1080/00313020701813743 18203033

[B27] Wu G. Knabe D. A. (1995). Arginine Synthesis in Enterocytes of Neonatal Pigs. Am. J. Physiology-Regulatory, Integr. Comp. Physiol. 269, R621–R629. 10.1152/ajpregu.1995.269.3.r621 7573565

[B28] Yang L. Zhang Y. Yang J. Huang X. (2018). Effects of Birth Weight on Profiles of Dried Blood Amino-Acids and Acylcarnitines. Ann. Clin. Biochem. 55, 92–99. 10.1177/0004563216688038 29064274

[B29] Zhan J.-Y. Qin Y.-F. Zhao Z.-Y. (2009). Neonatal Screening for Congenital Hypothyroidism and Phenylketonuria in china. World J. Pediatr. 5, 136–139. 10.1007/s12519-009-0027-0 19718537

[B30] Zhenzhen H. Yang J. Shang S. Huang X. Zhang Y. Lingwei H. (2018). Preliminary Application of Region 4 Stork Project Used in Newborn Screening by Tandem Mass Spectrometry. Chin. J Lab Med 41, 300–304.

[B31] Zytkovicz T. H. Fitzgerald E. F. Marsden D. Larson C. A. Shih V. E. Johnson D. M. (2001). Tandem Mass Spectrometric Analysis for Amino, Organic, and Fatty Acid Disorders in Newborn Dried Blood Spots. Clin. Chem. 47, 1945–1955. 10.1093/clinchem/47.11.1945 11673361

