# Supplementary material for: Reference Standards for Newborn Screening of Metabolic Disorders by Tandem Mass Spectrometry: A Nationwide Study on Millions of Chinese Neonatal Populations
Source: Front Mol Biosci. 2021 Dec 16;8:719866. doi: 10.3389/fmolb.2021.719866 (PMC8716770; doi:10.3389/fmolb.2021.719866)
Supplement: Supplementary file 1 [file DataSheet1.PDF]

Table S1. The MRM parameters of each mass spectrometer

Analytes	Instrument model	Ionization mode	Q1	Q3	Dwell time (s)	Declustering potential	Collision energy
GLY	AB Sciex QTRAP 3200	positive	76.2	30.1	0.05	14	17
	AB Sciex QTRAP 4000	positive	76	30.1	0.025	40	19
	AB Sciex QTRAP 4500	positive	76	30	0.025	30	16
	Waters Quattro micro AP	positive	76.1	30	0.1	16	8
	Waters Acquity TQD	positive	76	30	0.05	18	7
	Waters Xevo TQD	positive	76	30.1	0.025	20	7
GLY IS	AB Sciex QTRAP 3200	positive	78.2	32.1	0.05	14	17
	AB Sciex QTRAP 4000	positive	78	32.1	0.025	40	19
	AB Sciex QTRAP 4500	positive	78	32	0.025	30	16
	Waters Quattro micro AP	positive	78.1	32	0.1	16	8
	Waters Acquity TQD	positive	78	32	0.05	18	7
	Waters Xevo TQD	positive	78	32.1	0.025	20	7
ALA	AB Sciex QTRAP 3200	positive	90.2	44.1	0.025	18	22
	AB Sciex QTRAP 4000	positive	90	44.1	0.025	45	16
	AB Sciex QTRAP 4500	positive	90	44	0.025	31	14
	Waters Quattro micro AP	positive	90.1	44	0.1	17	8
	Waters Acquity TQD	positive	90	44	0.025	19	7
	Waters Xevo TQD	positive	90	44.1	0.025	22	8
ALA IS	AB Sciex QTRAP 3200	positive	94.2	48.1	0.025	18	22
	AB Sciex QTRAP 4000	positive	94	48.1	0.025	45	16
	AB Sciex QTRAP 4500	positive	94	48	0.025	31	14
	Waters Quattro micro AP	positive	94.1	48	0.1	17	8
	Waters Acquity TQD	positive	94	48	0.025	19	7
	Waters Xevo TQD	positive	94	48.1	0.025	22	8
PRO	AB Sciex QTRAP 3200	positive	116.1	70.1	0.025	12	21
	AB Sciex QTRAP 4000	positive	116.1	70.1	0.025	26	22
	AB Sciex QTRAP 4500	positive	116.1	70.1	0.025	12	21
	Waters Quattro micro AP	positive	116.1	70	0.05	10	13
	Waters Acquity TQD	positive	116.1	70.1	0.025	12	14
	Waters Xevo TQD	positive	116.1	70.1	0.025	14	14
VAL	AB Sciex QTRAP 3200	positive	118.1	72.1	0.025	22	18
	AB Sciex QTRAP 4000	positive	118.1	72.1	0.025	49	17
	AB Sciex QTRAP 4500	positive	118.1	72.1	0.025	25	15
	Waters Quattro micro AP	positive	118.1	72	0.05	18	10
	Waters Acquity TQD	positive	118.1	72.1	0.025	22	10
	Waters Xevo TQD	positive	118.1	72.1	0.025	25	10
PRO IS	AB Sciex QTRAP 3200	positive	121.1	74.1	0.025	12	21
	AB Sciex QTRAP 4000	positive	121.1	74.1	0.025	26	22
	AB Sciex QTRAP 4500	positive	121.1	74.1	0.025	12	21
	Waters Quattro micro AP	positive	121.1	74	0.05	10	13
	Waters Acquity TQD	positive	121.1	74.1	0.025	12	14
	Waters Xevo TQD	positive	121.1	74.1	0.025	14	14
VAL IS	AB Sciex QTRAP 3200	positive	126.2	80.1	0.025	22	18
	AB Sciex QTRAP 4000	positive	126.1	80.1	0.025	49	17
	AB Sciex QTRAP 4500	positive	126.1	80.1	0.025	25	15
	Waters Quattro micro AP	positive	126.1	80	0.05	18	10
	Waters Acquity TQD	positive	126.1	80.1	0.025	22	10
	Waters Xevo TQD	positive	126.1	80.1	0.025	25	10
LEU/ILE/ALLO-ILE/PRO-OH	AB Sciex QTRAP 3200	positive	132.2	86.1	0.025	23	13
	AB Sciex QTRAP 4000	positive	132.1	86.1	0.025	52	15
	AB Sciex QTRAP 4500	positive	132.1	86.1	0.025	28	13
	Waters Quattro micro AP	positive	132.1	86	0.05	16	10
	Waters Acquity TQD	positive	132.1	86.1	0.025	22	10

	Waters Xevo TQD	positive	132.1	86.1	0.025	24	10
ORN	AB Sciex QTRAP 3200	positive	133.2	70.1	0.025	24	27
	AB Sciex QTRAP 4000	positive	133.1	70.1	0.025	43	24
	AB Sciex QTRAP 4500	positive	133.1	70.1	0.025	26	24
	Waters Quattro micro AP	positive	133.1	70	0.05	15	16
	Waters Acquity TQD	positive	133.1	70.1	0.025	21	15
	Waters Xevo TQD	positive	133.1	70.1	0.025	22	15
LEU/ILE/ALLO-ILE/PRO-OH IS	AB Sciex QTRAP 3200	positive	135.2	89.1	0.025	23	13
	AB Sciex QTRAP 4000	positive	135.1	89.1	0.025	52	15
	AB Sciex QTRAP 4500	positive	135.1	89.1	0.025	28	13
	Waters Quattro micro AP	positive	135.1	89	0.05	16	10
	Waters Acquity TQD	positive	135.1	89.1	0.025	22	10
	Waters Xevo TQD	positive	135.1	89.1	0.025	24	10
ORN IS	AB Sciex QTRAP 3200	positive	139.2	76.1	0.025	24	27
	AB Sciex QTRAP 4000	positive	139.1	76.1	0.025	43	24
	AB Sciex QTRAP 4500	positive	139.1	76.1	0.025	26	24
	Waters Quattro micro AP	positive	139.1	76	0.05	15	16
	Waters Acquity TQD	positive	139.1	76.1	0.025	21	15
	Waters Xevo TQD	positive	139.1	76.1	0.025	22	15
MET	AB Sciex QTRAP 3200	positive	150.2	104.1	0.025	23	16
	AB Sciex QTRAP 4000	positive	150.1	104.1	0.025	48	15
	AB Sciex QTRAP 4500	positive	150.1	104.1	0.025	27	14
	Waters Quattro micro AP	positive	150.1	104.1	0.05	16	10
	Waters Acquity TQD	positive	150.1	104.1	0.025	20	10
	Waters Xevo TQD	positive	150.1	104.1	0.025	25	10
MET IS	AB Sciex QTRAP 3200	positive	153.2	107.1	0.025	23	16
	AB Sciex QTRAP 4000	positive	153.1	107.1	0.025	48	15
	AB Sciex QTRAP 4500	positive	153.1	107.1	0.025	27	14
	Waters Quattro micro AP	positive	153.1	107.1	0.05	16	10
	Waters Acquity TQD	positive	153.1	107.1	0.025	20	10
	Waters Xevo TQD	positive	153.1	107.1	0.025	25	10
PHE	AB Sciex QTRAP 3200	positive	166.2	120.1	0.025	27	20
	AB Sciex QTRAP 4000	positive	166.1	120.1	0.025	53	18
	AB Sciex QTRAP 4500	positive	166.1	120.1	0.025	30	18
	Waters Quattro micro AP	positive	166.1	120.1	0.05	16	12
	Waters Acquity TQD	positive	166.1	120.1	0.025	23	12
	Waters Xevo TQD	positive	166.1	120.1	0.025	22	13
PHE IS	AB Sciex QTRAP 3200	positive	172.2	126.1	0.025	27	20
	AB Sciex QTRAP 4000	positive	172.1	126.1	0.025	53	18
	AB Sciex QTRAP 4500	positive	172.1	126.1	0.025	30	18
	Waters Quattro micro AP	positive	172.1	126.1	0.05	16	12
	Waters Acquity TQD	positive	172.1	126.1	0.025	23	12
	Waters Xevo TQD	positive	172.1	126.1	0.025	22	13
ARG	AB Sciex QTRAP 3200	positive	175.2	70.1	0.025	33	33
	AB Sciex QTRAP 4000	positive	175.1	70.1	0.025	58	33
	AB Sciex QTRAP 4500	positive	175.1	70.1	0.025	30	31
	Waters Quattro micro AP	positive	175.1	70.1	0.05	20	20
	Waters Acquity TQD	positive	175.1	70.1	0.025	26	20
	Waters Xevo TQD	positive	175.1	70.1	0.025	32	23
CIT	AB Sciex QTRAP 3200	positive	176.2	113.1	0.025	21	21
	AB Sciex QTRAP 4000	positive	176.1	113.1	0.025	48	23
	AB Sciex QTRAP 4500	positive	176.1	113.1	0.025	25	22
	Waters Quattro micro AP	positive	176.1	113.1	0.05	15	16
	Waters Acquity TQD	positive	176.1	113.1	0.025	18	16
	Waters Xevo TQD	positive	176.1	113.1	0.025	24	15
CIT IS	AB Sciex QTRAP 3200	positive	178.2	115.1	0.025	21	21
	AB Sciex QTRAP 4000	positive	178.1	115.1	0.025	48	23

	AB Sciex QTRAP 4500	positive	178.1	115.1	0.025	25	22
	Waters Quattro micro AP	positive	178.1	115.1	0.05	15	16
	Waters Acquity TQD	positive	178.1	115.1	0.025	18	16
	Waters Xevo TQD	positive	178.1	115.1	0.025	24	15
ARG IS	AB Sciex QTRAP 3200	positive	180.2	75.1	0.025	33	33
	AB Sciex QTRAP 4000	positive	180.1	75.1	0.025	58	33
	AB Sciex QTRAP 4500	positive	180.1	75.1	0.025	30	31
	Waters Quattro micro AP	positive	180.1	75.1	0.05	20	20
	Waters Acquity TQD	positive	180.1	75.1	0.025	26	20
	Waters Xevo TQD	positive	180.1	75.1	0.025	32	23
TYR	AB Sciex QTRAP 3200	positive	182.2	136.1	0.025	28	16
	AB Sciex QTRAP 4000	positive	182.1	136.1	0.025	52	19
	AB Sciex QTRAP 4500	positive	182.1	136.1	0.025	30	19
	Waters Quattro micro AP	positive	182.1	136.1	0.05	16	13
	Waters Acquity TQD	positive	182.1	136.1	0.025	22	14
	Waters Xevo TQD	positive	182.1	136.1	0.025	25	14
TYR IS	AB Sciex QTRAP 3200	positive	188.2	142.1	0.025	28	16
	AB Sciex QTRAP 4000	positive	188.1	142.1	0.025	52	19
	AB Sciex QTRAP 4500	positive	188.1	142.1	0.025	30	19
	Waters Quattro micro AP	positive	188.1	142.1	0.05	16	13
	Waters Acquity TQD	positive	188.1	142.1	0.025	22	14
	Waters Xevo TQD	positive	188.1	142.1	0.025	25	14
C0	AB Sciex QTRAP 3200	positive	162.2	103.1	0.025	36	24
	AB Sciex QTRAP 4000	positive	162.1	103.1	0.025	62	24
	AB Sciex QTRAP 4500	positive	162.1	103.1	0.025	45	22
	Waters Quattro micro AP	positive	162.1	103.1	0.05	26	16
	Waters Acquity TQD	positive	162.15	103.1	0.025	32	16
	Waters Xevo TQD	positive	162.1	103.1	0.025	36	16
C0 IS	AB Sciex QTRAP 3200	positive	171.3	103.1	0.025	36	24
	AB Sciex QTRAP 4000	positive	171.1	103.1	0.025	62	24
	AB Sciex QTRAP 4500	positive	171.1	103.1	0.025	45	22
	Waters Quattro micro AP	positive	171.1	103.1	0.05	26	16
	Waters Acquity TQD	positive	171.15	103.1	0.025	32	16
	Waters Xevo TQD	positive	171.1	103.1	0.025	36	16
C2	AB Sciex QTRAP 3200	positive	204.3	85	0.025	30	26
	AB Sciex QTRAP 4000	positive	204.2	85	0.025	55	26
	AB Sciex QTRAP 4500	positive	204.1	85	0.025	35	23
	Waters Quattro micro AP	positive	204.1	85	0.05	23	17
	Waters Acquity TQD	positive	204.1	85	0.025	28	17
	Waters Xevo TQD	positive	204.1	85	0.025	35	18
C2 IS	AB Sciex QTRAP 3200	positive	207.3	85	0.025	30	26
	AB Sciex QTRAP 4000	positive	207.2	85	0.025	55	26
	AB Sciex QTRAP 4500	positive	207.1	85	0.025	35	23
	Waters Quattro micro AP	positive	207.1	85	0.05	23	17
	Waters Acquity TQD	positive	207.1	85	0.025	28	17
	Waters Xevo TQD	positive	207.1	85	0.025	35	18
C3	AB Sciex QTRAP 3200	positive	218.3	85	0.025	33	26
	AB Sciex QTRAP 4000	positive	218.2	85	0.025	58	27
	AB Sciex QTRAP 4500	positive	218.1	85	0.025	35	24
	Waters Quattro micro AP	positive	218.1	85	0.05	23	18
	Waters Acquity TQD	positive	218.1	85	0.025	29	19
	Waters Xevo TQD	positive	218.1	85	0.025	35	18
C3 IS	AB Sciex QTRAP 3200	positive	221.3	85	0.025	33	26
	AB Sciex QTRAP 4000	positive	221.2	85	0.025	58	27
	AB Sciex QTRAP 4500	positive	221.1	85	0.025	35	24
	Waters Quattro micro AP	positive	221.1	85	0.05	23	18
	Waters Acquity TQD	positive	221.1	85	0.025	29	19

	Waters Xevo TQD	positive	221.1	85	0.025	35	18
C4	AB Sciex QTRAP 3200	positive	232.3	85	0.025	34	28
	AB Sciex QTRAP 4000	positive	232.2	85	0.025	58	28
	AB Sciex QTRAP 4500	positive	232.2	85	0.025	38	24
	Waters Quattro micro AP	positive	232.2	85	0.05	24	19
	Waters Acquity TQD	positive	232.1	85	0.025	29	20
	Waters Xevo TQD	positive	232.1	85	0.025	35	20
C4 IS	AB Sciex QTRAP 3200	positive	235.3	85	0.025	34	28
	AB Sciex QTRAP 4000	positive	235.2	85	0.025	58	28
	AB Sciex QTRAP 4500	positive	235.2	85	0.025	38	24
	Waters Quattro micro AP	positive	235.2	85	0.05	24	19
	Waters Acquity TQD	positive	235.1	85	0.025	29	20
	Waters Xevo TQD	positive	235.1	85	0.025	35	20
C5	AB Sciex QTRAP 3200	positive	246.3	85	0.025	35	33
	AB Sciex QTRAP 4000	positive	246.2	85	0.025	62	30
	AB Sciex QTRAP 4500	positive	246.2	85	0.025	38	24
	Waters Quattro micro AP	positive	246.2	85	0.05	25	20
	Waters Acquity TQD	positive	246.2	85	0.025	30	21
	Waters Xevo TQD	positive	246.1	85	0.025	36	22
C3-DC+C4-OH	AB Sciex QTRAP 3200	positive	248.1	85	0.025	34	28
	AB Sciex QTRAP 4000	positive	248.2	85	0.025	58	28
	AB Sciex QTRAP 4500	positive	248.1	85	0.025	38	24
	Waters Quattro micro AP	positive	248.2	85	0.05	24	19
	Waters Acquity TQD	positive	248.2	85	0.025	29	20
	Waters Xevo TQD	positive	248.1	85	0.025	35	20
C5 IS	AB Sciex QTRAP 3200	positive	255.3	85	0.025	35	33
	AB Sciex QTRAP 4000	positive	255.2	85	0.025	62	30
	AB Sciex QTRAP 4500	positive	255.2	85	0.025	38	24
	Waters Quattro micro AP	positive	255.2	85	0.05	25	20
	Waters Acquity TQD	positive	255.2	85	0.025	30	21
	Waters Xevo TQD	positive	255.1	85	0.025	36	22
C6	AB Sciex QTRAP 3200	positive	260.3	85	0.025	33	35
	AB Sciex QTRAP 4000	positive	260.2	85	0.025	65	30
	AB Sciex QTRAP 4500	positive	260.2	85	0.025	38	24
	Waters Quattro micro AP	positive	260.2	85	0.05	25	20
	Waters Acquity TQD	positive	260.2	85	0.025	31	21
	Waters Xevo TQD	positive	260.2	85	0.025	38	22
C4-DC+C5-OH	AB Sciex QTRAP 3200	positive	262.3	85	0.025	35	33
	AB Sciex QTRAP 4000	positive	262.2	85	0.025	62	30
	AB Sciex QTRAP 4500	positive	262.2	85	0.025	38	24
	Waters Quattro micro AP	positive	262.2	85	0.05	25	20
	Waters Acquity TQD	positive	262.2	85	0.025	30	21
	Waters Xevo TQD	positive	262.2	85	0.025	36	22
C6 IS	AB Sciex QTRAP 3200	positive	263.3	85	0.025	33	35
	AB Sciex QTRAP 4000	positive	263.2	85	0.025	65	30
	AB Sciex QTRAP 4500	positive	263.2	85	0.025	38	24
	Waters Quattro micro AP	positive	263.2	85	0.05	25	20
	Waters Acquity TQD	positive	263.2	85	0.025	31	21
	Waters Xevo TQD	positive	263.2	85	0.025	38	22
C5-DC+C6-OH	AB Sciex QTRAP 3200	positive	276.3	85	0.05	36	39
	AB Sciex QTRAP 4000	positive	276.1	85	0.025	70	31
	AB Sciex QTRAP 4500	positive	276.1	85	0.025	40	27
	Waters Quattro micro AP	positive	276.2	85	0.05	27	22
	Waters Acquity TQD	positive	276.2	85	0.5	32	22
	Waters Xevo TQD	positive	276.2	85	0.025	38	24
C5-DC IS	AB Sciex QTRAP 3200	positive	282.3	85	0.05	36	39
	AB Sciex QTRAP 4000	positive	282.1	85	0.025	70	31

	AB Sciex QTRAP 4500	positive	282.1	85	0.025	40	27
	Waters Quattro micro AP	positive	282.2	85	0.05	27	22
	Waters Acquity TQD	positive	282.2	85	0.5	32	22
	Waters Xevo TQD	positive	282.2	85	0.025	38	24
C8:1	AB Sciex QTRAP 3200	positive	286.3	85	0.025	40	40
	AB Sciex QTRAP 4000	positive	286.2	85	0.025	70	35
	AB Sciex QTRAP 4500	positive	286.2	85	0.025	40	25
	Waters Quattro micro AP	positive	286.2	85	0.05	28	22
	Waters Acquity TQD	positive	286.2	85	0.025	33	22
	Waters Xevo TQD	positive	286.3	85	0.025	40	24
C8	AB Sciex QTRAP 3200	positive	288.3	85	0.025	40	40
	AB Sciex QTRAP 4000	positive	288.2	85	0.025	70	35
	AB Sciex QTRAP 4500	positive	288.2	85	0.025	40	25
	Waters Quattro micro AP	positive	288.2	85	0.05	28	22
	Waters Acquity TQD	positive	288.2	85	0.025	33	22
	Waters Xevo TQD	positive	288.3	85	0.025	40	24
C6-DC	AB Sciex QTRAP 3200	positive	290.3	85	0.05	36	39
	AB Sciex QTRAP 4000	positive	290.1	85	0.025	70	31
	AB Sciex QTRAP 4500	positive	290.2	85	0.025	40	27
	Waters Quattro micro AP	positive	290.2	85	0.05	27	22
	Waters Acquity TQD	positive	290.2	85	0.5	32	22
	Waters Xevo TQD	positive	290.2	85	0.025	38	24
C8 IS	AB Sciex QTRAP 3200	positive	291.3	85	0.025	40	40
	AB Sciex QTRAP 4000	positive	291.2	85	0.025	70	35
	AB Sciex QTRAP 4500	positive	291.2	85	0.025	40	25
	Waters Quattro micro AP	positive	291.2	85	0.05	28	22
	Waters Acquity TQD	positive	291.2	85	0.025	33	22
	Waters Xevo TQD	positive	291.3	85	0.025	40	24
C10:1	AB Sciex QTRAP 3200	positive	314.3	85	0.025	49	40
	AB Sciex QTRAP 4000	positive	314.2	85	0.025	75	38
	AB Sciex QTRAP 4500	positive	314.2	85	0.025	45	30
	Waters Quattro micro AP	positive	314.3	85	0.05	28	22
	Waters Acquity TQD	positive	314.2	85	0.025	34	24
	Waters Xevo TQD	positive	314.3	85	0.025	40	24
C10	AB Sciex QTRAP 3200	positive	316.3	85	0.025	49	40
	AB Sciex QTRAP 4000	positive	316.2	85	0.025	75	38
	AB Sciex QTRAP 4500	positive	316.2	85	0.025	45	30
	Waters Quattro micro AP	positive	316.3	85	0.05	28	22
	Waters Acquity TQD	positive	316.2	85	0.025	34	24
	Waters Xevo TQD	positive	316.3	85	0.025	40	24
C10 IS	AB Sciex QTRAP 3200	positive	319.3	85	0.025	49	40
	AB Sciex QTRAP 4000	positive	319.2	85	0.025	75	38
	AB Sciex QTRAP 4500	positive	319.2	85	0.025	45	30
	Waters Quattro micro AP	positive	319.3	85	0.05	28	22
	Waters Acquity TQD	positive	319.2	85	0.025	34	24
	Waters Xevo TQD	positive	319.3	85	0.025	40	24
C12:1	AB Sciex QTRAP 3200	positive	342.4	85	0.025	52	46
	AB Sciex QTRAP 4000	positive	342.2	85	0.025	77	43
	AB Sciex QTRAP 4500	positive	342.3	85	0.025	50	40
	Waters Quattro micro AP	positive	342.3	85	0.05	30	24
	Waters Acquity TQD	positive	342.2	85	0.025	36	25
	Waters Xevo TQD	positive	342.3	85	0.025	40	26
C12	AB Sciex QTRAP 3200	positive	344.4	85	0.025	52	46
	AB Sciex QTRAP 4000	positive	344.2	85	0.025	77	43
	AB Sciex QTRAP 4500	positive	344.3	85	0.025	50	40
	Waters Quattro micro AP	positive	344.3	85	0.05	30	24
	Waters Acquity TQD	positive	344.2	85	0.025	36	25

	Waters Xevo TQD	positive	344.3	85	0.025	40	26
C12 IS	AB Sciex QTRAP 3200	positive	347.4	85	0.025	52	46
	AB Sciex QTRAP 4000	positive	347.2	85	0.025	77	43
	AB Sciex QTRAP 4500	positive	347.3	85	0.025	50	40
	Waters Quattro micro AP	positive	347.3	85	0.05	30	24
	Waters Acquity TQD	positive	347.2	85	0.025	36	25
	Waters Xevo TQD	positive	347.3	85	0.025	40	26
C14:1	AB Sciex QTRAP 3200	positive	370.4	85	0.025	55	48
	AB Sciex QTRAP 4000	positive	370.3	85	0.025	80	46
	AB Sciex QTRAP 4500	positive	370.3	85	0.025	50	45
	Waters Quattro micro AP	positive	370.3	85	0.05	33	26
	Waters Acquity TQD	positive	370.3	85	0.025	36	27
	Waters Xevo TQD	positive	370.3	85	0.025	42	26
C14	AB Sciex QTRAP 3200	positive	372.4	85	0.025	55	48
	AB Sciex QTRAP 4000	positive	372.3	85	0.025	80	46
	AB Sciex QTRAP 4500	positive	372.3	85	0.025	50	45
	Waters Quattro micro AP	positive	372.3	85	0.05	33	26
	Waters Acquity TQD	positive	372.3	85	0.025	36	27
	Waters Xevo TQD	positive	372.3	85	0.025	42	26
C14 IS	AB Sciex QTRAP 3200	positive	375.4	85	0.025	55	48
	AB Sciex QTRAP 4000	positive	375.3	85	0.025	80	46
	AB Sciex QTRAP 4500	positive	375.3	85	0.025	50	45
	Waters Quattro micro AP	positive	375.3	85	0.05	33	26
	Waters Acquity TQD	positive	375.3	85	0.025	36	27
	Waters Xevo TQD	positive	375.3	85	0.025	42	26
C16:1	AB Sciex QTRAP 3200	positive	398.4	85	0.025	58	50
	AB Sciex QTRAP 4000	positive	398.3	85	0.025	90	49
	AB Sciex QTRAP 4500	positive	398.3	85	0.025	55	50
	Waters Quattro micro AP	positive	398.4	85	0.05	35	27
	Waters Acquity TQD	positive	398.3	85	0.025	38	27
	Waters Xevo TQD	positive	398.3	85	0.025	44	28
C16	AB Sciex QTRAP 3200	positive	400.4	85	0.025	58	50
	AB Sciex QTRAP 4000	positive	400.3	85	0.025	90	49
	AB Sciex QTRAP 4500	positive	400.3	85	0.025	55	50
	Waters Quattro micro AP	positive	400.4	85	0.05	35	27
	Waters Acquity TQD	positive	400.3	85	0.025	38	27
	Waters Xevo TQD	positive	400.3	85	0.025	44	28
C16 IS	AB Sciex QTRAP 3200	positive	403.4	85	0.025	58	50
	AB Sciex QTRAP 4000	positive	403.3	85	0.025	90	49
	AB Sciex QTRAP 4500	positive	403.3	85	0.025	55	50
	Waters Quattro micro AP	positive	403.4	85	0.05	35	27
	Waters Acquity TQD	positive	403.3	85	0.025	38	27
	Waters Xevo TQD	positive	403.3	85	0.025	44	28
C16:1-OH	AB Sciex QTRAP 3200	positive	414.4	85	0.025	58	50
	AB Sciex QTRAP 4000	positive	414.3	85	0.025	90	49
	AB Sciex QTRAP 4500	positive	414.3	85	0.025	55	50
	Waters Quattro micro AP	positive	414. 4	85	0.05	35	27
	Waters Acquity TQD	positive	414.3	85	0.025	38	27
	Waters Xevo TQD	positive	414.3	85	0.025	44	28
C18:2	AB Sciex QTRAP 3200	positive	424.4	85	0.025	60	50
	AB Sciex QTRAP 4000	positive	424.4	85	0.025	95	52
	AB Sciex QTRAP 4500	positive	424.4	85	0.025	55	55
	Waters Quattro micro AP	positive	424.4	85	0.05	37	28
	Waters Acquity TQD	positive	424.4	85	0.025	38	28
	Waters Xevo TQD	positive	424.4	85	0.025	46	28
C18:1	AB Sciex QTRAP 3200	positive	426.4	85	0.025	60	50
	AB Sciex QTRAP 4000	positive	426.4	85	0.025	95	52

	AB Sciex QTRAP 4500	positive	426.4	85	0.025	55	55
	Waters Quattro micro AP	positive	426.4	85	0.05	37	28
	Waters Acquity TQD	positive	426.4	85	0.025	38	28
	Waters Xevo TQD	positive	426.4	85	0.025	46	28
C18	AB Sciex QTRAP 3200	positive	428.4	85	0.025	60	50
	AB Sciex QTRAP 4000	positive	428.4	85	0.025	95	52
	AB Sciex QTRAP 4500	positive	428.4	85	0.025	55	55
	Waters Quattro micro AP	positive	428.4	85	0.05	37	28
	Waters Acquity TQD	positive	428.4	85	0.025	38	28
	Waters Xevo TQD	positive	428.4	85	0.025	46	28
C18 IS	AB Sciex QTRAP 3200	positive	431.4	85	0.025	60	50
	AB Sciex QTRAP 4000	positive	431.4	85	0.025	95	52
	AB Sciex QTRAP 4500	positive	431.4	85	0.025	55	55
	Waters Quattro micro AP	positive	431.4	85	0.05	37	28
	Waters Acquity TQD	positive	431.4	85	0.025	38	28
	Waters Xevo TQD	positive	431.4	85	0.025	46	28

Abbreviations: IS, internal standard; ALA, alanine; ARG, arginine; CIT, citrulline; GLY, glycine; LEU, leucine; ILE, isoleucine; ALLO-ILE, alloisoleucine; PRO-OH, hydroxyproline; MET, methionine; ORN, ornithine; PHE, phenylalanine; PRO, proline; TYR, Tyrosine; VAL, valine; C0, free carnitine; C2, acetylcarnitine; C3, propionylcarnitine; C3-DC+C4-OH, malonylcarnitine+3-hydroxybutyrylcarnitine; C4, butyrylcarnitine+isobutyrylcarnitine; C4-DC+C5-OH, methylmalonylcarnitine+3-hydroxyisovalerylcarnitine; C5, isovalerylcarnitine+methylbutyrylcarnitine; C5-DC+C6-OH, glutarylcarnitine+3-hydroxyhexanoylcarnitine; C6, hexanoylcarnitine; C6-DC, methylglutarylcarnitine; C8, octanoylcarnitine; C8:1, octenoylcarnitine; C10, decanoylcarnitine; C10:1, decenoylcarnitine; C12, dodecanoylcarnitine; C12:1, dodecenoylcarnitine; C14, tetradecanoylcarnitine; C14:1, tetradecenoylcarnitine; C16, palmitoylcarnitine; C16:1, palmitoleylcarnitine; C16:1-OH, 3-hydroxypalmitoleylcarnitine; C18, stearoylcarnitine; C18:1, oleoylcarnitine; C18:2, linoleoylcarnitine.

Table S2. The limits of detetion (LoD), limits of quantification (LoQ) and linear dynamic range of AB Sciex QTRAP 3200 mass spectrometer

Analytes	Linearity (lower limit) (µM)	Linearity (upper limit) (µM)	LoD (µM)	LoQ (µM)
ALA	75.4	1422.4	3.5	13.2
ARG	0.7	237.5	0.1	0.5
CIT	3.8	106.8	1.1	3.9
GLY	97.8	2575.8	3.4	12.2
LEU/ILE/ALLO-ILE/PRO-OH	18.6	991.4	2.7	9.5
MET	5.1	247.5	0.8	2.8
ORN	18.9	968.8	2.5	8.9
PHE	23.1	402.3	2.1	8.2
PRO	43.2	846.6	2.3	8.0
TYR	14.1	668.3	0.5	3.1
VAL	27.2	778.9	0.4	1.8
C0	3.93	177.40	0.90	3.12
C2	3.63	181.23	0.03	0.12
C3	0.34	30.57	<0.01	0.01
C4	0.02	5.24	<0.01	0.01
C5	0.02	6.01	<0.01	0.01
C5-DC	0.02	8.42	<0.01	0.01
C6	0.02	8.92	<0.01	0.01
C8	0.02	7.25	<0.01	0.01
C10	0.02	6.69	<0.01	0.01
C12	0.02	8.83	<0.01	0.01
C14	0.01	6.55	<0.01	0.01
C16	0.02	9.23	<0.01	0.01
C18	0.02	7.34	<0.01	0.01

Abbreviations: LoD, limit of detection; ALA, alanine; ARG, arginine; CIT, citrulline; GLY, glycine; LEU, leucine; ILE, isoleucine; ALLO-ILE, alloisoleucine; PRO-OH, hydroxyproline; MET, methionine; ORN, ornithine; PHE, phenylalanine; PRO, proline; TYR, Tyrosine; VAL, valine; C0, free carnitine; C2, acetylcarnitine; C3, propionylcarnitine; C3-DC+C4-OH, malonylcarnitine+3-hydroxybutyrylcarnitine; C4, butyrylcarnitine+isobutyrylcarnitine; C4-DC+C5-OH, methylmalonylcarnitine+3-hydroxyisovalerylcarnitine; C5, isovalerylcarnitine+methylbutyrylcarnitine; C5-DC, glutarylcarnitine; C5-DC+C6-OH, glutarylcarnitine+3-hydroxyhexanoylcarnitine; C6, hexanoylcarnitine; C6-DC, methylglutarylcarnitine; C8, octanoylcarnitine; C8:1, octenoylcarnitine; C10, decanoylcarnitine; C10:1, decenoylcarnitine; C12, dodecanoylcarnitine; C12:1, dodecenoylcarnitine; C14, tetradecanoylcarnitine; C14:1, tetradecenoylcarnitine; C16, palmitoylcarnitine; C16:1, palmitoleylcarnitine; C16:1-OH, 3-hydroxypalmitoleylcarnitine; C18, stearoylcarnitine; C18:1, oleoylcarnitine; C18:2, linoleoylcarnitine.

Note: the linearity and LoD data for C5-DC also applied to C6-DC; the linearity and LoD data for C4 also applied to C3-DC+C4-OH; the linearity and LoD data for C5 also applied to C4-DC+C5-OH; the linearity and LoD data for C8 also applied to C8:1; the linearity and LoD data for C10 also applied to C10:1; the linearity and LoD data for C12 also applied to C12:1; the linearity and LoD data for C14 also applied to C14:1; the linearity and LoD data for C16 also applied to C16:1 and C16:1-OH; the linearity and LoD data for C18 also applied to C18:1 and C18:2.

Table S3. The limits of detetion (LoD), limits of quantification (LoQ) and linear dynamic range of AB Sciex QTRAP 4000 mass spectrometer

Analytes	Linearity (lower limit) (µM)	Linearity (upper limit) (µM)	LoD (µM)	LoQ (µM)
ALA	71.2	1576.2	2.3	10.2
ARG	0.7	228.6	0.1	0.4
CIT	3.6	101.6	1.0	3.6
GLY	91.5	2696.5	3.2	11.3
LEU/ILE/ALLO-ILE/PRO-OH	17.6	964.8	1.2	5.8
MET	4.8	276.3	0.7	2.4
ORN	16.3	1042.6	1.7	6.2
PHE	18.8	474.2	1.1	5.2
PRO	37.6	925.8	0.9	3.6
TYR	12.5	616.3	0.3	1.6
VAL	23.0	764.3	0.2	0.9
C0	3.62	182.42	0.77	2.92
C2	2.13	168.21	0.02	0.08
C3	0.31	32.20	<0.01	0.01
C4	0.01	6.14	<0.01	0.01
C5	0.01	6.25	<0.01	0.01
C5DC	0.01	7.49	<0.01	0.01
C6	0.01	9.35	<0.01	0.01
C8	0.01	8.75	<0.01	0.01
C10	0.01	8.23	<0.01	0.01
C12	0.01	9.54	<0.01	0.01
C14	0.01	6.65	<0.01	0.01
C16	0.01	9.76	<0.01	0.01
C18	0.01	7.59	<0.01	0.01

Abbreviations: LoD, limit of detection; ALA, alanine; ARG, arginine; CIT, citrulline; GLY, glycine; LEU, leucine; ILE, isoleucine; ALLO-ILE, alloisoleucine; PRO-OH, hydroxyproline; MET, methionine; ORN, ornithine; PHE, phenylalanine; PRO, proline; TYR, Tyrosine; VAL, valine; C0, free carnitine; C2, acetylcarnitine; C3, propionylcarnitine; C3-DC+C4-OH, malonylcarnitine+3-hydroxybutyrylcarnitine; C4, butyrylcarnitine+isobutyrylcarnitine; C4-DC+C5-OH, methylmalonylcarnitine+3-hydroxyisovalerylcarnitine; C5, isovalerylcarnitine+methylbutyrylcarnitine; C5-DC, glutarylcarnitine; C5-DC+C6-OH, glutarylcarnitine+3-hydroxyhexanoylcarnitine; C6, hexanoylcarnitine; C6-DC, methylglutarylcarnitine; C8, octanoylcarnitine; C8:1, octenoylcarnitine; C10, decanoylcarnitine; C10:1, decenoylcarnitine; C12, dodecanoylcarnitine; C12:1, dodecenoylcarnitine; C14, tetradecanoylcarnitine; C14:1, tetradecenoylcarnitine; C16, palmitoylcarnitine; C16:1, palmitoleylcarnitine; C16:1-OH, 3-hydroxypalmitoleylcarnitine; C18, stearoylcarnitine; C18:1, oleoylcarnitine; C18:2, linoleoylcarnitine.

Note: the linearity and LoD data for C5-DC also applied to C6-DC; the linearity and LoD data for C4 also applied to C3-DC+C4-OH; the linearity and LoD data for C5 also applied to C4-DC+C5-OH; the linearity and LoD data for C8 also applied to C8:1; the linearity and LoD data for C10 also applied to C10:1; the linearity and LoD data for C12 also applied to C12:1; the linearity and LoD data for C14 also applied to C14:1; the linearity and LoD data for C16 also applied to C16:1 and C16:1-OH; the linearity and LoD data for C18 also applied to C18:1 and C18:2.

Table S4. The limits of detetion (LoD), limits of quantification (LoQ) and linear dynamic range of AB Sciex QTRAP 4500 mass spectrometer

Analytes	Linearity (lower limit) (µM)	Linearity (upper limit) (µM)	LoD (µM)	LoQ (µM)
ALA	68.3	1516.7	2.1	8.6
ARG	0.6	201.1	0.1	0.4
CIT	3.6	113.1	0.8	3.2
GLY	88.6	2809.2	2.3	9.5
LEU/ILE/ALLO-ILE/PRO-OH	17.1	944.8	0.5	3.1
MET	4.8	289.0	0.3	1.8
ORN	15.8	1035.6	0.6	3.7
PHE	18.8	431.7	0.1	0.8
PRO	35.6	962.2	0.1	0.9
TYR	12.3	589.3	0.3	1.6
VAL	22.3	714.0	0.2	0.9
C0	3.48	175.26	0.05	1.22
C2	1.65	134.43	0.02	0.08
C3	0.28	32.63	<0.01	0.01
C4	0.01	5.62	<0.01	0.01
C5	0.01	7.27	<0.01	0.01
C5DC	0.01	8.34	<0.01	0.01
C6	0.01	10.33	<0.01	0.01
C8	0.01	8.11	<0.01	0.01
C10	0.01	7.95	<0.01	0.01
C12	0.01	9.32	<0.01	0.01
C14	0.01	7.33	<0.01	0.01
C16	0.01	9.53	<0.01	0.01
C18	0.01	8.52	<0.01	0.01

Abbreviations: LoD, limit of detection; ALA, alanine; ARG, arginine; CIT, citrulline; GLY, glycine; LEU, leucine; ILE, isoleucine; ALLO-ILE, alloisoleucine; PRO-OH, hydroxyproline; MET, methionine; ORN, ornithine; PHE, phenylalanine; PRO, proline; TYR, Tyrosine; VAL, valine; C0, free carnitine; C2, acetylcarnitine; C3, propionylcarnitine; C3-DC+C4-OH, malonylcarnitine+3-hydroxybutyrylcarnitine; C4, butyrylcarnitine+isobutyrylcarnitine; C4-DC+C5-OH, methylmalonylcarnitine+3-hydroxyisovalerylcarnitine; C5, isovalerylcarnitine+methylbutyrylcarnitine; C5-DC, glutarylcarnitine; C5-DC+C6-OH, glutarylcarnitine+3-hydroxyhexanoylcarnitine; C6, hexanoylcarnitine; C6-DC, methylglutarylcarnitine; C8, octanoylcarnitine; C8:1, octenoylcarnitine; C10, decanoylcarnitine; C10:1, decenoylcarnitine; C12, dodecanoylcarnitine; C12:1, dodecenoylcarnitine; C14, tetradecanoylcarnitine; C14:1, tetradecenoylcarnitine; C16, palmitoylcarnitine; C16:1, palmitoleylcarnitine; C16:1-OH, 3-hydroxypalmitoleylcarnitine; C18, stearoylcarnitine; C18:1, oleoylcarnitine; C18:2, linoleoylcarnitine.

Note: the linearity and LoD data for C5-DC also applied to C6-DC; the linearity and LoD data for C4 also applied to C3-DC+C4-OH; the linearity and LoD data for C5 also applied to C4-DC+C5-OH; the linearity and LoD data for C8 also applied to C8:1; the linearity and LoD data for C10 also applied to C10:1; the linearity and LoD data for C12 also applied to C12:1; the linearity and LoD data for C14 also applied to C14:1; the linearity and LoD data for C16 also applied to C16:1 and C16:1-OH; the linearity and LoD data for C18 also applied to C18:1 and C18:2.

Table S5. The limits of detetion (LoD), limits of quantification (LoQ) and linear dynamic range of Waters Quattro micro AP mass spectrometer

Analytes	Linearity (lower limit) (μM)	Linearity (upper limit) (μM)	LoD (μM)	LoQ (μM)
ALA	68.5	1596.2	1.8	7.3
ARG	0.7	264.3	0.1	0.5
CIT	3.7	135.3	1.1	3.8
GLY	95.4	2741.4	2.7	12.0
LEU/ILE/ALLO-ILE/PRO-OH	18.5	903.3	1.2	5.2
MET	4.9	273.8	0.7	2.6
ORN	18.6	1214.5	1.3	6.9
PHE	19.3	410.3	0.8	3.7
PRO	37.8	900.6	1.1	4.3
TYR	11.5	638.2	0.5	2.6
VAL	26.9	779.6	0.4	1.8
C0	3.86	165.55	0.88	3.07
C2	2.47	118.37	0.02	0.08
C3	0.29	34.26	<0.01	0.01
C4	0.01	6.62	<0.01	0.01
C5	0.01	6.54	<0.01	0.01
C5DC	0.01	9.62	<0.01	0.01
C6	0.01	9.56	<0.01	0.01
C8	0.01	9.46	<0.01	0.01
C10	0.01	8.56	<0.01	0.01
C12	0.01	9.36	<0.01	0.01
C14	0.01	6.69	<0.01	0.01
C16	0.01	8.81	<0.01	0.01
C18	0.01	7.66	<0.01	0.01

Abbreviations: LoD, limit of detection; ALA, alanine; ARG, arginine; CIT, citrulline; GLY, glycine; LEU, leucine; ILE, isoleucine; ALLO-ILE, alloisoleucine; PRO-OH, hydroxyproline; MET, methionine; ORN, ornithine; PHE, phenylalanine; PRO, proline; TYR, Tyrosine; VAL, valine; C0, free carnitine; C2, acetylcarnitine; C3, propionylcarnitine; C3-DC+C4-OH, malonylcarnitine+3-hydroxybutyrylcarnitine; C4, butyrylcarnitine+isobutyrylcarnitine; C4-DC+C5-OH, methylmalonylcarnitine+3-hydroxyisovalerylcarnitine; C5, isovalerylcarnitine+methylbutyrylcarnitine; C5-DC, glutarylcarnitine; C5-DC+C6-OH, glutarylcarnitine+3-hydroxyhexanoylcarnitine; C6, hexanoylcarnitine; C6-DC, methylglutarylcarnitine; C8, octanoylcarnitine; C8:1, octenoylcarnitine; C10, decanoylcarnitine; C10:1, decenoylcarnitine; C12, dodecanoylcarnitine; C12:1, dodecenoylcarnitine; C14, tetradecanoylcarnitine; C14:1, tetradecenoylcarnitine; C16, palmitoylcarnitine; C16:1, palmitoleylcarnitine; C16:1-OH, 3-hydroxypalmitoleylcarnitine; C18, stearoylcarnitine; C18:1, oleoylcarnitine; C18:2, linoleoylcarnitine.

Note: the linearity and LoD data for C5-DC also applied to C6-DC; the linearity and LoD data for C4 also applied to C3-DC+C4-OH; the linearity and LoD data for C5 also applied to C4-DC+C5-OH; the linearity and LoD data for C8 also applied to C8:1; the linearity and LoD data for C10 also applied to C10:1; the linearity and LoD data for C12 also applied to C12:1; the linearity and LoD data for C14 also applied to C14:1; the linearity and LoD data for C16 also applied to C16:1 and C16:1-OH; the linearity and LoD data for C18 also applied to C18:1 and C18:2.

Table S6. The limits of detetion (LoD), limits of quantification (LoQ) and linear dynamic range of Waters Acquity TQD mass spectrometer

Analytes	Linearity (lower limit) (μM)	Linearity (upper limit) (μM)	LoD (μM)	LoQ (μM)
ALA	66.5	1631.3	1.7	6.3
ARG	0.5	259.4	0.1	0.4
CIT	3.6	128.3	0.8	3.4
GLY	92.1	2657.2	2.5	9.4
LEU/ILE/ALLO-ILE/PRO-OH	17.9	1026.8	0.1	0.6
MET	4.7	259.4	0.5	2.5
ORN	16.7	917.4	0.9	4.1
PHE	18.6	399.6	0.1	0.5
PRO	32.4	896.2	0.1	0.6
TYR	10.6	552.4	0.3	1.2
VAL	23.9	682.2	0.1	0.8
C0	3.12	152.77	0.04	0.32
C2	2.32	156.86	0.02	0.07
C3	0.26	34.46	<0.01	0.01
C4	0.01	6.11	<0.01	0.01
C5	0.01	6.61	<0.01	0.01
C5DC	0.01	7.79	<0.01	0.01
C6	0.01	6.43	<0.01	0.01
C8	0.01	7.42	<0.01	0.01
C10	0.01	7.36	<0.01	0.01
C12	0.01	8.62	<0.01	0.01
C14	0.01	7.45	<0.01	0.01
C16	0.01	8.95	<0.01	0.01
C18	0.01	7.12	<0.01	0.01

Abbreviations: LoD, limit of detection; ALA, alanine; ARG, arginine; CIT, citrulline; GLY, glycine; LEU, leucine; ILE, isoleucine; ALLO-ILE, alloisoleucine; PRO-OH, hydroxyproline; MET, methionine; ORN, ornithine; PHE, phenylalanine; PRO, proline; TYR, Tyrosine; VAL, valine; C0, free carnitine; C2, acetylcarnitine; C3, propionylcarnitine; C3-DC+C4-OH, malonylcarnitine+3-hydroxybutyrylcarnitine; C4, butyrylcarnitine+isobutyrylcarnitine; C4-DC+C5-OH, methylmalonylcarnitine+3-hydroxyisovalerylcarnitine; C5, isovalerylcarnitine+methylbutyrylcarnitine; C5-DC, glutarylcarnitine; C5-DC+C6-OH, glutarylcarnitine+3-hydroxyhexanoylcarnitine; C6, hexanoylcarnitine; C6-DC, methylglutarylcarnitine; C8, octanoylcarnitine; C8:1, octenoylcarnitine; C10, decanoylcarnitine; C10:1, decenoylcarnitine; C12, dodecanoylcarnitine; C12:1, dodecenoylcarnitine; C14, tetradecanoylcarnitine; C14:1, tetradecenoylcarnitine; C16, palmitoylcarnitine; C16:1, palmitoleylcarnitine; C16:1-OH, 3-hydroxypalmitoleylcarnitine; C18, stearoylcarnitine; C18:1, oleoylcarnitine; C18:2, linoleoylcarnitine.

Note: the linearity and LoD data for C5-DC also applied to C6-DC; the linearity and LoD data for C4 also applied to C3-DC+C4-OH; the linearity and LoD data for C5 also applied to C4-DC+C5-OH; the linearity and LoD data for C8 also applied to C8:1; the linearity and LoD data for C10 also applied to C10:1; the linearity and LoD data for C12 also applied to C12:1; the linearity and LoD data for C14 also applied to C14:1; the linearity and LoD data for C16 also applied to C16:1 and C16:1-OH; the linearity and LoD data for C18 also applied to C18:1 and C18:2.

Table S7. The limits of detetion (LoD), limits of quantification (LoQ) and linear dynamic range of Waters Xevo TQD mass spectrometer

Analytes	Linearity (lower limit) (µM)	Linearity (upper limit) (µM)	LoD (µM)	LoQ (µM)
ALA	56.2	1493.3	1.6	5.5
ARG	0.5	248.7	0.1	0.4
CIT	3.5	128.7	0.1	0.5
GLY	87.3	2616.3	1.6	6.2
LEU/ILE/ALLO-ILE/PRO-OH	17.8	919.2	0.1	0.6
MET	4.7	286.3	0.3	2.1
ORN	16.5	986.4	0.2	2.8
PHE	18.1	463.6	0.1	0.5
PRO	32.1	1069.6	0.1	0.5
TYR	9.5	576.5	0.3	1.2
VAL	23.4	762.8	0.1	0.8
C0	3.12	153.92	0.02	0.28
C2	2.03	124.54	0.02	0.07
C3	0.21	33.57	<0.01	0.01
C4	0.01	6.33	<0.01	0.01
C5	0.01	7.87	<0.01	0.01
C5DC	0.01	8.00	<0.01	0.01
C6	0.01	9.33	<0.01	0.01
C8	0.01	7.84	<0.01	0.01
C10	0.01	7.36	<0.01	0.01
C12	0.01	6.47	<0.01	0.01
C14	0.01	7.11	<0.01	0.01
C16	0.01	8.85	<0.01	0.01
C18	0.01	7.52	<0.01	0.01

Abbreviations: LoD, limit of detection; ALA, alanine; ARG, arginine; CIT, citrulline; GLY, glycine; LEU, leucine; ILE, isoleucine; ALLO-ILE, alloisoleucine; PRO-OH, hydroxyproline; MET, methionine; ORN, ornithine; PHE, phenylalanine; PRO, proline; TYR, Tyrosine; VAL, valine; C0, free carnitine; C2, acetylcarnitine; C3, propionylcarnitine; C3-DC+C4-OH, malonylcarnitine+3-hydroxybutyrylcarnitine; C4, butyrylcarnitine+isobutyrylcarnitine; C4-DC+C5-OH, methylmalonylcarnitine+3-hydroxyisovalerylcarnitine; C5, isovalerylcarnitine+methylbutyrylcarnitine; C5-DC, glutarylcarnitine; C5-DC+C6-OH, glutarylcarnitine+3-hydroxyhexanoylcarnitine; C6, hexanoylcarnitine; C6-DC, methylglutarylcarnitine; C8, octanoylcarnitine; C8:1, octenoylcarnitine; C10, decanoylcarnitine; C10:1, decenoylcarnitine; C12, dodecanoylcarnitine; C12:1, dodecenoylcarnitine; C14, tetradecanoylcarnitine; C14:1, tetradecenoylcarnitine; C16, palmitoylcarnitine; C16:1, palmitoleylcarnitine; C16:1-OH, 3-hydroxypalmitoleylcarnitine; C18, stearoylcarnitine; C18:1, oleoylcarnitine; C18:2, linoleoylcarnitine.

Note: the linearity and LoD data for C5-DC also applied to C6-DC; the linearity and LoD data for C4 also applied to C3-DC+C4-OH; the linearity and LoD data for C5 also applied to C4-DC+C5-OH; the linearity and LoD data for C8 also applied to C8:1; the linearity and LoD data for C10 also applied to C10:1; the linearity and LoD data for C12 also applied to C12:1; the linearity and LoD data for C14 also applied to C14:1; the linearity and LoD data for C16 also applied to C16:1 and C16:1-OH; the linearity and LoD data for C18 also applied to C18:1 and C18:2.

Table S8. The total imprecision of test analytes

Analytes	total imprecision
ALA	5.2%-8.0%
ARG	5.9%-9.1%
CIT	5.5%-8.2%
GLY	5.5%-8.9%
LEU/ILE/ALLO-ILE/PRO-OH	5.1%-7.7%
MET	5.2%-8.0%
ORN	5.4%-8.2%
PHE	4.9%-7.7%
PRO	5.7%-7.6%
TYR	4.9%-7.8%
VAL	5.4%-8.1%
C0	5.2%-8.3%
C2	4.9%-8.4%
C3	5.2%-8.0%
C4	5.3%-8.3%
C5	5.4%-8.5%
C5-DC	6.4%-10.0%
C6	5.4%-8.6%
C8	5.5%-8.8%
C10	5.4%-9.0%
C12	5.3%-8.7%
C14	5.5%-8.9%
C16	5.4%-8.1%
C18	5.5%-8.0%

Abbreviations: ALA, alanine; ARG, arginine; CIT, citrulline; GLY, glycine; LEU, leucine; ILE, isoleucine; ALLO-ILE, alloisoleucine; PRO-OH, hydroxyproline; MET, methionine; ORN, ornithine; PHE, phenylalanine; PRO, proline; TYR, Tyrosine; VAL, valine; C0, free carnitine; C2, acetylcarnitine; C3, propionylcarnitine; C4, butyrylcarnitine+isobutyrylcarnitine; C5, isovalerylcarnitine+methylbutyrylcarnitine; C5-DC, glutarylcarnitine; C6, hexanoylcarnitine; C8, octanoylcarnitine; C10, decanoylcarnitine; C12, dodecanoylcarnitine; C14, tetradecanoylcarnitine; C16, palmitoylcarnitine; C18, stearoylcarnitine

Table S9. The sample collection data at each province/municipality

Provinces/Municipalities	Date
Beijing	January 1, 2017, to December 31, 2018
Shanghai	May 1, 2015, to December 15, 2018
Chongqing	March 25, 2017, to December 29, 2018
Hebei	January 12, 2016, to November 23, 2018
Shanxi	January 12, 2016, to November 3, 2018
Liaoning	January 1, 2017, to December 31, 2018
Jilin	May 11, 2016, to October 30, 2018
Heilongjiang	January 1, 2017, to December 31, 2017
Jiangsu	December 1, 2015, to December 1, 2018
Zhejiang	January 15, 2015, to November 30, 2018
Anhui	May 20, 2016, to November 12, 2018
Fujian	February 15, 2016, to December 3, 2018
Jiangxi	January 6, 2016, to December 25, 2018
Shandong	November 1, 2015, to December 1, 2018
Henan	January 23, 2016, to October 30, 2018
Hubei	January 1, 2017, to December 31, 2018
Hunan	January 11, 2016, to November 5, 2018
Guangdong	January 29, 2016, to November 18, 2018
Guangxi	January 16, 2016, to November 16, 2018
Hainan	December 1, 2016, to November 30, 2018
Sichuan	January 21, 2016, to November 25, 2018
Guizhou	January 1, 2017, to December 31, 2018
Yunnan	February 5, 2017, to November 29, 2018
Shaanxi	February 1, 2016, to November 30, 2018
Gansu	May 22, 2016 to October 30, 2018
Ningxia	November 1, 2015, to November 3, 2018
Xinjiang	June 11, 2017, to December 20, 2018

Table S10. The 1st, 25th, 50th, 75th and 99th percentiles calculated by each day and sex for 35 MS/MS NBS biomarkers (μM)

Analyte	Age	Amino acids									
		Male					Female				
		1st	25th	50th	75th	99th	1st	25th	50th	75th	99th
ALA	0 to ≤ 1 day	**157.6**	**228.8**	**266.6**	**312.0**	**420.8**	**158.1**	**232.5**	**270.3**	**315.7**	**423.7**
	1 day to ≤ 2 days	**148.1**	**228.7**	**272.9**	**325.8**	**456.4**	**153.2**	**236.0**	**280.5**	**335.2**	**468.5**
	2 days to ≤ 3 days	**161.4**	**251.3**	**302.6**	**365.3**	**519.3**	**167.8**	**259.8**	**312.5**	**377.0**	**534.6**
	3 days to ≤ 4 days	**170.3**	**267.2**	**322.6**	**389.6**	**553.0**	**177.6**	**277.2**	**334.6**	**403.4**	**571.6**
	4 days to ≤ 5 days	**168.8**	**271.3**	**326.3**	**390.2**	**545.1**	**177.8**	**283.0**	**340.2**	**405.7**	**565.6**
	5 days to ≤ 6 days	**167.5**	**271.5**	**324.8**	**384.6**	**533.4**	**175.9**	**282.9**	**337.2**	**399.0**	**551.2**
	6 days to ≤ 7 days	**164.3**	**266.7**	**318.5**	**376.4**	**519.0**	**176.2**	**278.4**	**330.5**	**389.2**	**534.9**
	7 days to ≤ 8 days	**161.0**	**262.1**	**312.3**	**370.0**	**512.1**	**168.4**	**273.4**	**324.2**	**382.2**	**525.8**
	8 days to ≤ 9 days	**161.3**	**260.8**	**309.6**	**365.9**	**504.7**	**169.7**	**271.0**	**321.1**	**378.6**	**520.8**
	9 days to ≤ 10 days	**161.0**	**256.9**	**307.3**	**363.7**	**503.0**	**169.1**	**270.0**	**320.6**	**376.1**	**514.5**
	10 days to ≤ 11 days	**162.6**	**253.0**	**301.9**	**357.2**	**495.5**	**170.8**	**266.4**	**315.8**	**372.4**	**510.8**
	11 days to ≤ 12 days	**161.8**	**252.9**	**300.4**	**353.7**	**488.0**	**168.5**	**261.0**	**308.9**	**363.5**	**501.6**
	12 days to ≤ 13 days	**158.8**	**252.6**	**299.6**	**354.3**	**488.4**	**169.8**	**261.3**	**306.9**	**360.7**	**491.0**
	13 days to ≤ 14 days	**156.0**	**251.0**	**297.7**	**352.6**	**488.9**	**167.9**	**258.8**	**305.2**	**358.5**	**486.5**
ARG	0 to ≤ 1 day	**1.2**	**4.8**	**8.1**	**12.5**	**22.9**	**1.2**	**4.4**	**7.4**	**11.3**	**20.9**
	1 day to ≤ 2 days	**1.2**	**4.4**	**8.1**	**13.2**	**25.6**	**1.1**	**4.0**	**7.3**	**12.3**	**24.1**
	2 days to ≤ 3 days	**1.2**	**4.8**	**8.8**	**14.3**	**27.2**	**1.2**	**4.4**	**8.1**	**13.3**	**25.5**
	3 days to ≤ 4 days	**1.3**	**5.4**	**9.9**	**15.9**	**30.1**	**1.3**	**5.0**	**9.2**	**15.0**	**28.7**
	4 days to ≤ 5 days	**1.3**	**6.0**	**11.1**	**17.6**	**33.1**	**1.3**	**5.6**	**10.5**	**16.8**	**32.1**
	5 days to ≤ 6 days	**1.3**	**6.1**	**11.2**	**17.7**	**33.3**	**1.3**	**5.6**	**10.5**	**16.7**	**31.6**
	6 days to ≤ 7 days	**1.2**	**6.0**	**11.1**	**17.7**	**33.4**	**1.2**	**5.5**	**10.4**	**16.6**	**31.5**
	7 days to ≤ 8 days	**1.3**	**6.1**	**11.4**	**17.9**	**33.5**	**1.2**	**5.6**	**10.5**	**16.6**	**31.7**
	8 days to ≤ 9 days	**1.3**	**6.3**	**11.7**	**18.4**	**34.7**	**1.3**	**5.8**	**10.9**	**17.4**	**32.7**
	9 days to ≤ 10 days	**1.4**	**6.8**	**12.3**	**19.1**	**35.8**	**1.3**	**6.3**	**11.5**	**18.0**	**33.6**
	10 days to ≤ 11 days	**1.5**	**7.2**	**13.1**	**20.3**	**37.7**	**1.4**	**6.6**	**12.1**	**19.0**	**35.8**

	11 days to ≤ 12 days	**1.5**	**7.9**	**14.0**	**21.6**	**40.0**	**1.5**	**7.2**	**12.8**	**19.9**	**36.8**
	12 days to ≤ 13 days	**1.6**	**8.4**	**14.9**	**22.5**	**41.3**	**1.5**	**7.6**	**13.5**	**20.9**	**38.3**
	13 days to ≤ 14 days	**1.7**	**8.8**	**15.1**	**23.3**	**42.8**	**1.6**	**8.1**	**14.5**	**22.0**	**40.2**
CIT	0 to ≤ 1 day	**6.9**	**10.9**	**12.9**	**15.2**	**20.9**	**7.1**	**11.1**	**13.1**	**15.4**	**21.1**
	1 day to ≤ 2 days	**6.7**	**10.6**	**12.7**	**15.2**	**21.2**	**6.9**	**10.9**	**13.0**	**15.5**	**21.7**
	2 days to ≤ 3 days	**6.9**	**10.9**	**13.2**	**15.8**	**22.3**	**7.1**	**11.3**	**13.6**	**16.3**	**23.0**
	3 days to ≤ 4 days	**7.0**	**11.0**	**13.2**	**15.8**	**22.2**	**7.3**	**11.5**	**13.8**	**16.5**	**23.1**
	4 days to ≤ 5 days	**6.8**	**10.8**	**12.9**	**15.4**	**21.5**	**7.2**	**11.3**	**13.5**	**16.0**	**22.4**
	5 days to ≤ 6 days	**6.9**	**11.0**	**13.0**	**15.5**	**21.5**	**7.1**	**11.4**	**13.6**	**16.2**	**22.5**
	6 days to ≤ 7 days	**6.7**	**11.0**	**13.2**	**15.8**	**22.1**	**7.0**	**11.5**	**13.8**	**16.3**	**22.7**
	7 days to ≤ 8 days	**6.8**	**11.3**	**13.6**	**16.2**	**22.7**	**7.0**	**11.7**	**14.0**	**16.7**	**23.4**
	8 days to ≤ 9 days	**6.7**	**11.6**	**14.0**	**16.8**	**23.6**	**7.0**	**12.0**	**14.4**	**17.3**	**24.3**
	9 days to ≤ 10 days	**6.9**	**11.9**	**14.4**	**17.3**	**24.4**	**7.2**	**12.3**	**14.8**	**17.8**	**25.0**
	10 days to ≤ 11 days	**7.0**	**12.2**	**14.9**	**17.8**	**25.0**	**7.3**	**12.5**	**15.2**	**18.3**	**25.9**
	11 days to ≤ 12 days	**7.6**	**12.7**	**15.2**	**18.2**	**25.6**	**7.5**	**12.9**	**15.5**	**18.5**	**26.1**
	12 days to ≤ 13 days	**7.5**	**12.9**	**15.5**	**18.5**	**26.1**	**8.0**	**13.2**	**15.9**	**19.0**	**26.8**
	13 days to ≤ 14 days	**7.6**	**13.0**	**15.7**	**18.9**	**26.6**	**7.8**	**13.4**	**16.2**	**19.4**	**27.2**
GLY	0 to ≤ 1 day	**280.8**	**408.9**	**469.7**	**537.7**	**707.4**	**289.9**	**421.0**	**480.2**	**547.6**	**715.8**
	1 day to ≤ 2 days	**265.4**	**393.3**	**463.8**	**550.9**	**770.1**	**275.7**	**406.5**	**477.3**	**565.4**	**785.0**
	2 days to ≤ 3 days	**259.8**	**400.8**	**477.2**	**570.3**	**801.2**	**270.7**	**414.4**	**491.1**	**583.3**	**812.7**
	3 days to ≤ 4 days	**247.9**	**380.6**	**454.4**	**545.1**	**769.7**	**260.0**	**395.7**	**470.0**	**560.8**	**785.1**
	4 days to ≤ 5 days	**217.1**	**325.8**	**387.8**	**465.6**	**658.2**	**228.7**	**340.2**	**403.0**	**481.4**	**674.4**
	5 days to ≤ 6 days	**203.5**	**302.3**	**358.4**	**429.9**	**605.8**	**212.8**	**314.4**	**370.9**	**442.2**	**618.3**
	6 days to ≤ 7 days	**196.3**	**289.3**	**341.8**	**408.3**	**573.6**	**203.0**	**300.0**	**353.7**	**421.8**	**590.6**
	7 days to ≤ 8 days	**191.8**	**284.1**	**334.2**	**399.3**	**559.3**	**201.0**	**293.7**	**346.3**	**412.8**	**578.9**
	8 days to ≤ 9 days	**189.6**	**280.2**	**328.8**	**389.6**	**546.3**	**197.0**	**289.8**	**338.8**	**401.0**	**559.0**
	9 days to ≤ 10 days	**191.8**	**277.7**	**324.8**	**383.7**	**533.9**	**197.3**	**286.3**	**333.7**	**394.2**	**546.3**
	10 days to ≤ 11 days	**188.5**	**273.9**	**321.0**	**380.4**	**529.6**	**196.0**	**281.3**	**327.3**	**387.1**	**536.9**
	11 days to ≤ 12 days	**184.5**	**268.4**	**313.0**	**368.2**	**507.7**	**189.7**	**275.2**	**319.5**	**375.2**	**512.6**
	12 days to ≤ 13 days	**185.6**	**267.2**	**310.2**	**364.0**	**501.8**	**189.3**	**273.0**	**317.5**	**374.4**	**514.3**
	13 days to ≤ 14 days	**185.5**	**261.9**	**304.1**	**360.5**	**500.6**	**187.5**	**268.0**	**311.5**	**365.2**	**503.1**
LEU/ILE/ALLO-ILE/PRO-OH	0 to ≤ 1 day	56.4	83.4	105.0	137.3	207.9	56.4	83.4	105.0	137.3	207.9

	1 day to ≤ 2 days	**68.0**	**111.3**	**133.6**	**158.9**	**221.0**	**68.6**	**113.2**	**136.3**	**162.0**	**225.7**
	2 days to ≤ 3 days	**83.5**	**127.5**	**150.2**	**176.1**	**239.9**	**85.6**	**130.9**	**154.0**	**180.5**	**245.4**
	3 days to ≤ 4 days	**89.0**	**136.4**	**160.6**	**188.6**	**256.9**	**91.7**	**139.8**	**164.5**	**192.9**	**262.4**
	4 days to ≤ 5 days	**87.6**	**138.6**	**164.9**	**195.2**	**269.5**	**89.8**	**141.8**	**168.5**	**199.3**	**274.5**
	5 days to ≤ 6 days	**88.4**	**142.1**	**170.4**	**202.9**	**282.0**	**91.0**	**145.2**	**174.1**	**207.1**	**287.5**
	6 days to ≤ 7 days	**87.3**	**143.7**	**172.6**	**206.0**	**286.9**	**90.7**	**147.4**	**176.8**	**210.2**	**292.0**
	7 days to ≤ 8 days	**89.2**	**145.3**	**175.1**	**208.9**	**291.1**	**90.9**	**148.5**	**178.9**	**213.2**	**296.2**
	8 days to ≤ 9 days	**89.9**	**146.0**	**175.8**	**209.3**	**291.0**	**92.6**	**150.6**	**180.8**	**214.4**	**298.6**
	9 days to ≤ 10 days	**89.8**	**145.8**	**175.9**	**209.9**	**291.8**	**93.2**	**151.5**	**180.3**	**214.4**	**295.7**
	10 days to ≤ 11 days	**90.2**	**146.1**	**175.4**	**208.8**	**291.6**	**94.8**	**150.9**	**180.9**	**214.6**	**297.1**
	11 days to ≤ 12 days	**90.7**	**146.8**	**175.5**	**208.5**	**286.5**	**95.4**	**151.2**	**181.6**	**214.6**	**295.7**
	12 days to ≤ 13 days	**89.5**	**147.6**	**177.0**	**209.3**	**288.5**	**95.5**	**152.3**	**181.9**	**214.5**	**296.1**
	13 days to ≤ 14 days	**92.4**	**148.0**	**177.1**	**209.9**	**287.7**	**93.1**	**152.3**	**182.1**	**215.3**	**295.3**
MET	0 to ≤ 1 day	10.3	16.9	20.1	23.7	32.6	10.3	16.9	20.1	23.7	32.6
	1 day to ≤ 2 days	**9.1**	**15.8**	**19.2**	**23.1**	**32.6**	**9.3**	**16.3**	**19.8**	**23.9**	**34.0**
	2 days to ≤ 3 days	**7.8**	**14.3**	**17.8**	**22.0**	**32.2**	**8.2**	**15.0**	**18.7**	**23.1**	**33.6**
	3 days to ≤ 4 days	**7.8**	**14.4**	**18.0**	**22.5**	**33.0**	**8.3**	**15.2**	**19.0**	**23.6**	**34.6**
	4 days to ≤ 5 days	**7.5**	**14.2**	**17.9**	**22.1**	**32.3**	**7.8**	**15.0**	**18.8**	**23.1**	**33.6**
	5 days to ≤ 6 days	**7.2**	**14.2**	**17.8**	**21.9**	**31.7**	**7.6**	**14.9**	**18.6**	**22.8**	**32.8**
	6 days to ≤ 7 days	**7.2**	**14.4**	**18.0**	**22.0**	**31.7**	**7.6**	**15.0**	**18.7**	**22.7**	**32.5**
	7 days to ≤ 8 days	**7.5**	**14.6**	**18.3**	**22.4**	**32.5**	**7.9**	**15.2**	**19.0**	**23.1**	**33.1**
	8 days to ≤ 9 days	**7.7**	**14.9**	**18.7**	**23.0**	**33.2**	**8.1**	**15.5**	**19.4**	**23.6**	**34.0**
	9 days to ≤ 10 days	**8.1**	**15.3**	**19.1**	**23.4**	**34.0**	**8.2**	**15.9**	**19.8**	**24.1**	**34.9**
	10 days to ≤ 11 days	**8.2**	**15.6**	**19.5**	**24.1**	**35.2**	**8.3**	**16.2**	**20.3**	**24.7**	**35.5**
	11 days to ≤ 12 days	**8.6**	**16.0**	**20.1**	**24.5**	**35.3**	**8.4**	**16.5**	**20.6**	**25.1**	**36.2**
	12 days to ≤ 13 days	**8.4**	**16.4**	**20.4**	**25.1**	**36.1**	**8.6**	**16.8**	**20.9**	**25.5**	**36.9**
	13 days to ≤ 14 days	**8.6**	**16.6**	**20.7**	**25.5**	**37.0**	**9.1**	**17.0**	**21.2**	**26.0**	**37.2**
ORN	0 to ≤ 1 day	**39.1**	**62.5**	**78.3**	**101.8**	**158.5**	**39.1**	**61.7**	**77.0**	**98.8**	**151.2**
	1 day to ≤ 2 days	**45.0**	**74.8**	**93.0**	**117.1**	**178.3**	**44.4**	**74.2**	**92.5**	**116.3**	**176.8**
	2 days to ≤ 3 days	**54.2**	**92.2**	**114.9**	**143.6**	**215.4**	**54.9**	**92.7**	**114.8**	**142.6**	**211.9**
	3 days to ≤ 4 days	**59.7**	**100.7**	**124.6**	**154.9**	**230.1**	**61.0**	**102.0**	**125.7**	**155.3**	**228.9**
	4 days to ≤ 5 days	**58.5**	**99.8**	**122.9**	**151.7**	**222.7**	**59.7**	**101.0**	**124.0**	**152.2**	**222.5**

	5 days to ≤ 6 days	**56.5**	**97.4**	**120.4**	**148.7**	**219.0**	**58.1**	**98.2**	**120.9**	**148.8**	**218.0**
	6 days to ≤ 7 days	**54.0**	**94.2**	**116.5**	**143.2**	**210.2**	**55.6**	**95.0**	**116.5**	**143.5**	**210.2**
	7 days to ≤ 8 days	**53.8**	**94.2**	**115.4**	**141.9**	**207.6**	**54.7**	**93.4**	**114.9**	**140.5**	**205.9**
	8 days to ≤ 9 days	53.4	94.0	115.8	141.6	206.7	53.4	94.0	115.8	141.6	206.7
	9 days to ≤ 10 days	55.0	94.9	115.8	141.5	205.0	55.0	94.9	115.8	141.5	205.0
	10 days to ≤ 11 days	56.2	95.7	116.9	143.0	208.1	56.2	95.7	116.9	143.0	208.1
	11 days to ≤ 12 days	**56.5**	**97.1**	**118.1**	**144.0**	**207.8**	**57.3**	**96.7**	**117.3**	**142.0**	**204.1**
	12 days to ≤ 13 days	57.6	98.8	120.2	145.9	209.8	57.6	98.8	120.2	145.9	209.8
	13 days to ≤ 14 days	59.2	100.0	121.1	146.2	209.4	59.2	100.0	121.1	146.2	209.4
PHE	0 to ≤ 1 day	**34.0**	**47.6**	**53.9**	**60.9**	**78.3**	**34.2**	**46.8**	**52.9**	**59.7**	**76.4**
	1 day to ≤ 2 days	32.1	44.9	51.2	58.6	77.0	32.1	44.9	51.2	58.6	77.0
	2 days to ≤ 3 days	**32.6**	**46.5**	**53.6**	**61.7**	**81.7**	**32.7**	**46.8**	**53.9**	**62.1**	**82.2**
	3 days to ≤ 4 days	**32.0**	**46.0**	**53.1**	**61.3**	**81.3**	**32.1**	**46.2**	**53.3**	**61.5**	**81.6**
	4 days to ≤ 5 days	**29.3**	**42.4**	**49.1**	**57.0**	**76.3**	**29.4**	**42.5**	**49.3**	**57.2**	**76.6**
	5 days to ≤ 6 days	**28.5**	**41.6**	**48.4**	**56.3**	**75.8**	**28.8**	**42.0**	**48.6**	**56.5**	**75.8**
	6 days to ≤ 7 days	**27.7**	**40.7**	**47.5**	**55.5**	**75.1**	**28.0**	**41.1**	**47.8**	**55.8**	**75.1**
	7 days to ≤ 8 days	**27.1**	**40.5**	**47.3**	**55.2**	**74.6**	**27.6**	**40.8**	**47.6**	**55.7**	**75.4**
	8 days to ≤ 9 days	**26.9**	**40.1**	**46.9**	**54.8**	**74.4**	**27.7**	**40.8**	**47.6**	**55.6**	**75.0**
	9 days to ≤ 10 days	**26.8**	**39.9**	**46.5**	**54.3**	**73.6**	**27.6**	**40.6**	**47.2**	**55.4**	**75.2**
	10 days to ≤ 11 days	**26.9**	**39.4**	**45.9**	**53.4**	**72.0**	**27.2**	**40.2**	**46.8**	**54.5**	**73.2**
	11 days to ≤ 12 days	**27.1**	**39.1**	**45.6**	**53.0**	**71.2**	**27.3**	**40.0**	**46.4**	**54.1**	**72.6**
	12 days to ≤ 13 days	**27.1**	**39.1**	**45.4**	**52.9**	**71.1**	**27.7**	**40.0**	**46.4**	**54.2**	**73.1**
	13 days to ≤ 14 days	**27.0**	**38.8**	**44.9**	**52.2**	**70.1**	**27.8**	**40.0**	**46.2**	**53.3**	**71.3**
PRO	0 to ≤ 1 day	**102.6**	**141.0**	**161.9**	**185.8**	**246.0**	**99.9**	**137.7**	**158.1**	**182.2**	**242.7**
	1 day to ≤ 2 days	**100.3**	**148.4**	**173.2**	**202.1**	**273.7**	**100.9**	**148.7**	**173.9**	**203.2**	**275.3**
	2 days to ≤ 3 days	**109.8**	**163.4**	**191.6**	**224.8**	**306.9**	**111.9**	**165.0**	**193.0**	**226.2**	**307.8**
	3 days to ≤ 4 days	**114.1**	**170.0**	**199.7**	**235.4**	**323.7**	**116.9**	**172.1**	**201.8**	**237.6**	**325.9**
	4 days to ≤ 5 days	**108.7**	**164.7**	**195.2**	**232.5**	**324.6**	**111.6**	**167.5**	**198.7**	**236.2**	**328.7**
	5 days to ≤ 6 days	**106.9**	**165.7**	**197.7**	**236.5**	**331.5**	**110.7**	**168.7**	**201.0**	**240.0**	**334.7**
	6 days to ≤ 7 days	**105.5**	**165.4**	**197.8**	**236.8**	**331.3**	**110.1**	**169.8**	**201.3**	**239.8**	**333.1**
	7 days to ≤ 8 days	**106.2**	**165.0**	**196.7**	**234.7**	**329.0**	**109.0**	**168.4**	**200.4**	**237.9**	**330.3**
	8 days to ≤ 9 days	**106.1**	**164.8**	**195.6**	**232.1**	**321.9**	**107.8**	**168.1**	**199.7**	**237.0**	**328.7**

	9 days to ≤ 10 days	**105.9**	**163.7**	**194.5**	**230.9**	**320.2**	**110.2**	**168.5**	**199.0**	**235.4**	**324.2**
	10 days to ≤ 11 days	**104.0**	**163.9**	**194.1**	**229.6**	**316.6**	**110.0**	**167.2**	**198.2**	**233.2**	**320.0**
	11 days to ≤ 12 days	**106.8**	**163.7**	**193.2**	**227.7**	**312.6**	**110.1**	**167.0**	**196.6**	**231.5**	**317.9**
	12 days to ≤ 13 days	**107.0**	**163.6**	**193.4**	**227.4**	**311.9**	**110.8**	**166.5**	**195.7**	**229.5**	**313.1**
	13 days to ≤ 14 days	**105.4**	**164.0**	**193.0**	**227.4**	**309.2**	**108.9**	**165.6**	**194.8**	**228.5**	**310.1**
TYR	0 to ≤ 1 day	**40.7**	**68.8**	**85.6**	**106.9**	**159.6**	**39.7**	**67.7**	**84.8**	**105.9**	**157.3**
	1 day to ≤ 2 days	39.9	71.5	90.3	113.6	170.8	39.9	71.5	90.3	113.6	170.8
	2 days to ≤ 3 days	**43.5**	**77.8**	**98.1**	**123.3**	**185.8**	**44.5**	**79.0**	**99.4**	**124.7**	**186.8**
	3 days to ≤ 4 days	**46.1**	**83.0**	**104.8**	**131.9**	**199.0**	**47.8**	**85.4**	**107.8**	**135.4**	**203.6**
	4 days to ≤ 5 days	**43.8**	**81.7**	**104.0**	**132.0**	**200.5**	**45.2**	**84.7**	**108.2**	**137.2**	**208.9**
	5 days to ≤ 6 days	**41.7**	**80.2**	**102.4**	**129.9**	**198.1**	**43.4**	**83.7**	**107.2**	**135.8**	**206.4**
	6 days to ≤ 7 days	**39.7**	**77.6**	**99.4**	**125.8**	**191.5**	**41.7**	**81.7**	**104.3**	**131.3**	**197.8**
	7 days to ≤ 8 days	**38.6**	**75.5**	**97.0**	**121.5**	**182.2**	**40.9**	**79.6**	**101.5**	**127.0**	**190.4**
	8 days to ≤ 9 days	**38.1**	**74.0**	**93.8**	**117.6**	**175.9**	**41.5**	**78.9**	**99.7**	**123.1**	**181.1**
	9 days to ≤ 10 days	**37.9**	**73.5**	**92.2**	**114.3**	**169.2**	**41.7**	**78.3**	**98.2**	**121.0**	**176.9**
	10 days to ≤ 11 days	**38.5**	**73.4**	**92.0**	**113.3**	**166.9**	**41.6**	**78.3**	**97.2**	**120.8**	**176.2**
	11 days to ≤ 12 days	**39.4**	**73.4**	**91.0**	**111.9**	**162.9**	**41.9**	**77.8**	**96.8**	**119.6**	**174.6**
	12 days to ≤ 13 days	**39.3**	**74.4**	**92.1**	**112.5**	**162.4**	**42.7**	**79.6**	**98.1**	**119.7**	**172.7**
	13 days to ≤ 14 days	**39.2**	**74.1**	**91.7**	**112.0**	**160.9**	**41.4**	**79.5**	**98.7**	**120.2**	**174.5**
VAL	0 to ≤ 1 day	51.9	75.9	92.1	115.4	168.9	51.9	75.9	92.1	115.4	168.9
	1 day to ≤ 2 days	**58.4**	**92.9**	**111.2**	**133.2**	**187.4**	**59.6**	**95.4**	**114.7**	**137.5**	**193.6**
	2 days to ≤ 3 days	**71.5**	**111.5**	**132.0**	**155.7**	**214.0**	**74.0**	**115.8**	**136.9**	**161.2**	**221.0**
	3 days to ≤ 4 days	**79.1**	**120.2**	**141.0**	**165.1**	**224.5**	**82.7**	**124.8**	**146.2**	**170.8**	**231.4**
	4 days to ≤ 5 days	**78.7**	**120.6**	**141.7**	**166.1**	**226.5**	**81.9**	**125.3**	**146.9**	**171.9**	**233.5**
	5 days to ≤ 6 days	**77.7**	**121.6**	**144.1**	**170.1**	**233.4**	**81.9**	**126.4**	**149.6**	**176.3**	**241.3**
	6 days to ≤ 7 days	**75.4**	**120.8**	**144.0**	**170.7**	**235.8**	**79.3**	**126.0**	**150.2**	**178.0**	**245.2**
	7 days to ≤ 8 days	**74.9**	**120.8**	**144.5**	**171.6**	**237.0**	**78.1**	**125.9**	**150.4**	**178.3**	**245.3**
	8 days to ≤ 9 days	**74.1**	**120.5**	**144.0**	**170.6**	**235.8**	**79.7**	**126.6**	**151.2**	**178.6**	**246.9**
	9 days to ≤ 10 days	**74.2**	**119.5**	**143.3**	**170.5**	**237.4**	**79.1**	**126.7**	**151.0**	**178.5**	**245.2**
	10 days to ≤ 11 days	**74.5**	**119.0**	**142.2**	**168.7**	**232.0**	**79.7**	**126.3**	**150.5**	**177.7**	**244.3**
	11 days to ≤ 12 days	**73.8**	**118.9**	**141.8**	**168.2**	**231.4**	**80.1**	**125.8**	**150.9**	**177.1**	**244.2**
	12 days to ≤ 13 days	**74.2**	**119.7**	**143.5**	**169.1**	**232.3**	**79.6**	**126.5**	**150.4**	**177.9**	**245.0**

	13 days to ≤ 14 days	75.6	119.4	142.7	168.4	230.4	80.1	126.9	150.4	177.5	242.3
		**Acylcarnitnes**									
C0	0 to ≤ 1 day	9.33	15.65	19.24	23.85	34.87	8.90	14.67	17.99	21.79	31.31
	1 day to ≤ 2 days	9.07	15.91	19.88	24.68	36.51	8.52	14.79	18.42	22.79	33.47
	2 days to ≤ 3 days	10.08	17.47	21.80	27.00	39.73	9.55	16.19	20.15	24.89	36.52
	3 days to ≤ 4 days	10.51	18.29	22.68	27.86	40.59	9.91	16.99	21.03	25.80	37.48
	4 days to ≤ 5 days	10.42	18.33	22.63	27.72	40.20	9.79	17.11	21.18	25.92	37.52
	5 days to ≤ 6 days	10.28	18.42	22.82	28.00	40.67	9.82	17.42	21.60	26.44	38.34
	6 days to ≤ 7 days	10.24	18.35	22.85	28.00	40.70	9.87	17.49	21.64	26.55	38.37
	7 days to ≤ 8 days	10.28	18.53	23.07	28.31	41.14	9.83	17.74	22.00	27.01	39.28
	8 days to ≤ 9 days	10.53	18.84	23.49	28.84	41.83	10.00	18.16	22.63	27.64	39.97
	9 days to ≤ 10 days	10.66	19.08	23.76	29.06	42.24	9.92	18.41	22.90	27.95	40.41
	10 days to ≤ 11 days	10.53	19.25	24.08	29.63	43.06	10.08	18.96	23.33	28.51	41.12
	11 days to ≤ 12 days	10.81	19.75	24.56	29.94	43.18	10.37	19.38	23.97	29.07	41.97
	12 days to ≤ 13 days	10.95	20.13	24.91	30.39	43.51	10.92	19.86	24.45	29.53	41.89
	13 days to ≤ 14 days	11.44	20.66	25.46	30.98	44.35	10.75	20.17	24.91	29.95	42.56
C2	0 to ≤ 1 day	7.84	14.88	18.33	22.53	32.73	7.64	13.86	17.05	20.75	30.04
	1 day to ≤ 2 days	8.20	15.64	19.35	23.71	34.50	7.51	14.40	17.85	21.93	32.00
	2 days to ≤ 3 days	7.09	15.00	18.87	23.36	34.51	6.60	13.93	17.50	21.71	32.05
	3 days to ≤ 4 days	6.80	14.25	17.85	22.03	32.39	6.44	13.32	16.69	20.65	30.40
	4 days to ≤ 5 days	5.92	12.43	15.61	19.28	28.39	5.66	11.77	14.72	18.20	26.82
	5 days to ≤ 6 days	5.33	11.25	14.08	17.40	25.54	5.15	10.79	13.49	16.65	24.42
	6 days to ≤ 7 days	4.83	10.12	12.69	15.64	22.97	4.77	9.74	12.17	15.02	22.02
	7 days to ≤ 8 days	4.44	9.25	11.63	14.42	21.33	4.46	8.99	11.21	13.89	20.51
	8 days to ≤ 9 days	4.12	8.71	10.93	13.52	20.00	4.10	8.47	10.65	13.17	19.52
	9 days to ≤ 10 days	3.98	8.37	10.52	13.05	19.34	4.02	8.06	10.16	12.55	18.53
	10 days to ≤ 11 days	3.90	8.13	10.19	12.66	18.58	3.97	7.96	9.90	12.23	17.99
	11 days to ≤ 12 days	3.88	8.06	10.11	12.49	18.26	3.81	7.82	9.79	12.00	17.47
	12 days to ≤ 13 days	4.02	8.17	10.19	12.52	18.28	3.84	7.90	9.79	12.00	17.27
	13 days to ≤ 14 days	4.17	8.16	10.16	12.55	18.27	3.94	7.90	9.85	12.11	17.66
C3	0 to ≤ 1 day	0.69	1.25	1.58	1.98	2.97	0.67	1.19	1.50	1.87	2.79
	1 day to ≤ 2 days	0.68	1.23	1.56	1.98	3.02	0.65	1.18	1.50	1.90	2.89

	2 days to ≤ 3 days	**0.66**	**1.24**	**1.59**	**2.03**	**3.11**	**0.64**	**1.20**	**1.53**	**1.96**	**3.00**
	3 days to ≤ 4 days	**0.65**	**1.20**	**1.52**	**1.93**	**2.93**	**0.62**	**1.16**	**1.47**	**1.87**	**2.84**
	4 days to ≤ 5 days	**0.57**	**1.05**	**1.33**	**1.69**	**2.57**	**0.55**	**1.01**	**1.29**	**1.64**	**2.50**
	5 days to ≤ 6 days	**0.49**	**0.91**	**1.16**	**1.47**	**2.23**	**0.48**	**0.89**	**1.13**	**1.44**	**2.20**
	6 days to ≤ 7 days	**0.43**	**0.79**	**1.01**	**1.29**	**1.98**	**0.41**	**0.77**	**1.00**	**1.28**	**1.96**
	7 days to ≤ 8 days	**0.39**	**0.73**	**0.94**	**1.21**	**1.86**	**0.38**	**0.71**	**0.92**	**1.19**	**1.85**
	8 days to ≤ 9 days	0.35	0.69	0.89	1.14	1.77	0.35	0.69	0.89	1.14	1.77
	9 days to ≤ 10 days	**0.34**	**0.67**	**0.88**	**1.14**	**1.78**	**0.34**	**0.67**	**0.86**	**1.11**	**1.72**
	10 days to ≤ 11 days	0.32	0.66	0.87	1.12	1.76	0.32	0.66	0.87	1.12	1.76
	11 days to ≤ 12 days	0.32	0.66	0.87	1.13	1.76	0.32	0.66	0.87	1.13	1.76
	12 days to ≤ 13 days	0.33	0.68	0.89	1.15	1.80	0.33	0.68	0.89	1.15	1.80
	13 days to ≤ 14 days	0.32	0.68	0.91	1.18	1.86	0.32	0.68	0.91	1.18	1.86
C3-DC+C4-OH	0 to ≤ 1 day	**0.04**	**0.07**	**0.10**	**0.13**	**0.21**	**0.04**	**0.07**	**0.09**	**0.12**	**0.19**
	1 day to ≤ 2 days	**0.04**	**0.08**	**0.13**	**0.20**	**0.34**	**0.04**	**0.08**	**0.13**	**0.20**	**0.35**
	2 days to ≤ 3 days	**0.04**	**0.08**	**0.12**	**0.17**	**0.29**	**0.04**	**0.08**	**0.11**	**0.16**	**0.27**
	3 days to ≤ 4 days	**0.04**	**0.08**	**0.10**	**0.15**	**0.25**	**0.04**	**0.07**	**0.10**	**0.14**	**0.24**
	4 days to ≤ 5 days	**0.03**	**0.06**	**0.08**	**0.11**	**0.18**	**0.03**	**0.06**	**0.08**	**0.11**	**0.18**
	5 days to ≤ 6 days	**0.03**	**0.06**	**0.08**	**0.10**	**0.16**	**0.03**	**0.06**	**0.08**	**0.10**	**0.16**
	6 days to ≤ 7 days	0.03	0.06	0.07	0.10	0.16	0.03	0.06	0.07	0.10	0.16
	7 days to ≤ 8 days	**0.03**	**0.06**	**0.07**	**0.09**	**0.13**	**0.03**	**0.06**	**0.07**	**0.09**	**0.13**
	8 days to ≤ 9 days	0.03	0.05	0.07	0.09	0.14	0.03	0.05	0.07	0.09	0.14
	9 days to ≤ 10 days	0.03	0.05	0.07	0.09	0.14	0.03	0.05	0.07	0.09	0.14
	10 days to ≤ 11 days	**0.03**	**0.05**	**0.06**	**0.08**	**0.12**	**0.03**	**0.05**	**0.06**	**0.08**	**0.12**
	11 days to ≤ 12 days	0.03	0.05	0.06	0.08	0.12	0.03	0.05	0.06	0.08	0.12
	12 days to ≤ 13 days	0.03	0.05	0.06	0.08	0.12	0.03	0.05	0.06	0.08	0.12
	13 days to ≤ 14 days	0.03	0.05	0.06	0.07	0.10	0.03	0.05	0.06	0.07	0.10
C4	0 to ≤ 1 day	**0.10**	**0.17**	**0.20**	**0.24**	**0.33**	**0.11**	**0.17**	**0.21**	**0.25**	**0.34**
	1 day to ≤ 2 days	**0.06**	**0.14**	**0.18**	**0.23**	**0.34**	**0.06**	**0.15**	**0.19**	**0.24**	**0.35**
	2 days to ≤ 3 days	**0.09**	**0.16**	**0.20**	**0.24**	**0.35**	**0.09**	**0.17**	**0.21**	**0.26**	**0.38**
	3 days to ≤ 4 days	**0.10**	**0.16**	**0.20**	**0.24**	**0.33**	**0.10**	**0.17**	**0.21**	**0.25**	**0.36**
	4 days to ≤ 5 days	**0.09**	**0.15**	**0.18**	**0.22**	**0.31**	**0.09**	**0.16**	**0.19**	**0.23**	**0.32**
	5 days to ≤ 6 days	**0.09**	**0.15**	**0.18**	**0.21**	**0.29**	**0.09**	**0.15**	**0.19**	**0.22**	**0.31**

	6 days to ≤ 7 days	**0.09**	**0.14**	**0.17**	**0.21**	**0.29**	**0.09**	**0.15**	**0.18**	**0.22**	**0.31**
	7 days to ≤ 8 days	**0.08**	**0.14**	**0.17**	**0.20**	**0.28**	**0.08**	**0.15**	**0.18**	**0.21**	**0.29**
	8 days to ≤ 9 days	**0.08**	**0.14**	**0.17**	**0.20**	**0.28**	**0.08**	**0.14**	**0.17**	**0.21**	**0.29**
	9 days to ≤ 10 days	**0.08**	**0.14**	**0.17**	**0.20**	**0.28**	**0.08**	**0.14**	**0.17**	**0.20**	**0.28**
	10 days to ≤ 11 days	**0.07**	**0.13**	**0.16**	**0.19**	**0.27**	**0.08**	**0.14**	**0.17**	**0.20**	**0.28**
	11 days to ≤ 12 days	**0.08**	**0.13**	**0.16**	**0.19**	**0.27**	**0.08**	**0.14**	**0.17**	**0.20**	**0.28**
	12 days to ≤ 13 days	**0.08**	**0.13**	**0.16**	**0.19**	**0.27**	**0.08**	**0.14**	**0.17**	**0.20**	**0.28**
	13 days to ≤ 14 days	**0.08**	**0.13**	**0.16**	**0.19**	**0.27**	**0.08**	**0.14**	**0.16**	**0.20**	**0.28**
C4-DC+C5-OH	0 to ≤ 1 day	**0.10**	**0.15**	**0.18**	**0.22**	**0.31**	**0.09**	**0.15**	**0.17**	**0.21**	**0.29**
	1 day to ≤ 2 days	**0.06**	**0.12**	**0.16**	**0.21**	**0.32**	**0.06**	**0.12**	**0.16**	**0.20**	**0.30**
	2 days to ≤ 3 days	**0.08**	**0.16**	**0.20**	**0.24**	**0.35**	**0.08**	**0.15**	**0.18**	**0.22**	**0.31**
	3 days to ≤ 4 days	**0.10**	**0.16**	**0.20**	**0.24**	**0.34**	**0.10**	**0.15**	**0.19**	**0.22**	**0.31**
	4 days to ≤ 5 days	**0.10**	**0.16**	**0.19**	**0.22**	**0.31**	**0.09**	**0.15**	**0.18**	**0.21**	**0.29**
	5 days to ≤ 6 days	**0.09**	**0.15**	**0.18**	**0.22**	**0.31**	**0.09**	**0.15**	**0.18**	**0.21**	**0.29**
	6 days to ≤ 7 days	**0.09**	**0.15**	**0.18**	**0.22**	**0.31**	**0.09**	**0.14**	**0.17**	**0.21**	**0.30**
	7 days to ≤ 8 days	**0.09**	**0.15**	**0.18**	**0.21**	**0.29**	**0.09**	**0.14**	**0.17**	**0.20**	**0.28**
	8 days to ≤ 9 days	**0.09**	**0.15**	**0.18**	**0.21**	**0.29**	**0.09**	**0.14**	**0.17**	**0.20**	**0.28**
	9 days to ≤ 10 days	**0.09**	**0.15**	**0.18**	**0.21**	**0.29**	**0.09**	**0.14**	**0.17**	**0.20**	**0.28**
	10 days to ≤ 11 days	**0.09**	**0.15**	**0.17**	**0.21**	**0.29**	**0.09**	**0.14**	**0.17**	**0.20**	**0.28**
	11 days to ≤ 12 days	**0.09**	**0.15**	**0.17**	**0.21**	**0.29**	**0.09**	**0.14**	**0.17**	**0.20**	**0.28**
	12 days to ≤ 13 days	**0.09**	**0.15**	**0.18**	**0.21**	**0.29**	**0.09**	**0.14**	**0.17**	**0.20**	**0.28**
	13 days to ≤ 14 days	**0.09**	**0.15**	**0.17**	**0.21**	**0.29**	**0.09**	**0.14**	**0.17**	**0.20**	**0.28**
C5	0 to ≤ 1 day	**0.04**	**0.06**	**0.08**	**0.10**	**0.16**	**0.04**	**0.07**	**0.09**	**0.11**	**0.16**
	1 day to ≤ 2 days	**0.04**	**0.04**	**0.08**	**0.11**	**0.19**	**0.00**	**0.04**	**0.08**	**0.11**	**0.20**
	2 days to ≤ 3 days	**0.04**	**0.08**	**0.10**	**0.12**	**0.18**	**0.01**	**0.08**	**0.10**	**0.13**	**0.19**
	3 days to ≤ 4 days	**0.05**	**0.08**	**0.10**	**0.12**	**0.18**	**0.05**	**0.09**	**0.11**	**0.13**	**0.19**
	4 days to ≤ 5 days	**0.05**	**0.08**	**0.10**	**0.13**	**0.19**	**0.05**	**0.09**	**0.11**	**0.14**	**0.21**
	5 days to ≤ 6 days	**0.05**	**0.09**	**0.11**	**0.14**	**0.21**	**0.06**	**0.09**	**0.12**	**0.15**	**0.22**
	6 days to ≤ 7 days	**0.05**	**0.09**	**0.12**	**0.15**	**0.23**	**0.06**	**0.10**	**0.12**	**0.16**	**0.24**
	7 days to ≤ 8 days	**0.05**	**0.10**	**0.12**	**0.16**	**0.24**	**0.05**	**0.10**	**0.13**	**0.16**	**0.24**
	8 days to ≤ 9 days	**0.05**	**0.10**	**0.13**	**0.16**	**0.25**	**0.05**	**0.10**	**0.13**	**0.17**	**0.26**
	9 days to ≤ 10 days	**0.03**	**0.10**	**0.13**	**0.17**	**0.26**	**0.05**	**0.11**	**0.13**	**0.17**	**0.26**

	10 days to ≤ 11 days	**0.04**	**0.10**	**0.13**	**0.17**	**0.26**	**0.05**	**0.11**	**0.14**	**0.18**	**0.27**
	11 days to ≤ 12 days	**0.04**	**0.11**	**0.14**	**0.18**	**0.27**	**0.05**	**0.11**	**0.14**	**0.18**	**0.27**
	12 days to ≤ 13 days	**0.05**	**0.11**	**0.14**	**0.18**	**0.27**	**0.05**	**0.11**	**0.14**	**0.18**	**0.28**
	13 days to ≤ 14 days	**0.04**	**0.11**	**0.14**	**0.18**	**0.27**	**0.05**	**0.11**	**0.15**	**0.19**	**0.30**
C5-DC+C6-OH	0 to ≤ 1 day	**0.05**	**0.09**	**0.11**	**0.14**	**0.20**	**0.05**	**0.09**	**0.11**	**0.13**	**0.19**
	1 day to ≤ 2 days	**0.05**	**0.10**	**0.13**	**0.17**	**0.26**	**0.05**	**0.10**	**0.13**	**0.17**	**0.26**
	2 days to ≤ 3 days	**0.05**	**0.09**	**0.11**	**0.14**	**0.21**	**0.05**	**0.09**	**0.11**	**0.14**	**0.21**
	3 days to ≤ 4 days	**0.05**	**0.08**	**0.10**	**0.13**	**0.19**	**0.05**	**0.08**	**0.10**	**0.13**	**0.19**
	4 days to ≤ 5 days	**0.04**	**0.07**	**0.09**	**0.11**	**0.16**	**0.04**	**0.07**	**0.09**	**0.11**	**0.16**
	5 days to ≤ 6 days	**0.04**	**0.07**	**0.08**	**0.10**	**0.14**	**0.04**	**0.07**	**0.08**	**0.10**	**0.14**
	6 days to ≤ 7 days	**0.04**	**0.07**	**0.08**	**0.10**	**0.14**	**0.04**	**0.06**	**0.08**	**0.10**	**0.14**
	7 days to ≤ 8 days	**0.04**	**0.06**	**0.08**	**0.10**	**0.15**	**0.04**	**0.06**	**0.08**	**0.09**	**0.15**
	8 days to ≤ 9 days	**0.04**	**0.06**	**0.07**	**0.09**	**0.13**	**0.04**	**0.06**	**0.07**	**0.09**	**0.13**
	9 days to ≤ 10 days	**0.04**	**0.06**	**0.07**	**0.09**	**0.13**	**0.04**	**0.06**	**0.07**	**0.09**	**0.13**
	10 days to ≤ 11 days	**0.04**	**0.06**	**0.07**	**0.09**	**0.13**	**0.04**	**0.06**	**0.07**	**0.09**	**0.13**
	11 days to ≤ 12 days	**0.03**	**0.06**	**0.07**	**0.09**	**0.13**	**0.04**	**0.06**	**0.07**	**0.09**	**0.13**
	12 days to ≤ 13 days	**0.04**	**0.06**	**0.07**	**0.09**	**0.13**	**0.03**	**0.06**	**0.07**	**0.09**	**0.13**
	13 days to ≤ 14 days	0.03	0.06	0.07	0.09	0.13	0.03	0.06	0.07	0.09	0.13
C6	0 to ≤ 1 day	0.02	0.04	0.05	0.06	0.09	0.03	0.04	0.04	0.05	0.06
	1 day to ≤ 2 days	0.02	0.04	0.05	0.06	0.09	0.02	0.04	0.05	0.06	0.09
	2 days to ≤ 3 days	0.02	0.03	0.04	0.05	0.08	0.02	0.03	0.04	0.05	0.08
	3 days to ≤ 4 days	0.02	0.03	0.04	0.05	0.08	0.01	0.03	0.04	0.05	0.08
	4 days to ≤ 5 days	0.02	0.03	0.03	0.04	0.05	0.02	0.03	0.03	0.04	0.05
	5 days to ≤ 6 days	0.02	0.03	0.03	0.04	0.05	0.01	0.02	0.03	0.04	0.06
	6 days to ≤ 7 days	**0.01**	**0.02**	**0.03**	**0.04**	**0.06**	**0.01**	**0.02**	**0.03**	**0.04**	**0.06**
	7 days to ≤ 8 days	**0.01**	**0.02**	**0.03**	**0.04**	**0.06**	**0.01**	**0.02**	**0.03**	**0.04**	**0.07**
	8 days to ≤ 9 days	**0.01**	**0.02**	**0.03**	**0.04**	**0.06**	**0.01**	**0.02**	**0.03**	**0.04**	**0.06**
	9 days to ≤ 10 days	0.02	0.03	0.03	0.04	0.05	0.01	0.02	0.03	0.04	0.06
	10 days to ≤ 11 days	0.02	0.03	0.03	0.04	0.05	0.02	0.03	0.03	0.04	0.05
	11 days to ≤ 12 days	0.02	0.03	0.03	0.04	0.05	0.02	0.03	0.03	0.04	0.05
	12 days to ≤ 13 days	0.02	0.03	0.03	0.04	0.05	0.02	0.03	0.03	0.04	0.05
	13 days to ≤ 14 days	0.02	0.03	0.03	0.04	0.05	0.02	0.03	0.03	0.04	0.05

C6-DC	0 to ≤ 1 day	**0.03**	**0.07**	**0.09**	**0.11**	**0.16**	**0.03**	**0.07**	**0.09**	**0.12**	**0.19**
	1 day to ≤ 2 days	**0.04**	**0.08**	**0.11**	**0.15**	**0.24**	**0.04**	**0.08**	**0.11**	**0.15**	**0.25**
	2 days to ≤ 3 days	**0.03**	**0.07**	**0.09**	**0.12**	**0.19**	**0.03**	**0.07**	**0.09**	**0.13**	**0.21**
	3 days to ≤ 4 days	**0.03**	**0.06**	**0.08**	**0.12**	**0.20**	**0.03**	**0.06**	**0.09**	**0.12**	**0.20**
	4 days to ≤ 5 days	**0.03**	**0.05**	**0.07**	**0.10**	**0.17**	**0.03**	**0.05**	**0.07**	**0.10**	**0.17**
	5 days to ≤ 6 days	**0.03**	**0.05**	**0.07**	**0.09**	**0.15**	**0.03**	**0.05**	**0.07**	**0.09**	**0.15**
	6 days to ≤ 7 days	**0.03**	**0.05**	**0.06**	**0.09**	**0.15**	**0.03**	**0.05**	**0.06**	**0.09**	**0.15**
	7 days to ≤ 8 days	**0.03**	**0.05**	**0.06**	**0.08**	**0.14**	**0.03**	**0.05**	**0.06**	**0.09**	**0.15**
	8 days to ≤ 9 days	0.03	0.05	0.06	0.09	0.15	0.03	0.05	0.06	0.09	0.15
	9 days to ≤ 10 days	0.03	0.05	0.07	0.09	0.15	0.03	0.05	0.07	0.09	0.15
	10 days to ≤ 11 days	0.03	0.05	0.07	0.09	0.15	0.03	0.05	0.07	0.09	0.15
	11 days to ≤ 12 days	0.03	0.05	0.07	0.09	0.15	0.03	0.05	0.07	0.09	0.15
	12 days to ≤ 13 days	0.03	0.05	0.07	0.09	0.15	0.03	0.05	0.07	0.09	0.15
	13 days to ≤ 14 days	0.03	0.05	0.07	0.09	0.15	0.03	0.05	0.07	0.09	0.15
C8	0 to ≤ 1 day	**0.02**	**0.04**	**0.05**	**0.07**	**0.11**	**0.02**	**0.04**	**0.05**	**0.06**	**0.09**
	1 day to ≤ 2 days	**0.02**	**0.04**	**0.06**	**0.07**	**0.11**	**0.02**	**0.04**	**0.05**	**0.07**	**0.11**
	2 days to ≤ 3 days	**0.02**	**0.04**	**0.06**	**0.07**	**0.11**	**0.02**	**0.04**	**0.05**	**0.07**	**0.11**
	3 days to ≤ 4 days	**0.02**	**0.04**	**0.05**	**0.07**	**0.11**	**0.02**	**0.04**	**0.05**	**0.06**	**0.09**
	4 days to ≤ 5 days	**0.02**	**0.03**	**0.04**	**0.05**	**0.08**	**0.02**	**0.03**	**0.04**	**0.05**	**0.08**
	5 days to ≤ 6 days	**0.02**	**0.03**	**0.04**	**0.05**	**0.08**	**0.02**	**0.03**	**0.04**	**0.05**	**0.08**
	6 days to ≤ 7 days	**0.02**	**0.03**	**0.04**	**0.05**	**0.08**	**0.02**	**0.03**	**0.04**	**0.05**	**0.08**
	7 days to ≤ 8 days	**0.02**	**0.03**	**0.04**	**0.05**	**0.08**	**0.02**	**0.03**	**0.04**	**0.05**	**0.08**
	8 days to ≤ 9 days	**0.02**	**0.03**	**0.04**	**0.05**	**0.08**	**0.02**	**0.03**	**0.04**	**0.05**	**0.08**
	9 days to ≤ 10 days	**0.02**	**0.03**	**0.04**	**0.05**	**0.08**	**0.02**	**0.03**	**0.04**	**0.05**	**0.08**
	10 days to ≤ 11 days	**0.02**	**0.03**	**0.04**	**0.05**	**0.08**	**0.02**	**0.03**	**0.04**	**0.05**	**0.08**
	11 days to ≤ 12 days	**0.02**	**0.03**	**0.04**	**0.05**	**0.08**	**0.02**	**0.03**	**0.04**	**0.05**	**0.08**
	12 days to ≤ 13 days	**0.02**	**0.03**	**0.04**	**0.05**	**0.08**	**0.02**	**0.03**	**0.04**	**0.05**	**0.08**
	13 days to ≤ 14 days	**0.02**	**0.03**	**0.04**	**0.05**	**0.08**	**0.02**	**0.03**	**0.04**	**0.05**	**0.08**
C8:1	0 to ≤ 1 day	**0.04**	**0.08**	**0.11**	**0.14**	**0.22**	**0.04**	**0.08**	**0.11**	**0.14**	**0.22**
	1 day to ≤ 2 days	**0.04**	**0.09**	**0.12**	**0.16**	**0.25**	**0.04**	**0.08**	**0.12**	**0.15**	**0.24**
	2 days to ≤ 3 days	**0.05**	**0.09**	**0.12**	**0.16**	**0.24**	**0.05**	**0.09**	**0.12**	**0.16**	**0.25**
	3 days to ≤ 4 days	**0.05**	**0.10**	**0.13**	**0.17**	**0.26**	**0.05**	**0.10**	**0.13**	**0.16**	**0.24**

	4 days to ≤ 5 days	**0.04**	**0.09**	**0.12**	**0.15**	**0.24**	**0.04**	**0.09**	**0.12**	**0.15**	**0.23**
	5 days to ≤ 6 days	0.03	0.08	0.11	0.15	0.24	0.03	0.08	0.11	0.15	0.24
	6 days to ≤ 7 days	0.03	0.08	0.11	0.15	0.24	0.03	0.08	0.11	0.15	0.24
	7 days to ≤ 8 days	0.03	0.08	0.11	0.15	0.24	0.03	0.08	0.11	0.15	0.24
	8 days to ≤ 9 days	0.03	0.08	0.11	0.15	0.25	0.03	0.08	0.11	0.15	0.25
	9 days to ≤ 10 days	**0.03**	**0.08**	**0.12**	**0.16**	**0.27**	**0.03**	**0.08**	**0.12**	**0.16**	**0.27**
	10 days to ≤ 11 days	0.03	0.08	0.12	0.16	0.27	0.03	0.08	0.12	0.16	0.27
	11 days to ≤ 12 days	**0.03**	**0.08**	**0.12**	**0.16**	**0.27**	**0.03**	**0.08**	**0.12**	**0.17**	**0.28**
	12 days to ≤ 13 days	0.03	0.09	0.12	0.17	0.28	0.03	0.09	0.12	0.17	0.28
	13 days to ≤ 14 days	0.03	0.09	0.12	0.17	0.28	0.03	0.09	0.12	0.17	0.28
C10	0 to ≤ 1 day	**0.03**	**0.06**	**0.08**	**0.10**	**0.16**	**0.03**	**0.06**	**0.07**	**0.09**	**0.13**
	1 day to ≤ 2 days	**0.03**	**0.06**	**0.08**	**0.10**	**0.16**	**0.03**	**0.06**	**0.07**	**0.10**	**0.16**
	2 days to ≤ 3 days	**0.03**	**0.06**	**0.07**	**0.10**	**0.16**	**0.03**	**0.05**	**0.07**	**0.09**	**0.15**
	3 days to ≤ 4 days	**0.03**	**0.05**	**0.07**	**0.09**	**0.15**	**0.03**	**0.05**	**0.07**	**0.09**	**0.15**
	4 days to ≤ 5 days	**0.02**	**0.04**	**0.06**	**0.07**	**0.11**	**0.02**	**0.04**	**0.05**	**0.07**	**0.11**
	5 days to ≤ 6 days	**0.02**	**0.04**	**0.05**	**0.07**	**0.11**	**0.02**	**0.04**	**0.05**	**0.06**	**0.09**
	6 days to ≤ 7 days	**0.02**	**0.04**	**0.05**	**0.06**	**0.09**	**0.02**	**0.04**	**0.05**	**0.06**	**0.09**
	7 days to ≤ 8 days	**0.02**	**0.04**	**0.05**	**0.06**	**0.09**	**0.02**	**0.04**	**0.05**	**0.06**	**0.09**
	8 days to ≤ 9 days	**0.02**	**0.04**	**0.05**	**0.06**	**0.09**	**0.02**	**0.04**	**0.05**	**0.06**	**0.09**
	9 days to ≤ 10 days	**0.02**	**0.04**	**0.05**	**0.06**	**0.09**	**0.02**	**0.04**	**0.05**	**0.06**	**0.09**
	10 days to ≤ 11 days	**0.02**	**0.04**	**0.05**	**0.06**	**0.09**	**0.02**	**0.04**	**0.05**	**0.06**	**0.09**
	11 days to ≤ 12 days	**0.02**	**0.04**	**0.05**	**0.06**	**0.09**	**0.02**	**0.04**	**0.05**	**0.06**	**0.09**
	12 days to ≤ 13 days	**0.02**	**0.04**	**0.05**	**0.06**	**0.09**	**0.02**	**0.04**	**0.05**	**0.06**	**0.09**
	13 days to ≤ 14 days	**0.02**	**0.04**	**0.05**	**0.06**	**0.09**	**0.02**	**0.04**	**0.05**	**0.06**	**0.09**
C10:1	0 to ≤ 1 day	**0.03**	**0.05**	**0.07**	**0.08**	**0.12**	**0.03**	**0.05**	**0.06**	**0.08**	**0.12**
	1 day to ≤ 2 days	**0.03**	**0.05**	**0.07**	**0.09**	**0.14**	**0.03**	**0.05**	**0.07**	**0.09**	**0.15**
	2 days to ≤ 3 days	**0.03**	**0.06**	**0.07**	**0.09**	**0.13**	**0.03**	**0.05**	**0.07**	**0.09**	**0.14**
	3 days to ≤ 4 days	**0.03**	**0.06**	**0.07**	**0.10**	**0.15**	**0.03**	**0.05**	**0.07**	**0.09**	**0.15**
	4 days to ≤ 5 days	**0.02**	**0.05**	**0.07**	**0.09**	**0.14**	**0.02**	**0.05**	**0.06**	**0.08**	**0.12**
	5 days to ≤ 6 days	**0.02**	**0.05**	**0.06**	**0.08**	**0.12**	**0.02**	**0.04**	**0.06**	**0.08**	**0.13**
	6 days to ≤ 7 days	**0.02**	**0.04**	**0.06**	**0.08**	**0.13**	**0.02**	**0.04**	**0.06**	**0.07**	**0.11**
	7 days to ≤ 8 days	**0.02**	**0.04**	**0.06**	**0.07**	**0.11**	**0.02**	**0.04**	**0.05**	**0.07**	**0.11**

	8 days to ≤ 9 days	**0.02**	**0.04**	**0.06**	**0.07**	**0.11**	**0.02**	**0.04**	**0.05**	**0.07**	**0.11**
	9 days to ≤ 10 days	**0.02**	**0.04**	**0.06**	**0.07**	**0.11**	**0.02**	**0.04**	**0.05**	**0.07**	**0.11**
	10 days to ≤ 11 days	**0.02**	**0.04**	**0.06**	**0.07**	**0.11**	**0.02**	**0.04**	**0.06**	**0.07**	**0.11**
	11 days to ≤ 12 days	**0.02**	**0.04**	**0.06**	**0.07**	**0.11**	**0.02**	**0.04**	**0.06**	**0.07**	**0.11**
	12 days to ≤ 13 days	**0.02**	**0.04**	**0.06**	**0.07**	**0.11**	**0.02**	**0.04**	**0.06**	**0.07**	**0.11**
	13 days to ≤ 14 days	0.02	0.04	0.06	0.07	0.11	0.02	0.04	0.06	0.07	0.11
C12	0 to ≤ 1 day	**0.04**	**0.07**	**0.10**	**0.13**	**0.21**	**0.03**	**0.07**	**0.09**	**0.11**	**0.16**
	1 day to ≤ 2 days	**0.03**	**0.07**	**0.09**	**0.12**	**0.19**	**0.03**	**0.06**	**0.08**	**0.11**	**0.18**
	2 days to ≤ 3 days	**0.03**	**0.06**	**0.08**	**0.11**	**0.18**	**0.03**	**0.06**	**0.08**	**0.10**	**0.16**
	3 days to ≤ 4 days	**0.03**	**0.05**	**0.07**	**0.09**	**0.13**	**0.03**	**0.05**	**0.07**	**0.09**	**0.15**
	4 days to ≤ 5 days	**0.02**	**0.04**	**0.06**	**0.07**	**0.11**	**0.02**	**0.04**	**0.05**	**0.07**	**0.11**
	5 days to ≤ 6 days	**0.02**	**0.04**	**0.05**	**0.06**	**0.10**	**0.02**	**0.04**	**0.05**	**0.06**	**0.09**
	6 days to ≤ 7 days	**0.02**	**0.04**	**0.05**	**0.06**	**0.09**	**0.02**	**0.04**	**0.05**	**0.06**	**0.09**
	7 days to ≤ 8 days	**0.02**	**0.04**	**0.05**	**0.06**	**0.09**	**0.02**	**0.04**	**0.05**	**0.06**	**0.09**
	8 days to ≤ 9 days	**0.02**	**0.04**	**0.05**	**0.06**	**0.09**	**0.02**	**0.04**	**0.05**	**0.06**	**0.09**
	9 days to ≤ 10 days	**0.02**	**0.04**	**0.05**	**0.06**	**0.09**	**0.02**	**0.04**	**0.05**	**0.06**	**0.09**
	10 days to ≤ 11 days	**0.02**	**0.04**	**0.05**	**0.06**	**0.09**	**0.02**	**0.04**	**0.05**	**0.06**	**0.09**
	11 days to ≤ 12 days	**0.02**	**0.04**	**0.05**	**0.06**	**0.09**	**0.02**	**0.04**	**0.04**	**0.05**	**0.08**
	12 days to ≤ 13 days	**0.02**	**0.04**	**0.05**	**0.06**	**0.09**	**0.03**	**0.04**	**0.04**	**0.05**	**0.06**
	13 days to ≤ 14 days	**0.02**	**0.04**	**0.04**	**0.05**	**0.08**	**0.03**	**0.04**	**0.04**	**0.05**	**0.06**
C12:1	0 to ≤ 1 day	**0.02**	**0.05**	**0.09**	**0.13**	**0.23**	**0.02**	**0.05**	**0.09**	**0.12**	**0.20**
	1 day to ≤ 2 days	**0.02**	**0.04**	**0.06**	**0.10**	**0.18**	**0.02**	**0.04**	**0.06**	**0.09**	**0.16**
	2 days to ≤ 3 days	**0.02**	**0.04**	**0.06**	**0.09**	**0.16**	**0.02**	**0.04**	**0.06**	**0.08**	**0.14**
	3 days to ≤ 4 days	**0.01**	**0.04**	**0.05**	**0.07**	**0.11**	**0.01**	**0.03**	**0.05**	**0.07**	**0.11**
	4 days to ≤ 5 days	**0.01**	**0.03**	**0.04**	**0.05**	**0.08**	**0.01**	**0.03**	**0.04**	**0.05**	**0.08**
	5 days to ≤ 6 days	**0.01**	**0.02**	**0.03**	**0.05**	**0.08**	**0.01**	**0.02**	**0.03**	**0.05**	**0.08**
	6 days to ≤ 7 days	**0.01**	**0.02**	**0.03**	**0.05**	**0.09**	**0.01**	**0.02**	**0.03**	**0.04**	**0.07**
	7 days to ≤ 8 days	**0.01**	**0.02**	**0.03**	**0.04**	**0.07**	**0.01**	**0.02**	**0.03**	**0.04**	**0.07**
	8 days to ≤ 9 days	**0.01**	**0.02**	**0.03**	**0.04**	**0.07**	**0.01**	**0.02**	**0.03**	**0.04**	**0.07**
	9 days to ≤ 10 days	**0.01**	**0.02**	**0.03**	**0.04**	**0.07**	**0.01**	**0.02**	**0.03**	**0.04**	**0.07**
	10 days to ≤ 11 days	0.01	0.02	0.03	0.04	0.07	0.01	0.02	0.03	0.04	0.07
	11 days to ≤ 12 days	**0.01**	**0.02**	**0.03**	**0.04**	**0.07**	**0.01**	**0.02**	**0.03**	**0.04**	**0.07**

	12 days to ≤ 13 days	0.01	0.02	0.03	0.04	0.07	0.01	0.02	0.03	0.04	0.07
	13 days to ≤ 14 days	0.01	0.02	0.03	0.04	0.07	0.01	0.02	0.03	0.04	0.07
C14	0 to ≤ 1 day	**0.09**	**0.16**	**0.19**	**0.23**	**0.32**	**0.08**	**0.14**	**0.17**	**0.21**	**0.30**
	1 day to ≤ 2 days	**0.09**	**0.15**	**0.19**	**0.23**	**0.34**	**0.08**	**0.14**	**0.17**	**0.21**	**0.30**
	2 days to ≤ 3 days	**0.09**	**0.15**	**0.19**	**0.23**	**0.34**	**0.08**	**0.14**	**0.17**	**0.20**	**0.29**
	3 days to ≤ 4 days	**0.08**	**0.14**	**0.18**	**0.22**	**0.32**	**0.08**	**0.13**	**0.16**	**0.19**	**0.27**
	4 days to ≤ 5 days	**0.07**	**0.13**	**0.16**	**0.19**	**0.27**	**0.06**	**0.11**	**0.14**	**0.17**	**0.25**
	5 days to ≤ 6 days	**0.07**	**0.12**	**0.15**	**0.19**	**0.28**	**0.06**	**0.11**	**0.13**	**0.17**	**0.25**
	6 days to ≤ 7 days	**0.06**	**0.11**	**0.14**	**0.18**	**0.27**	**0.06**	**0.10**	**0.13**	**0.16**	**0.24**
	7 days to ≤ 8 days	**0.06**	**0.11**	**0.13**	**0.17**	**0.25**	**0.05**	**0.10**	**0.12**	**0.15**	**0.22**
	8 days to ≤ 9 days	**0.05**	**0.10**	**0.13**	**0.16**	**0.24**	**0.05**	**0.09**	**0.11**	**0.14**	**0.21**
	9 days to ≤ 10 days	**0.05**	**0.09**	**0.12**	**0.15**	**0.23**	**0.05**	**0.09**	**0.11**	**0.14**	**0.20**
	10 days to ≤ 11 days	**0.05**	**0.09**	**0.11**	**0.14**	**0.21**	**0.04**	**0.08**	**0.10**	**0.13**	**0.20**
	11 days to ≤ 12 days	**0.04**	**0.08**	**0.10**	**0.13**	**0.20**	**0.04**	**0.08**	**0.10**	**0.12**	**0.18**
	12 days to ≤ 13 days	**0.04**	**0.08**	**0.10**	**0.12**	**0.18**	**0.04**	**0.07**	**0.09**	**0.11**	**0.17**
	13 days to ≤ 14 days	**0.04**	**0.07**	**0.09**	**0.12**	**0.19**	**0.04**	**0.07**	**0.09**	**0.11**	**0.16**
C14:1	0 to ≤ 1 day	**0.04**	**0.08**	**0.10**	**0.13**	**0.20**	**0.04**	**0.08**	**0.10**	**0.13**	**0.19**
	1 day to ≤ 2 days	**0.04**	**0.07**	**0.09**	**0.12**	**0.21**	**0.04**	**0.07**	**0.09**	**0.12**	**0.19**
	2 days to ≤ 3 days	**0.04**	**0.06**	**0.08**	**0.11**	**0.16**	**0.03**	**0.06**	**0.08**	**0.11**	**0.18**
	3 days to ≤ 4 days	**0.03**	**0.06**	**0.07**	**0.10**	**0.16**	**0.03**	**0.05**	**0.07**	**0.09**	**0.13**
	4 days to ≤ 5 days	**0.03**	**0.04**	**0.06**	**0.07**	**0.10**	**0.03**	**0.04**	**0.05**	**0.07**	**0.11**
	5 days to ≤ 6 days	**0.02**	**0.04**	**0.05**	**0.06**	**0.09**	**0.02**	**0.04**	**0.05**	**0.06**	**0.09**
	6 days to ≤ 7 days	**0.02**	**0.04**	**0.05**	**0.06**	**0.09**	**0.02**	**0.04**	**0.05**	**0.06**	**0.09**
	7 days to ≤ 8 days	**0.02**	**0.04**	**0.05**	**0.06**	**0.09**	**0.02**	**0.04**	**0.05**	**0.06**	**0.09**
	8 days to ≤ 9 days	**0.02**	**0.04**	**0.05**	**0.06**	**0.09**	**0.02**	**0.04**	**0.04**	**0.05**	**0.08**
	9 days to ≤ 10 days	**0.02**	**0.04**	**0.04**	**0.06**	**0.09**	**0.02**	**0.03**	**0.04**	**0.05**	**0.06**
	10 days to ≤ 11 days	**0.03**	**0.04**	**0.04**	**0.05**	**0.06**	**0.02**	**0.03**	**0.04**	**0.05**	**0.06**
	11 days to ≤ 12 days	**0.03**	**0.04**	**0.04**	**0.05**	**0.06**	**0.02**	**0.03**	**0.04**	**0.05**	**0.07**
	12 days to ≤ 13 days	**0.02**	**0.03**	**0.04**	**0.05**	**0.06**	**0.02**	**0.03**	**0.04**	**0.05**	**0.07**
	13 days to ≤ 14 days	0.02	0.03	0.04	0.05	0.07	0.02	0.03	0.04	0.05	0.07
C16	0 to ≤ 1 day	**1.29**	**2.48**	**3.05**	**3.68**	**5.25**	**1.26**	**2.36**	**2.89**	**3.48**	**4.98**
	1 day to ≤ 2 days	**1.26**	**2.45**	**3.10**	**3.83**	**5.60**	**1.18**	**2.29**	**2.89**	**3.57**	**5.24**

	2 days to ≤ 3 days	**1.22**	**2.46**	**3.14**	**3.92**	**5.82**	**1.14**	**2.29**	**2.92**	**3.65**	**5.43**
	3 days to ≤ 4 days	**1.12**	**2.27**	**2.93**	**3.70**	**5.58**	**1.05**	**2.12**	**2.74**	**3.46**	**5.22**
	4 days to ≤ 5 days	**0.93**	**1.84**	**2.39**	**3.09**	**4.75**	**0.87**	**1.72**	**2.24**	**2.88**	**4.43**
	5 days to ≤ 6 days	**0.82**	**1.61**	**2.06**	**2.64**	**4.07**	**0.78**	**1.51**	**1.93**	**2.47**	**3.80**
	6 days to ≤ 7 days	**0.74**	**1.42**	**1.81**	**2.30**	**3.51**	**0.68**	**1.32**	**1.69**	**2.13**	**3.25**
	7 days to ≤ 8 days	**0.66**	**1.27**	**1.62**	**2.05**	**3.13**	**0.61**	**1.18**	**1.50**	**1.90**	**2.90**
	8 days to ≤ 9 days	**0.59**	**1.16**	**1.48**	**1.87**	**2.84**	**0.57**	**1.08**	**1.36**	**1.72**	**2.63**
	9 days to ≤ 10 days	**0.55**	**1.06**	**1.35**	**1.70**	**2.61**	**0.52**	**0.98**	**1.24**	**1.56**	**2.38**
	10 days to ≤ 11 days	**0.50**	**0.97**	**1.22**	**1.54**	**2.34**	**0.49**	**0.90**	**1.13**	**1.43**	**2.17**
	11 days to ≤ 12 days	**0.47**	**0.89**	**1.12**	**1.41**	**2.14**	**0.46**	**0.83**	**1.04**	**1.30**	**1.95**
	12 days to ≤ 13 days	**0.44**	**0.82**	**1.03**	**1.30**	**1.97**	**0.42**	**0.77**	**0.96**	**1.20**	**1.80**
	13 days to ≤ 14 days	**0.42**	**0.77**	**0.96**	**1.20**	**1.79**	**0.39**	**0.72**	**0.90**	**1.11**	**1.65**
C16:1	0 to ≤ 1 day	**0.06**	**0.15**	**0.20**	**0.25**	**0.38**	**0.06**	**0.14**	**0.19**	**0.24**	**0.35**
	1 day to ≤ 2 days	**0.06**	**0.14**	**0.19**	**0.25**	**0.39**	**0.06**	**0.13**	**0.18**	**0.23**	**0.36**
	2 days to ≤ 3 days	**0.06**	**0.13**	**0.18**	**0.24**	**0.38**	**0.05**	**0.12**	**0.17**	**0.22**	**0.35**
	3 days to ≤ 4 days	**0.05**	**0.11**	**0.16**	**0.21**	**0.34**	**0.05**	**0.11**	**0.15**	**0.20**	**0.32**
	4 days to ≤ 5 days	**0.04**	**0.08**	**0.11**	**0.16**	**0.27**	**0.04**	**0.07**	**0.10**	**0.14**	**0.23**
	5 days to ≤ 6 days	**0.03**	**0.07**	**0.09**	**0.12**	**0.19**	**0.03**	**0.06**	**0.08**	**0.11**	**0.18**
	6 days to ≤ 7 days	**0.03**	**0.06**	**0.08**	**0.10**	**0.16**	**0.03**	**0.05**	**0.07**	**0.09**	**0.15**
	7 days to ≤ 8 days	**0.03**	**0.05**	**0.07**	**0.09**	**0.15**	**0.03**	**0.05**	**0.06**	**0.08**	**0.12**
	8 days to ≤ 9 days	**0.03**	**0.05**	**0.06**	**0.08**	**0.12**	**0.02**	**0.04**	**0.06**	**0.07**	**0.11**
	9 days to ≤ 10 days	**0.02**	**0.04**	**0.06**	**0.07**	**0.11**	**0.02**	**0.04**	**0.05**	**0.07**	**0.11**
	10 days to ≤ 11 days	**0.02**	**0.04**	**0.05**	**0.07**	**0.11**	**0.02**	**0.04**	**0.05**	**0.06**	**0.09**
	11 days to ≤ 12 days	**0.02**	**0.04**	**0.05**	**0.06**	**0.09**	**0.02**	**0.04**	**0.04**	**0.06**	**0.09**
	12 days to ≤ 13 days	**0.02**	**0.04**	**0.04**	**0.06**	**0.09**	**0.02**	**0.03**	**0.04**	**0.05**	**0.08**
	13 days to ≤ 14 days	**0.02**	**0.03**	**0.04**	**0.05**	**0.08**	**0.02**	**0.03**	**0.04**	**0.05**	**0.08**
C16:1-OH	0 to ≤ 1 day	0.02	0.03	0.04	0.04	0.05	0.02	0.03	0.03	0.04	0.05
	1 day to ≤ 2 days	**0.02**	**0.03**	**0.04**	**0.05**	**0.07**	**0.02**	**0.03**	**0.03**	**0.04**	**0.05**
	2 days to ≤ 3 days	0.02	0.03	0.03	0.04	0.05	0.02	0.03	0.03	0.04	0.05
	3 days to ≤ 4 days	0.02	0.03	0.03	0.04	0.05	0.02	0.03	0.03	0.04	0.05
	4 days to ≤ 5 days	**0.02**	**0.03**	**0.03**	**0.04**	**0.05**	**0.01**	**0.02**	**0.03**	**0.04**	**0.06**
	5 days to ≤ 6 days	**0.02**	**0.03**	**0.03**	**0.04**	**0.05**	**0.01**	**0.02**	**0.03**	**0.04**	**0.06**

	6 days to ≤ 7 days	**0.01**	0.02	0.03	0.04	0.06	0.01	0.02	0.03	0.04	0.06
	7 days to ≤ 8 days	**0.01**	**0.02**	**0.03**	**0.04**	**0.06**	**0.01**	**0.02**	**0.03**	**0.03**	**0.04**
	8 days to ≤ 9 days	0.01	0.02	0.03	0.03	0.04	0.01	0.02	0.03	0.03	0.04
	9 days to ≤ 10 days	0.01	0.02	0.03	0.03	0.04	0.01	0.02	0.02	0.03	0.04
	10 days to ≤ 11 days	0.01	0.02	0.02	0.03	0.04	0.01	0.02	0.02	0.03	0.04
	11 days to ≤ 12 days	0.01	0.02	0.02	0.03	0.04	0.01	0.02	0.02	0.03	0.04
	12 days to ≤ 13 days	0.01	0.02	0.02	0.03	0.04	0.01	0.02	0.02	0.03	0.04
	13 days to ≤ 14 days	0.01	0.02	0.02	0.03	0.04	0.01	0.02	0.02	0.03	0.04
C18	0 to ≤ 1 day	**0.41**	**0.69**	**0.83**	**1.00**	**1.40**	**0.40**	**0.68**	**0.81**	**0.97**	**1.37**
	1 day to ≤ 2 days	**0.41**	**0.70**	**0.85**	**1.03**	**1.49**	**0.40**	**0.67**	**0.82**	**1.00**	**1.44**
	2 days to ≤ 3 days	**0.40**	**0.70**	**0.86**	**1.05**	**1.51**	**0.39**	**0.68**	**0.83**	**1.02**	**1.47**
	3 days to ≤ 4 days	**0.38**	**0.66**	**0.82**	**1.00**	**1.46**	**0.37**	**0.64**	**0.80**	**0.98**	**1.41**
	4 days to ≤ 5 days	**0.33**	**0.58**	**0.72**	**0.89**	**1.30**	**0.32**	**0.57**	**0.70**	**0.87**	**1.27**
	5 days to ≤ 6 days	**0.30**	**0.53**	**0.67**	**0.83**	**1.22**	**0.30**	**0.53**	**0.66**	**0.81**	**1.18**
	6 days to ≤ 7 days	**0.28**	**0.49**	**0.62**	**0.77**	**1.14**	**0.27**	**0.49**	**0.61**	**0.75**	**1.11**
	7 days to ≤ 8 days	**0.26**	**0.46**	**0.58**	**0.72**	**1.06**	**0.26**	**0.45**	**0.57**	**0.70**	**1.02**
	8 days to ≤ 9 days	**0.24**	**0.44**	**0.55**	**0.68**	**1.00**	**0.24**	**0.44**	**0.54**	**0.67**	**0.99**
	9 days to ≤ 10 days	**0.24**	**0.42**	**0.52**	**0.65**	**0.96**	**0.23**	**0.41**	**0.51**	**0.64**	**0.96**
	10 days to ≤ 11 days	0.22	0.39	0.49	0.61	0.92	0.22	0.39	0.49	0.61	0.92
	11 days to ≤ 12 days	0.21	0.37	0.47	0.58	0.87	0.21	0.37	0.47	0.58	0.87
	12 days to ≤ 13 days	0.20	0.36	0.45	0.56	0.83	0.20	0.36	0.45	0.56	0.83
	13 days to ≤ 14 days	0.19	0.34	0.43	0.53	0.81	0.19	0.34	0.43	0.53	0.81
C18:1	0 to ≤ 1 day	**0.61**	**1.03**	**1.24**	**1.50**	**2.12**	**0.61**	**0.98**	**1.18**	**1.42**	**1.99**
	1 day to ≤ 2 days	**0.70**	**1.15**	**1.37**	**1.64**	**2.30**	**0.65**	**1.07**	**1.29**	**1.54**	**2.16**
	2 days to ≤ 3 days	**0.74**	**1.21**	**1.46**	**1.75**	**2.46**	**0.69**	**1.13**	**1.36**	**1.63**	**2.29**
	3 days to ≤ 4 days	**0.75**	**1.22**	**1.47**	**1.76**	**2.47**	**0.70**	**1.14**	**1.37**	**1.64**	**2.31**
	4 days to ≤ 5 days	**0.69**	**1.15**	**1.39**	**1.68**	**2.37**	**0.64**	**1.06**	**1.29**	**1.55**	**2.20**
	5 days to ≤ 6 days	**0.65**	**1.09**	**1.32**	**1.60**	**2.28**	**0.60**	**1.01**	**1.22**	**1.48**	**2.11**
	6 days to ≤ 7 days	**0.61**	**1.01**	**1.23**	**1.50**	**2.15**	**0.56**	**0.93**	**1.14**	**1.38**	**1.97**
	7 days to ≤ 8 days	**0.55**	**0.94**	**1.16**	**1.41**	**2.03**	**0.52**	**0.87**	**1.06**	**1.30**	**1.86**
	8 days to ≤ 9 days	**0.51**	**0.87**	**1.07**	**1.31**	**1.90**	**0.47**	**0.81**	**0.99**	**1.20**	**1.74**
	9 days to ≤ 10 days	**0.47**	**0.80**	**1.00**	**1.23**	**1.81**	**0.43**	**0.74**	**0.92**	**1.13**	**1.64**

	10 days to ≤ 11 days	**0.42**	**0.74**	**0.91**	**1.12**	**1.65**	**0.40**	**0.69**	**0.85**	**1.06**	**1.55**
	11 days to ≤ 12 days	**0.38**	**0.68**	**0.85**	**1.06**	**1.55**	**0.37**	**0.64**	**0.79**	**0.98**	**1.44**
	12 days to ≤ 13 days	**0.38**	**0.65**	**0.81**	**1.00**	**1.45**	**0.36**	**0.61**	**0.75**	**0.92**	**1.36**
	13 days to ≤ 14 days	**0.36**	**0.62**	**0.76**	**0.94**	**1.38**	**0.33**	**0.58**	**0.71**	**0.88**	**1.28**
C18:2	0 to ≤ 1 day	**0.06**	**0.12**	**0.16**	**0.23**	**0.38**	**0.06**	**0.10**	**0.14**	**0.21**	**0.36**
	1 day to ≤ 2 days	**0.07**	**0.14**	**0.19**	**0.27**	**0.44**	**0.06**	**0.12**	**0.17**	**0.24**	**0.40**
	2 days to ≤ 3 days	**0.07**	**0.15**	**0.21**	**0.28**	**0.45**	**0.06**	**0.13**	**0.18**	**0.26**	**0.43**
	3 days to ≤ 4 days	**0.08**	**0.18**	**0.24**	**0.33**	**0.53**	**0.07**	**0.15**	**0.22**	**0.29**	**0.47**
	4 days to ≤ 5 days	**0.10**	**0.23**	**0.31**	**0.40**	**0.62**	**0.08**	**0.20**	**0.28**	**0.36**	**0.55**
	5 days to ≤ 6 days	**0.11**	**0.26**	**0.34**	**0.43**	**0.65**	**0.09**	**0.23**	**0.30**	**0.38**	**0.58**
	6 days to ≤ 7 days	**0.11**	**0.26**	**0.33**	**0.42**	**0.63**	**0.10**	**0.23**	**0.30**	**0.38**	**0.58**
	7 days to ≤ 8 days	**0.11**	**0.25**	**0.32**	**0.40**	**0.60**	**0.10**	**0.22**	**0.29**	**0.36**	**0.55**
	8 days to ≤ 9 days	**0.11**	**0.23**	**0.30**	**0.38**	**0.58**	**0.09**	**0.21**	**0.28**	**0.35**	**0.53**
	9 days to ≤ 10 days	**0.10**	**0.22**	**0.29**	**0.37**	**0.57**	**0.09**	**0.20**	**0.26**	**0.33**	**0.51**
	10 days to ≤ 11 days	**0.10**	**0.21**	**0.27**	**0.35**	**0.53**	**0.09**	**0.19**	**0.25**	**0.32**	**0.49**
	11 days to ≤ 12 days	**0.10**	**0.21**	**0.27**	**0.34**	**0.51**	**0.09**	**0.19**	**0.25**	**0.32**	**0.49**
	12 days to ≤ 13 days	**0.10**	**0.20**	**0.26**	**0.33**	**0.50**	**0.09**	**0.18**	**0.24**	**0.30**	**0.45**
	13 days to ≤ 14 days	**0.09**	**0.20**	**0.25**	**0.32**	**0.48**	**0.08**	**0.18**	**0.23**	**0.30**	**0.46**

[a] Sex specific partitions are shown in bold. If no statistical difference was found between males and females within the age partitions, data were combined and percentiles re-estimated.

[b] ALA, alanine; ARG, arginine; CIT, citrulline; GLY, glycine; LEU, leucine; ILE, isoleucine; ALLO-ILE, alloisoleucine; PRO-OH, hydroxyproline; MET, methionine; ORN, ornithine; PHE, phenylalanine; PRO, proline; TYR, Tyrosine; VAL, valine; C0, free carnitine; C2, acetylcarnitine; C3, propionylcarnitine; C3-DC+C4-OH, malonylcarnitine+3-hydroxybutyrylcarnitine; C4, butyrylcarnitine+isobutyrylcarnitine; C4-DC+C5-OH, methylmalonylcarnitine+3-hydroxyisovalerylcarnitine; C5, isovalerylcarnitine+methylbutyrylcarnitine; C5-DC+C6-OH, glutarylcarnitine+3-hydroxyhexanoylcarnitine; C6, hexanoylcarnitine; C6-DC, methylglutarylcarnitine; C8, octanoylcarnitine; C8:1, octenoylcarnitine; C10, decanoylcarnitine; C10:1, decenoylcarnitine; C12, dodecanoylcarnitine; C12:1, dodecenoylcarnitine; C14, tetradecanoylcarnitine; C14:1, tetradecenoylcarnitine; C16, palmitoylcarnitine; C16:1, palmitoleylcarnitine; C16:1-OH, 3-hydroxypalmitoleylcarnitine; C18, stearoylcarnitine; C18:1, oleoylcarnitine; C18:2, linoleoylcarnitine.

Table S11. Reference intervals partitioned by Harris and Boyd approach for 35 MS/MS NBS biomarkers (µM)

		Amino acids			
		Male		Female	
Analyte	Age	Lower limit (2.5th) & 90% CI	Upper limit (97.5th) & 90% CI	Lower limit (2.5th) & 90% CI	Upper limit (97.5th) & 90% CI
ALA	0 to ≤ 1 day	**171.0 (168.7-172.9)**	**404.2 (401.7-406.4)**	**171.8 (169.9-173.7)**	**406.7 (404.3-409.2)**
	1 day to ≤ 2 days	165.8 (165.5-166.3)	442.3 (441.8-442.9)	165.8 (165.5-166.3)	442.3 (441.8-442.9)
	2 days to ≤ 3 days	181.0 (180.9-181.2)	503.1 (502.8-503.3)	181.0 (180.9-181.2)	503.1 (502.8-503.3)
	3 days to ≤ 4 days	191.6 (191.4-191.8)	536.2 (535.8-536.5)	191.6 (191.4-191.8)	536.2 (535.8-536.5)
	4 days to ≤ 5 days	192.1 (191.6-192.5)	529.5 (528.8-530.2)	192.1 (191.6-192.5)	529.5 (528.8-530.2)
	5 days to ≤ 6 days	191.8 (191.3-192.3)	517.4 (516.7-518.4)	191.8 (191.3-192.3)	517.4 (516.7-518.4)
	6 days to ≤ 7 days	189.1 (188.5-189.8)	503.6 (502.6-504.7)	189.1 (188.5-189.8)	503.6 (502.6-504.7)
	7 days to ≤ 8 days	**182.3 (180.8-183.6)**	**488.0 (485.7-489.8)**	**190.4 (189.0-192.0)**	**503.6 (501.7-505.8)**
	8 days to ≤ 10 days	183.2 (182.3-184.1)	488.1 (486.9-489.2)	183.2 (182.3-184.1)	488.1 (486.9-489.2)
	10 days to ≤ 12 days	**178.5 (177.1-179.7)**	**469.9 (468.2-472.0)**	**188.4 (186.9-189.7)**	**484.3 (481.8-486.1)**
	12 days to ≤ 14 days	180.5 (179.2-181.6)	467.5 (466.5-468.9)	180.5 (179.2-181.6)	467.5 (466.5-468.9)
ARG	0 to ≤ 14 days	1.6 (1.6-1.6)	26.9 (26.9-26.9)	1.6 (1.6-1.6)	26.9 (26.9-26.9)
CIT	0 to ≤ 5 days	7.8 (7.8-7.8)	21.5 (21.5-21.6)	7.8 (7.8-7.8)	21.5 (21.5-21.6)
	5 days to ≤ 6 days	7.8 (7.8-7.8)	21.0 (21.0-21.0)	7.8 (7.8-7.8)	21.0 (21.0-21.0)
	6 days to ≤ 8 days	7.7 (7.7-7.8)	21.6 (21.6-21.7)	7.7 (7.7-7.8)	21.6 (21.6-21.7)
	8 days to ≤ 14 days	8.2 (8.2-8.3)	24.2 (24.2-24.3)	8.2 (8.2-8.3)	24.2 (24.2-24.3)
GLY	0 to ≤ 1 day	**310.4 (307.0-313.3)**	**682.4 (679.2-685.0)**	**317.1 (313.0-320.1)**	**691.6 (688.4-695.0)**
	1 day to ≤ 2 days	**289.6 (288.4-290.6)**	**738.1 (736.7-739.3)**	**301.7 (300.8-302.7)**	**753.6 (752.0-755.1)**
	2 days to ≤ 3 days	**287.1 (286.8-287.4)**	**766.7 (766.2-767.2)**	**299.4 (299.0-299.7)**	**777.6 (777.0-778.2)**
	3 days to ≤ 4 days	**273.0 (272.7-273.4)**	**737.1 (736.6-737.6)**	**286.3 (285.9-286.6)**	**751.5 (751.0-752.1)**
	4 days to ≤ 5 days	**238.1 (237.4-239.0)**	**630.8 (629.7-631.8)**	**250.0 (249.3-250.6)**	**646.3 (645.2-647.6)**
	5 days to ≤ 7 days	**218.5 (218.0-219.1)**	**569.9 (569.0-570.8)**	**228.0 (227.3-228.6)**	**583.1 (582.1-584.2)**
	7 days to ≤ 8 days	**211.0 (210.0-212.0)**	**538.8 (537.2-540.9)**	**218.4 (217.3-219.9)**	**554.7 (552.8-557.5)**
	8 days to ≤ 9 days	**207.5 (205.3-209.1)**	**524.5 (522.5-526.7)**	**216.7 (215.3-218.4)**	**536.4 (534.3-539.2)**
	9 days to ≤ 10 days	**207.8 (206.3-209.5)**	**513.4 (511.6-516.3)**	**213.7 (211.8-215.4)**	**526.2 (523.5-528.0)**

	10 days to ≤ 11 days	**204.1 (202.2-206.2)**	**506.5 (504.0-509.1)**	**212.6 (210.8-214.1)**	**515.2 (513.2-518.4)**
	11 days to ≤ 12 days	**201.0 (199.2-202.3)**	**487.7 (483.8-490.6)**	**206.3 (203.9-208.1)**	**494.8 (492.2-497.2)**
	12 days to ≤ 13 days	**201.9 (200.5-203.8)**	**484.2 (481.8-486.3)**	**207.9 (206.3-210.9)**	**494.4 (491.0-497.8)**
	13 days to ≤ 14 days	**198.7 (197.5-200.8)**	**479.3 (476.8-481.5)**	**204.7 (203.3-206.4)**	**482.0 (478.8-485.0)**
LEU/ILE/ALLO-ILE/PRO-OH	0 to ≤ 1 day	**61.5 (61.1-62.4)**	**195.1 (192.9-196.6)**	**60.8 (60.2-61.5)**	**195.0 (193.0-197.0)**
	1 day to ≤ 2 days	**76.0 (75.6-76.2)**	**210.5 (210.1-210.9)**	**76.8 (76.5-77.1)**	**214.8 (214.4-215.4)**
	2 days to ≤ 4 days	95.5 (95.4-95.5)	239.8 (239.7-239.9)	95.5 (95.4-95.5)	239.8 (239.7-239.9)
	4 days to ≤ 6 days	99.3 (99.1-99.6)	264.5 (264.2-264.7)	99.3 (99.1-99.6)	264.5 (264.2-264.7)
	6 days to ≤ 8 days	100.3 (99.9-100.6)	277.2 (276.8-277.7)	100.3 (99.9-100.6)	277.2 (276.8-277.7)
	8 days to ≤ 9 days	102.0 (101.5-102.9)	280.7 (279.6-281.6)	102.0 (101.5-102.9)	280.7 (279.6-281.6)
	9 days to ≤ 10 days	**100.4 (99.7-101.3)**	**277.3 (276.2-278.5)**	**104.9 (103.9-105.9)**	**282.7 (281.2-284.0)**
	10 days to ≤ 11 days	**101.3 (100.4-102.5)**	**276.6 (274.7-278.0)**	**105.1 (103.7-105.9)**	**284.2 (283.0-285.5)**
	11 days to ≤ 12 days	102.9 (102.2-103.6)	277.7 (276.5-278.9)	102.9 (102.2-103.6)	277.7 (276.5-278.9)
	12 days to ≤ 14 days	103.1 (102.4-103.7)	278.5 (277.6-279.2)	103.1 (102.4-103.7)	278.5 (277.6-279.2)
MET	0 to ≤ 4 days	9.3 (9.3-9.3)	31.6 (31.5-31.6)	9.3 (9.3-9.3)	31.6 (31.5-31.6)
	4 days to ≤ 5 days	8.9 (8.9-9.0)	31.1 (31.1-31.2)	8.9 (8.9-9.0)	31.1 (31.1-31.2)
	5 days to ≤ 14 days	9.0 (9.0-9.0)	31.6 (31.6-31.7)	9.0 (9.0-9.0)	31.6 (31.6-31.7)
ORN	0 to ≤ 1 day	**42.9 (42.4-43.2)**	**149.6 (147.9-150.3)**	**43.3 (42.9-43.9)**	**145.0 (143.8-145.9)**
	1 day to ≤ 2 days	**50.0 (49.9-50.3)**	**169.7 (169.3-170.2)**	**49.6 (49.4-49.8)**	**169.0 (168.6-169.4)**
	2 days to ≤ 3 days	**61.1 (61.0-61.2)**	**205.3 (205.2-205.5)**	**61.7 (61.7-61.8)**	**202.0 (201.9-202.2)**
	3 days to ≤ 4 days	**67.2 (67.1-67.3)**	**219.4 (219.2-219.5)**	**68.5 (68.4-68.6)**	**218.1 (217.9-218.3)**
	4 days to ≤ 7 days	65.2 (65.1-65.3)	208.9 (208.7-209.1)	65.2 (65.1-65.3)	208.9 (208.7-209.1)
	7 days to ≤ 8 days	**61.4 (60.7-61.9)**	**198.0 (197.2-199.0)**	**61.9 (61.4-62.5)**	**196.2 (195.4-197.0)**
	8 days to ≤ 9 days	**60.5 (59.6-61.1)**	**196.8 (195.8-197.8)**	**62.9 (62.2-63.4)**	**196.5 (195.4-197.3)**
	9 days to ≤ 10 days	**62.3 (61.6-63.1)**	**196.1 (194.9-196.9)**	**63.9 (63.4-64.6)**	**195.5 (194.6-196.7)**
	10 days to ≤ 11 days	63.8 (63.4-64.4)	198.6 (197.6-199.3)	63.8 (63.4-64.4)	198.6 (197.6-199.3)
	11 days to ≤ 14 days	65.7 (65.3-66.0)	199.2 (198.7-199.7)	65.7 (65.3-66.0)	199.2 (198.7-199.7)
PHE	0 to ≤ 1 day	37.0 (36.7-37.2)	74.5 (74.3-74.9)	37.0 (36.7-37.2)	74.5 (74.3-74.9)
	1 day to ≤ 2 days	**34.7 (34.6-34.8)**	**74.2 (74.1-74.3)**	**34.8 (34.6-34.9)**	**74.2 (74.0-74.3)**
	2 days to ≤ 4 days	35.2 (35.1-35.2)	78.4 (78.4-78.5)	35.2 (35.1-35.2)	78.4 (78.4-78.5)
	4 days to ≤ 5 days	32.0 (32.0-32.1)	73.4 (73.3-73.5)	32.0 (32.0-32.1)	73.4 (73.3-73.5)
	5 days to ≤ 7 days	31.0 (30.9-31.0)	72.4 (72.3-72.5)	31.0 (30.9-31.0)	72.4 (72.3-72.5)

	7 days to ≤ 9 days	**29.8 (29.6-29.9)**	**71.3 (71.1-71.5)**	**30.1 (30.0-30.3)**	**72.2 (72.0-72.4)**
	9 days to ≤ 12 days	29.7 (29.6-29.8)	70.3 (70.2-70.5)	29.7 (29.6-29.8)	70.3 (70.2-70.5)
	12 days to ≤ 13 days	**29.3 (29.0-29.6)**	**68.1 (67.8-68.5)**	**30.1 (29.8-30.4)**	**70.1 (69.7-70.5)**
	13 days to ≤ 14 days	**29.2 (28.7-29.4)**	**67.7 (67.3-68.0)**	**30.3 (29.9-30.5)**	**68.7 (68.3-69.1)**
PRO	0 to ≤ 1 day	**109.2 (108.3-110.2)**	**237.0 (235.6-238.6)**	**107.4 (106.6-108.4)**	**231.7 (230.2-233.2)**
	1 day to ≤ 3 days	**118.8 (118.7-119.0)**	**292.3 (292.1-292.5)**	**120.6 (120.5-120.7)**	**293.2 (293.0-293.4)**
	3 days to ≤ 5 days	**124.3 (124.1-124.4)**	**310.4 (310.2-310.6)**	**126.5 (126.4-126.7)**	**312.9 (312.7-313.1)**
	5 days to ≤ 6 days	**118.5 (117.9-119.0)**	**316.5 (315.8-317.1)**	**121.4 (120.9-121.9)**	**320.6 (319.9-321.4)**
	6 days to ≤ 8 days	119.0 (118.6-119.3)	316.7 (316.2-317.2)	119.0 (118.6-119.3)	316.7 (316.2-317.2)
	8 days to ≤ 9 days	118.0 (117.5-118.6)	310.4 (309.5-311.3)	118.0 (117.5-118.6)	310.4 (309.5-311.3)
	9 days to ≤ 10 days	118.4 (117.8-119.2)	308.1 (307.1-309.2)	118.4 (117.8-119.2)	308.1 (307.1-309.2)
	10 days to ≤ 11 days	**116.7 (115.6-117.8)**	**302.1 (300.2-303.5)**	**120.5 (118.7-121.5)**	**305.7 (303.8-307.4)**
	11 days to ≤ 12 days	**117.9 (116.8-119.4)**	**300.2 (298.6-301.6)**	**121.3 (120.2-122.4)**	**303.4 (301.7-304.9)**
	12 days to ≤ 13 days	**117.8 (116.8-118.8)**	**299.1 (297.2-300.8)**	**120.6 (119.7-121.6)**	**298.6 (297.4-300.0)**
	13 days to ≤ 14 days	**117.6 (116.2-119.6)**	**295.9 (294.2-297.8)**	**120.0 (118.4-121.7)**	**295.7 (293.0-297.9)**
TYR	0 to ≤ 1 day	45.2 (44.8-45.5)	151.7 (151.0-152.4)	45.2 (44.8-45.5)	151.7 (151.0-152.4)
	1 day to ≤ 4 days	50.9 (50.9-51.0)	182.7 (182.7-182.8)	50.9 (50.9-51.0)	182.7 (182.7-182.8)
	4 days to ≤ 5 days	51.3 (51.2-51.5)	194.3 (194.0-194.6)	51.3 (51.2-51.5)	194.3 (194.0-194.6)
	5 days to ≤ 6 days	49.5 (49.2-49.6)	191.9 (191.6-192.3)	49.5 (49.2-49.6)	191.9 (191.6-192.3)
	6 days to ≤ 7 days	**46.5 (46.1-46.8)**	**181.5 (181.0-182.1)**	**48.8 (48.5-49.2)**	**188.0 (187.4-188.5)**
	7 days to ≤ 12 days	**45.1 (44.8-45.3)**	**165.0 (164.6-165.4)**	**48.0 (47.7-48.3)**	**172.8 (172.4-173.4)**
	12 days to ≤ 14 days	47.4 (47.0-47.9)	159.0 (158.5-159.8)	47.4 (47.0-47.9)	159.0 (158.5-159.8)
VAL	0 to ≤ 1 day	**56.3 (55.8-56.7)**	**162.1 (161.2-163.3)**	**55.9 (55.4-56.5)**	**159.7 (158.6-161.0)**
	1 day to ≤ 2 days	**64.9 (64.7-65.2)**	**179.1 (178.7-179.4)**	**66.4 (66.1-66.6)**	**184.9 (184.4-185.3)**
	2 days to ≤ 3 days	**79.5 (79.4-79.6)**	**204.4 (204.3-204.6)**	**82.4 (82.2-82.5)**	**211.2 (211.1-211.4)**
	3 days to ≤ 6 days	88.9 (88.8-89.0)	219.4 (219.3-219.5)	88.9 (88.8-89.0)	219.4 (219.3-219.5)
	6 days to ≤ 8 days	85.9 (85.6-86.1)	229.6 (229.3-230.0)	85.9 (85.6-86.1)	229.6 (229.3-230.0)
	8 days to ≤ 9 days	85.3 (84.9-85.7)	230.0 (229.4-230.8)	85.3 (84.9-85.7)	230.0 (229.4-230.8)
	9 days to ≤ 11 days	**83.2 (82.7-83.8)**	**224.2 (223.5-225.0)**	**88.4 (87.7-89.2)**	**234.0 (233.4-234.9)**
	11 days to ≤ 12 days	**83.1 (82.2-84.2)**	**220.9 (219.7-222.7)**	**88.5 (87.5-89.6)**	**232.0 (230.7-233.0)**
	12 days to ≤ 14 days	85.3 (84.7-85.8)	226.8 (226.1-227.6)	85.3 (84.7-85.8)	226.8 (226.1-227.6)
			**Acylcarnitines**		

C0	0 to ≤ 3 days	**11.16 (11.15-11.18)**	**37.45 (37.43-37.48)**	**10.53 (10.52-10.54)**	**34.42 (34.39-34.44)**
	3 days to ≤ 11 days	11.47 (11.46-11.48)	37.46 (37.44-37.48)	11.47 (11.46-11.48)	37.46 (37.44-37.48)
	11 days to ≤ 12 days	**12.35 (12.17-12.48)**	**41.02 (40.74-41.22)**	**12.15 (11.96-12.31)**	**39.69 (39.32-39.97)**
	12 days to ≤ 14 days	12.76 (12.63-12.87)	40.95 (40.79-41.11)	12.76 (12.63-12.87)	40.95 (40.79-41.11)
C2	0 to ≤ 7 days	7.95 (7.94-7.96)	30.67 (30.65-30.68)	7.95 (7.94-7.96)	30.67 (30.65-30.68)
	7 days to ≤ 14 days	4.98 (4.96-5.00)	18.45 (18.41-18.48)	4.98 (4.96-5.00)	18.45 (18.41-18.48)
C3	0 to ≤ 14 days	0.64 (0.64-0.64)	2.79 (2.79-2.80)	0.64 (0.64-0.64)	2.79 (2.79-2.80)
C3-DC+C4-OH	0 to ≤ 1 day	0.04 (0.04-0.04)	0.19 (0.19-0.20)	0.04 (0.04-0.04)	0.19 (0.19-0.20)
	1 day to ≤ 5 days	0.04 (0.04-0.04)	0.25 (0.25-0.25)	0.04 (0.04-0.04)	0.25 (0.25-0.25)
	5 days to ≤ 7 days	0.04 (0.04-0.04)	0.15 (0.15-0.15)	0.04 (0.04-0.04)	0.15 (0.15-0.15)
	7 days to ≤ 8 days	0.04 (0.04-0.04)	0.13 (0.13-0.14)	0.04 (0.04-0.04)	0.13 (0.13-0.14)
	8 days to ≤ 9 days	0.03 (0.03-0.03)	0.13 (0.13-0.13)	0.03 (0.03-0.03)	0.13 (0.13-0.13)
	9 days to ≤ 10 days	0.03 (0.03-0.03)	0.13 (0.13-0.13)	0.03 (0.03-0.03)	0.13 (0.13-0.13)
	10 days to ≤ 11 days	**0.03 (0.03-0.03)**	**0.11 (0.11-0.11)**	**0.03 (0.03-0.03)**	**0.11 (0.10-0.11)**
	11 days to ≤ 13 days	0.03 (0.03-0.03)	0.11 (0.11-0.11)	0.03 (0.03-0.03)	0.11 (0.11-0.11)
	13 days to ≤ 14 days	0.03 (0.03-0.03)	0.10 (0.10-0.10)	0.03 (0.03-0.03)	0.10 (0.10-0.10)
C4	0 to ≤ 1 day	0.12 (0.12-0.12)	0.32 (0.32-0.32)	0.12 (0.12-0.12)	0.32 (0.32-0.32)
	1 day to ≤ 2 days	0.07 (0.07-0.07)	0.33 (0.33-0.34)	0.07 (0.07-0.07)	0.33 (0.33-0.34)
	2 days to ≤ 3 days	0.11 (0.11-0.11)	0.34 (0.34-0.34)	0.11 (0.11-0.11)	0.34 (0.34-0.34)
	3 days to ≤ 7 days	0.11 (0.11-0.11)	0.32 (0.32-0.32)	0.11 (0.11-0.11)	0.32 (0.32-0.32)
	7 days to ≤ 11 days	**0.09 (0.09-0.09)**	**0.27 (0.27-0.27)**	**0.09 (0.09-0.10)**	**0.28 (0.28-0.28)**
	11 days to ≤ 14 days	0.09 (0.09-0.09)	0.26 (0.26-0.26)	0.09 (0.09-0.09)	0.26 (0.26-0.26)
C4-DC+C5-OH	0 to ≤ 1 day	0.10 (0.10-0.10)	0.29 (0.29-0.29)	0.10 (0.10-0.10)	0.29 (0.29-0.29)
	1 day to ≤ 3 days	0.09 (0.09-0.09)	0.31 (0.31-0.31)	0.09 (0.09-0.09)	0.31 (0.31-0.31)
	3 days to ≤ 6 days	0.11 (0.11-0.11)	0.31 (0.31-0.31)	0.11 (0.11-0.11)	0.31 (0.31-0.31)
	6 days to ≤ 7 days	**0.10 (0.10-0.10)**	**0.29 (0.29-0.29)**	**0.10 (0.10-0.10)**	**0.28 (0.28-0.28)**
	7 days to ≤ 14 days	**0.10 (0.10-0.10)**	**0.28 (0.28-0.28)**	**0.10 (0.10-0.10)**	**0.27 (0.27-0.27)**
C5	0 to ≤ 1 day	**0.04 (0.04-0.04)**	**0.15 (0.15-0.15)**	**0.04 (0.03-0.04)**	**0.15 (0.15-0.15)**
	1 day to ≤ 2 days	0.01 (0.01-0.01)	0.18 (0.18-0.18)	0.01 (0.01-0.01)	0.18 (0.18-0.18)
	2 days to ≤ 3 days	**0.05 (0.05-0.05)**	**0.17 (0.17-0.17)**	**0.04 (0.04-0.04)**	**0.18 (0.18-0.18)**
	3 days to ≤ 4 days	**0.06 (0.06-0.06)**	**0.17 (0.17-0.17)**	**0.06 (0.06-0.06)**	**0.18 (0.18-0.18)**
	4 days to ≤ 5 days	0.06 (0.06-0.06)	0.19 (0.19-0.19)	0.06 (0.06-0.06)	0.19 (0.19-0.19)

	5 days to ≤ 6 days	**0.06 (0.06-0.06)**	**0.20 (0.20-0.20)**	**0.06 (0.06-0.06)**	**0.21 (0.21-0.21)**
	6 days to ≤ 8 days	**0.06 (0.06-0.06)**	**0.22 (0.22-0.23)**	**0.06 (0.06-0.06)**	**0.23 (0.23-0.23)**
	8 days to ≤ 9 days	**0.06 (0.06-0.06)**	**0.23 (0.23-0.23)**	**0.06 (0.06-0.06)**	**0.25 (0.25-0.25)**
	9 days to ≤ 10 days	**0.06 (0.06-0.06)**	**0.25 (0.25-0.25)**	**0.06 (0.05-0.06)**	**0.25 (0.25-0.26)**
	10 days to ≤ 14 days	0.06 (0.06-0.06)	0.26 (0.26-0.26)	0.06 (0.06-0.06)	0.26 (0.26-0.26)
C5-DC+C6-OH	0 to ≤ 2 days	0.06 (0.06-0.06)	0.24 (0.24-0.24)	0.06 (0.06-0.06)	0.24 (0.24-0.24)
	2 days to ≤ 3 days	0.06 (0.06-0.06)	0.20 (0.20-0.20)	0.06 (0.06-0.06)	0.20 (0.20-0.20)
	3 days to ≤ 5 days	0.05 (0.05-0.05)	0.18 (0.18-0.18)	0.05 (0.05-0.05)	0.18 (0.18-0.18)
	5 days to ≤ 6 days	**0.05 (0.05-0.05)**	**0.14 (0.14-0.14)**	**0.05 (0.04-0.05)**	**0.13 (0.13-0.14)**
	6 days to ≤ 7 days	0.04 (0.04-0.04)	0.13 (0.13-0.13)	0.04 (0.04-0.04)	0.13 (0.13-0.13)
	7 days to ≤ 8 days	0.04 (0.04-0.04)	0.14 (0.14-0.14)	0.04 (0.04-0.04)	0.14 (0.14-0.14)
	8 days to ≤ 9 days	0.04 (0.04-0.04)	0.12 (0.12-0.12)	0.04 (0.04-0.04)	0.12 (0.12-0.12)
	9 days to ≤ 10 days	0.04 (0.04-0.04)	0.12 (0.12-0.12)	0.04 (0.04-0.04)	0.12 (0.12-0.12)
	10 days to ≤ 14 days	0.04 (0.04-0.04)	0.12 (0.12-0.12)	0.04 (0.04-0.04)	0.12 (0.12-0.12)
C6	0 to ≤ 1 day	**0.02 (0.02-0.02)**	**0.08 (0.08-0.08)**	**0.03 (0.03-0.03)**	**0.06 (0.06-0.06)**
	1 day to ≤ 2 days	**0.02 (0.02-0.02)**	**0.08 (0.08-0.08)**	**0.02 (0.02-0.02)**	**0.08 (0.08-0.08)**
	2 days to ≤ 3 days	**0.02 (0.02-0.02)**	**0.08 (0.08-0.08)**	**0.02 (0.02-0.02)**	**0.07 (0.07-0.07)**
	3 days to ≤ 6 days	0.02 (0.02-0.02)	0.07 (0.07-0.07)	0.02 (0.02-0.02)	0.07 (0.07-0.07)
	6 days to ≤ 9 days	0.01 (0.01-0.01)	0.06 (0.06-0.06)	0.01 (0.01-0.01)	0.06 (0.06-0.06)
	9 days to ≤ 10 days	**0.02 (0.02-0.02)**	**0.05 (0.05-0.05)**	**0.01 (0.01-0.01)**	**0.06 (0.06-0.06)**
	10 days to ≤ 11 days	**0.02 (0.02-0.02)**	**0.05 (0.05-0.05)**	**0.02 (0.02-0.02)**	**0.05 (0.05-0.05)**
	11 days to ≤ 14 days	0.02 (0.02-0.02)	0.05 (0.05-0.05)	0.02 (0.02-0.02)	0.05 (0.05-0.05)
C6-DC	0 to ≤ 1 day	**0.04 (0.04-0.04)**	**0.16 (0.16-0.17)**	**0.04 (0.04-0.04)**	**0.18 (0.18-0.19)**
	1 day to ≤ 3 days	**0.04 (0.04-0.04)**	**0.18 (0.18-0.18)**	**0.04 (0.04-0.04)**	**0.21 (0.21-0.21)**
	3 days to ≤ 4 days	0.04 (0.04-0.04)	0.19 (0.19-0.19)	0.04 (0.04-0.04)	0.19 (0.19-0.19)
	4 days to ≤ 10 days	0.03 (0.03-0.03)	0.15 (0.15-0.15)	0.03 (0.03-0.03)	0.15 (0.15-0.15)
	10 days to ≤ 14 days	0.03 (0.03-0.03)	0.14 (0.14-0.14)	0.03 (0.03-0.03)	0.14 (0.14-0.14)
C8	0 to ≤ 1 day	**0.03 (0.03-0.03)**	**0.10 (0.10-0.10)**	**0.03 (0.03-0.03)**	**0.08 (0.08-0.09)**
	1 day to ≤ 2 days	**0.03 (0.03-0.03)**	**0.10 (0.10-0.10)**	**0.03 (0.03-0.03)**	**0.10 (0.10-0.10)**
	2 days to ≤ 3 days	**0.03 (0.03-0.03)**	**0.10 (0.10-0.10)**	**0.03 (0.03-0.03)**	**0.10 (0.10-0.10)**
	3 days to ≤ 4 days	**0.03 (0.03-0.03)**	**0.10 (0.10-0.10)**	**0.02 (0.02-0.02)**	**0.09 (0.09-0.09)**
	4 days to ≤ 5 days	**0.02 (0.02-0.02)**	**0.08 (0.08-0.08)**	**0.02 (0.02-0.02)**	**0.08 (0.08-0.08)**

	5 days to ≤ 14 days	0.02 (0.02-0.02)	0.07 (0.07-0.07)	0.02 (0.02-0.02)	0.07 (0.07-0.07)
C8:1	0 to ≤ 3 days	0.05 (0.05-0.05)	0.23 (0.23-0.23)	0.05 (0.05-0.05)	0.23 (0.23-0.23)
	3 days to ≤ 14 days	0.05 (0.05-0.05)	0.24 (0.24-0.24)	0.05 (0.05-0.05)	0.24 (0.24-0.24)
C10	0 to ≤ 2 days	**0.03 (0.03-0.03)**	**0.15 (0.15-0.15)**	**0.03 (0.03-0.03)**	**0.15 (0.15-0.15)**
	2 days to ≤ 3 days	0.03 (0.03-0.03)	0.15 (0.15-0.15)	0.03 (0.03-0.03)	0.15 (0.15-0.15)
	3 days to ≤ 5 days	0.03 (0.03-0.03)	0.14 (0.14-0.14)	0.03 (0.03-0.03)	0.14 (0.14-0.14)
	5 days to ≤ 6 days	**0.03 (0.03-0.03)**	**0.10 (0.10-0.10)**	**0.02 (0.02-0.03)**	**0.09 (0.09-0.09)**
	6 days to ≤ 7 days	**0.03 (0.03-0.03)**	**0.09 (0.09-0.09)**	**0.02 (0.02-0.02)**	**0.09 (0.09-0.09)**
	7 days to ≤ 9 days	**0.03 (0.02-0.03)**	**0.09 (0.09-0.09)**	**0.02 (0.02-0.02)**	**0.09 (0.09-0.09)**
	9 days to ≤ 10 days	**0.03 (0.02-0.03)**	**0.09 (0.09-0.09)**	**0.02 (0.02-0.02)**	**0.09 (0.09-0.09)**
	10 days to ≤ 11 days	**0.03 (0.02-0.03)**	**0.09 (0.09-0.09)**	**0.03 (0.02-0.03)**	**0.09 (0.09-0.10)**
	11 days to ≤ 12 days	**0.02 (0.02-0.02)**	**0.09 (0.09-0.09)**	**0.02 (0.02-0.02)**	**0.09 (0.09-0.10)**
	12 days to ≤ 13 days	**0.03 (0.03-0.03)**	**0.09 (0.09-0.09)**	**0.02 (0.02-0.02)**	**0.09 (0.08-0.09)**
	13 days to ≤ 14 days	**0.03 (0.02-0.03)**	**0.09 (0.09-0.10)**	**0.03 (0.02-0.03)**	**0.09 (0.09-0.10)**
C10:1	0 to ≤ 1 day	**0.04 (0.04-0.05)**	**0.11 (0.11-0.11)**	**0.03 (0.03-0.03)**	**0.11 (0.11-0.11)**
	1 day to ≤ 3 days	0.03 (0.03-0.03)	0.13 (0.13-0.13)	0.03 (0.03-0.03)	0.13 (0.13-0.13)
	3 days to ≤ 6 days	0.03 (0.03-0.03)	0.14 (0.14-0.14)	0.03 (0.03-0.03)	0.14 (0.14-0.14)
	6 days to ≤ 7 days	**0.03 (0.03-0.03)**	**0.12 (0.12-0.12)**	**0.02 (0.02-0.02)**	**0.11 (0.11-0.12)**
	7 days to ≤ 12 days	0.02 (0.02-0.02)	0.10 (0.10-0.10)	0.02 (0.02-0.02)	0.10 (0.10-0.10)
	12 days to ≤ 14 days	0.03 (0.02-0.03)	0.10 (0.10-0.10)	0.03 (0.02-0.03)	0.10 (0.10-0.10)
C12	0 to ≤ 1 day	**0.04 (0.04-0.04)**	**0.19 (0.18-0.19)**	**0.04 (0.04-0.04)**	**0.15 (0.14-0.15)**
	1 day to ≤ 3 days	0.04 (0.04-0.04)	0.16 (0.16-0.16)	0.04 (0.04-0.04)	0.16 (0.16-0.16)
	3 days to ≤ 5 days	**0.03 (0.03-0.03)**	**0.13 (0.13-0.14)**	**0.03 (0.03-0.03)**	**0.14 (0.14-0.14)**
	5 days to ≤ 6 days	**0.03 (0.03-0.03)**	**0.09 (0.09-0.09)**	**0.03 (0.02-0.03)**	**0.09 (0.09-0.09)**
	6 days to ≤ 8 days	**0.02 (0.02-0.02)**	**0.09 (0.09-0.09)**	**0.02 (0.02-0.02)**	**0.08 (0.08-0.08)**
	8 days to ≤ 9 days	**0.02 (0.02-0.02)**	**0.08 (0.08-0.08)**	**0.02 (0.02-0.02)**	**0.08 (0.08-0.08)**
	9 days to ≤ 10 days	**0.02 (0.02-0.02)**	**0.08 (0.07-0.08)**	**0.02 (0.02-0.02)**	**0.08 (0.08-0.08)**
	10 days to ≤ 11 days	**0.02 (0.02-0.02)**	**0.08 (0.08-0.08)**	**0.02 (0.02-0.02)**	**0.08 (0.08-0.08)**
	11 days to ≤ 12 days	0.02 (0.02-0.02)	0.08 (0.08-0.08)	0.02 (0.02-0.02)	0.08 (0.08-0.08)
	12 days to ≤ 13 days	**0.02 (0.02-0.02)**	**0.08 (0.08-0.08)**	**0.03 (0.03-0.03)**	**0.06 (0.06-0.06)**
	13 days to ≤ 14 days	**0.02 (0.02-0.02)**	**0.08 (0.08-0.08)**	**0.03 (0.03-0.03)**	**0.06 (0.06-0.06)**
C12:1	0 to ≤ 1 day	0.03 (0.03-0.03)	0.20 (0.19-0.20)	0.03 (0.03-0.03)	0.20 (0.19-0.20)

	1 day to ≤ 3 days	**0.02 (0.02-0.02)**	**0.15 (0.15-0.15)**	**0.02 (0.02-0.02)**	**0.13 (0.13-0.13)**
	3 days to ≤ 5 days	0.02 (0.02-0.02)	0.11 (0.11-0.11)	0.02 (0.02-0.02)	0.11 (0.11-0.11)
	5 days to ≤ 6 days	0.01 (0.01-0.01)	0.08 (0.08-0.08)	0.01 (0.01-0.01)	0.08 (0.08-0.08)
	6 days to ≤ 7 days	**0.01 (0.01-0.01)**	**0.08 (0.08-0.08)**	**0.01 (0.01-0.01)**	**0.07 (0.07-0.07)**
	7 days to ≤ 10 days	0.01 (0.01-0.01)	0.07 (0.07-0.07)	0.01 (0.01-0.01)	0.07 (0.07-0.07)
	10 days to ≤ 11 days	0.01 (0.01-0.01)	0.07 (0.07-0.08)	0.01 (0.01-0.01)	0.07 (0.07-0.08)
	11 days to ≤ 14 days	0.01 (0.01-0.01)	0.06 (0.06-0.06)	0.01 (0.01-0.01)	0.06 (0.06-0.06)
C14	0 to ≤ 1 day	**0.10 (0.09-0.10)**	**0.31 (0.31-0.31)**	**0.10 (0.10-0.11)**	**0.29 (0.29-0.30)**
	1 day to ≤ 5 days	0.09 (0.09-0.09)	0.29 (0.29-0.29)	0.09 (0.09-0.09)	0.29 (0.29-0.29)
	5 days to ≤ 9 days	**0.07 (0.07-0.07)**	**0.26 (0.26-0.26)**	**0.06 (0.06-0.06)**	**0.23 (0.23-0.23)**
	9 days to ≤ 13 days	0.05 (0.05-0.05)	0.19 (0.19-0.19)	0.05 (0.05-0.05)	0.19 (0.19-0.19)
	13 days to ≤ 14 days	**0.05 (0.05-0.05)**	**0.17 (0.16-0.17)**	**0.04 (0.04-0.04)**	**0.15 (0.15-0.15)**
C14:1	0 to ≤ 1 day	**0.05 (0.05-0.05)**	**0.19 (0.19-0.20)**	**0.05 (0.05-0.05)**	**0.18 (0.17-0.18)**
	1 day to ≤ 2 days	0.04 (0.04-0.04)	0.19 (0.19-0.19)	0.04 (0.04-0.04)	0.19 (0.19-0.19)
	2 days to ≤ 3 days	**0.04 (0.04-0.04)**	**0.15 (0.15-0.15)**	**0.04 (0.04-0.04)**	**0.17 (0.17-0.17)**
	3 days to ≤ 4 days	0.04 (0.04-0.04)	0.14 (0.14-0.14)	0.04 (0.04-0.04)	0.14 (0.14-0.14)
	4 days to ≤ 5 days	0.03 (0.03-0.03)	0.10 (0.10-0.10)	0.03 (0.03-0.03)	0.10 (0.10-0.10)
	5 days to ≤ 6 days	0.03 (0.03-0.03)	0.09 (0.09-0.09)	0.03 (0.03-0.03)	0.09 (0.09-0.09)
	6 days to ≤ 7 days	**0.03 (0.03-0.03)**	**0.08 (0.08-0.08)**	**0.03 (0.03-0.03)**	**0.08 (0.08-0.08)**
	7 days to ≤ 10 days	**0.03 (0.02-0.03)**	**0.08 (0.08-0.08)**	**0.02 (0.02-0.02)**	**0.08 (0.08-0.08)**
	10 days to ≤ 11 days	**0.03 (0.03-0.03)**	**0.06 (0.06-0.06)**	**0.02 (0.02-0.02)**	**0.06 (0.06-0.06)**
	11 days to ≤ 12 days	**0.03 (0.03-0.03)**	**0.06 (0.06-0.06)**	**0.02 (0.02-0.02)**	**0.07 (0.07-0.07)**
	12 days to ≤ 13 days	**0.02 (0.02-0.02)**	**0.06 (0.06-0.06)**	**0.02 (0.02-0.02)**	**0.07 (0.07-0.07)**
	13 days to ≤ 14 days	0.02 (0.02-0.02)	0.07 (0.07-0.07)	0.02 (0.02-0.02)	0.07 (0.07-0.07)
C16	0 to ≤ 4 days	1.35 (1.35-1.35)	5.25 (5.25-5.25)	1.35 (1.35-1.35)	5.25 (5.25-5.25)
	4 days to ≤ 5 days	1.05 (1.04-1.05)	4.37 (4.36-4.38)	1.05 (1.04-1.05)	4.37 (4.36-4.38)
	5 days to ≤ 14 days	0.66 (0.66-0.66)	3.37 (3.37-3.38)	0.66 (0.66-0.66)	3.37 (3.37-3.38)
C16:1	0 to ≤ 1 day	0.07 (0.07-0.07)	0.35 (0.35-0.36)	0.07 (0.07-0.07)	0.35 (0.35-0.36)
	1 day to ≤ 3 days	0.07 (0.07-0.07)	0.34 (0.34-0.34)	0.07 (0.07-0.07)	0.34 (0.34-0.34)
	3 days to ≤ 4 days	0.06 (0.06-0.06)	0.31 (0.31-0.31)	0.06 (0.06-0.06)	0.31 (0.31-0.31)
	4 days to ≤ 5 days	**0.05 (0.05-0.05)**	**0.25 (0.25-0.25)**	**0.04 (0.04-0.04)**	**0.22 (0.22-0.22)**
	5 days to ≤ 6 days	**0.04 (0.04-0.04)**	**0.18 (0.18-0.18)**	**0.04 (0.04-0.04)**	**0.17 (0.17-0.17)**

	6 days to ≤ 7 days	0.03 (0.03-0.03)	0.15 (0.15-0.15)	0.03 (0.03-0.03)	0.15 (0.15-0.15)
	7 days to ≤ 8 days	**0.03 (0.03-0.03)**	**0.14 (0.14-0.14)**	**0.03 (0.03-0.03)**	**0.12 (0.12-0.12)**
	8 days to ≤ 9 days	0.03 (0.03-0.03)	0.11 (0.01-0.01)	0.03 (0.03-0.03)	0.11 (0.01-0.01)
	9 days to ≤ 10 days	**0.03 (0.03-0.03)**	**0.11 (0.01-0.01)**	**0.03 (0.03-0.03)**	**0.10 (0.10-0.10)**
	10 days to ≤ 11 days	**0.03 (0.03-0.03)**	**0.10 (0.10-0.10)**	**0.02 (0.02-0.02)**	**0.09 (0.09-0.09)**
	11 days to ≤ 13 days	0.02 (0.02-0.02)	0.08 (0.08-0.08)	0.02 (0.02-0.02)	0.08 (0.08-0.08)
	13 days to ≤ 14 days	**0.02 (0.02-0.02)**	**0.08 (0.08-0.08)**	**0.02 (0.02-0.02)**	**0.07 (0.07-0.07)**
C16:1-OH	0 to ≤ 1 day	0.02 (0.02-0.02)	0.05 (0.05-0.05)	0.02 (0.02-0.02)	0.05 (0.05-0.05)
	1 day to ≤ 2 days	**0.02 (0.02-0.02)**	**0.07 (0.07-0.07)**	**0.02 (0.02-0.02)**	**0.05 (0.05-0.05)**
	2 days to ≤ 4 days	0.02 (0.02-0.02)	0.05 (0.05-0.05)	0.02 (0.02-0.02)	0.05 (0.05-0.05)
	4 days to ≤ 6 days	**0.02 (0.02-0.02)**	**0.05 (0.05-0.05)**	**0.02 (0.02-0.02)**	**0.06 (0.06-0.06)**
	6 days to ≤ 7 days	**0.01 (0.01-0.01)**	**0.06 (0.06-0.06)**	**0.01 (0.01-0.01)**	**0.05 (0.05-0.06)**
	7 days to ≤ 8 days	**0.01 (0.01-0.01)**	**0.06 (0.06-0.06)**	**0.01 (0.01-0.01)**	**0.04 (0.04-0.04)**
	8 days to ≤ 14 days	0.01 (0.01-0.01)	0.04 (0.04-0.04)	0.01 (0.01-0.01)	0.04 (0.04-0.04)
C18	0 to ≤ 14 days	0.38 (0.38-0.38)	1.38 (1.38-1.39)	0.38 (0.38-0.38)	1.38 (1.38-1.39)
C18:1	0 to ≤ 14 days	0.73 (0.72-0.73)	2.25 (2.25-2.25)	0.73 (0.72-0.73)	2.25 (2.25-2.25)
C18:2	0 to ≤ 1 day	**0.07 (0.07-0.07)**	**0.36 (0.36-0.36)**	**0.06 (0.05-0.06)**	**0.34 (0.34-0.35)**
	1 day to ≤ 3 days	0.08 (0.08-0.08)	0.42 (0.42-0.42)	0.08 (0.08-0.08)	0.42 (0.42-0.42)
	3 days to ≤ 4 days	0.09 (0.09-0.09)	0.47 (0.47-0.47)	0.09 (0.09-0.09)	0.47 (0.47-0.47)
	4 days to ≤ 14 days	0.12 (0.12-0.12)	0.56 (0.01-0.01)	0.12 (0.12-0.12)	0.56 (0.01-0.01)

[a] Sex specific partitions are shown in bold. If no statistical difference was found between males and females within the age partitions, data were combined and RIs re-estimated.

[b] ALA, alanine; ARG, arginine; CIT, citrulline; GLY, glycine; LEU, leucine; ILE, isoleucine; ALLO-ILE, alloisoleucine; PRO-OH, hydroxyproline; MET, methionine; ORN, ornithine; PHE, phenylalanine; PRO, proline; TYR, Tyrosine; VAL, valine; C0, free carnitine; C2, acetylcarnitine; C3, propionylcarnitine; C3-DC+C4-OH, malonylcarnitine+3-hydroxybutyrylcarnitine; C4, butyrylcarnitine+isobutyrylcarnitine; C4-DC+C5-OH, methylmalonylcarnitine+3-hydroxyisovalerylcarnitine; C5, isovalerylcarnitine+methylbutyrylcarnitine; C5-DC+C6-OH, glutarylcarnitine+3-hydroxyhexanoylcarnitine; C6, hexanoylcarnitine; C6-DC, methylglutarylcarnitine; C8, octanoylcarnitine; C8:1, octenoylcarnitine; C10, decanoylcarnitine; C10:1, decenoylcarnitine; C12, dodecanoylcarnitine; C12:1, dodecenoylcarnitine; C14, tetradecanoylcarnitine; C14:1, tetradecenoylcarnitine; C16, palmitoylcarnitine; C16:1, palmitoleylcarnitine; C16:1-OH, 3-hydroxypalmitoleylcarnitine; C18, stearoylcarnitine; C18:1, oleoylcarnitine; C18:2, linoleoylcarnitine.

Table S12. Performance validation of the established RIs on true-positive (TP) cases

Conditions	Sampling age	Gender & No. of cases	Target biomarker	The disorder range for the target biomarker (μM)	The established RIs for the target biomarker (μM)	Whether the level of target biomarker in TP cases fell outside the established RIs
Hyperphenylalaninemia	0 to ≤ 1 day	Male (2)	PHE	>100.0	37.3-75.3	Yes
		Female (1)	PHE	>100.0	36.6-73.6	Yes
	1 day to ≤ 2 days	Male (4)	PHE	>100.0	34.7-74.2	Yes
		Female (8)	PHE	>100.0	34.7-74.2	Yes
	2 days to ≤ 3 days	Male (73)	PHE	>100.0	35.4-78.5	Yes
		Female (66)	PHE	>100.0	35.5-78.9	Yes
	3 days to ≤ 4 days	Male (59)	PHE	>100.0	34.8-78.0	Yes
		Female (46)	PHE	>100.0	34.9-78.2	Yes
	4 days to ≤ 5 days	Male (9)	PHE	>100.0	32.0-73.2	Yes
		Female (14)	PHE	>100.0	32.1-73.6	Yes
	5 days to ≤ 6 days	Male (10)	PHE	>100.0	31.1-72.7	Yes
		Female (7)	PHE	>100.0	31.4-72.6	Yes
	6 days to ≤ 7 days	Male (5)	PHE	>100.0	30.4-71.9	Yes
		Female (6)	PHE	>100.0	30.7-71.9	Yes
	7 days to ≤ 8 days	Male (4)	PHE	>100.0	30.0-71.5	Yes
		Female (3)	PHE	>100.0	30.1-72.3	Yes
	8 days to ≤ 9 days	Male (1)	PHE	>100.0	29.6-71.1	Yes
		Female (4)	PHE	>100.0	30.3-72.1	Yes
	9 days to ≤ 10 days	Male (2)	PHE	>100.0	29.6-70.9	Yes
		Female (1)	PHE	>100.0	30.1-72.0	Yes
	10 days to ≤ 11 days	Male (3)	PHE	>100.0	29.4-69.1	Yes
		Female (2)	PHE	>100.0	29.8-70.6	Yes
	11 days to ≤ 12 days	Male (0)	PHE	>100.0	29.3-68.6	\
		Female (1)	PHE	>100.0	30.0-69.5	Yes
	12 days to ≤ 13 days	Male (2)	PHE	>100.0	29.3-68.1	Yes
		Female (2)	PHE	>100.0	30.1-70.1	Yes
	13 days to ≤ 14 days	Male (0)	PHE	>100.0	29.2-67.7	\
		Female (2)	PHE	>100.0	30.3-68.7	Yes
Methylmalonic acidemia	0 to ≤ 1 day	Male (1)	C3	>4.50	0.79-2.82	Yes
		Female (1)	C3	>4.50	0.77-2.66	Yes
	1 day to ≤ 2 days	Male (1)	C3	>4.50	0.78-2.88	Yes
		Female (2)	C3	>4.50	0.74-2.75	Yes
	2 days to ≤ 3 days	Male (27)	C3	>4.50	0.77-2.95	Yes

		Female (13)	C3	>4.50	0.74-2.84	Yes
	3 days to ≤ 4 days	Male (18)	C3	>4.50	0.75-2.78	Yes
		Female (17)	C3	>4.50	0.72-2.70	Yes
	4 days to ≤ 5 days	Male (2)	C3	>4.50	0.66-2.44	Yes
		Female (3)	C3	>4.50	0.64-2.37	Yes
	5 days to ≤ 6 days	Male (0)	C3	>4.50	0.57-2.12	\
		Female (4)	C3	>4.50	0.55-2.09	Yes
	6 days to ≤ 7 days	Male (3)	C3	>4.50	0.49-1.88	Yes
		Female (1)	C3	>4.50	0.48-1.86	Yes
	7 days to ≤ 8 days	Male (2)	C3	>4.50	0.45-1.77	Yes
		Female (0)	C3	>4.50	0.43-1.75	\
	8 days to ≤ 9 days	Male (3)	C3	>4.50	0.41-1.68	Yes
		Female (3)	C3	>4.50	0.41-1.68	Yes
	9 days to ≤ 10 days	Male (2)	C3	>4.50	0.39-1.69	Yes
		Female (2)	C3	>4.50	0.39-1.69	Yes
	10 days to ≤ 11 days	Male (0)	C3	>4.50	0.38-1.66	\
		Female (2)	C3	>4.50	0.38-1.66	Yes
	12 days to ≤ 13 days	Male (0)	C3	>4.50	0.39-1.71	\
		Female (1)	C3	>4.50	0.39-1.71	Yes
	13 days to ≤ 14 days	Male (2)	C3	>4.50	0.39-1.74	Yes
		Female (0)	C3	>4.50	0.39-1.74	\
Propionic acidemia	2 days to ≤ 3 days	Male (8)	C3	>4.50	0.77-2.95	Yes
		Female (3)	C3	>4.50	0.74-2.84	Yes
	3 days to ≤ 4 days	Male (3)	C3	>4.50	0.75-2.78	Yes
		Female (2)	C3	>4.50	0.72-2.70	Yes
	5 days to ≤ 6 days	Male (0)	C3	>4.50	0.57-2.12	\
		Female (1)	C3	>4.50	0.55-2.09	Yes
	6 days to ≤ 7 days	Male (2)	C3	>4.50	0.49-1.88	Yes
		Female (0)	C3	>4.50	0.48-1.86	\
Short-chain acyl coenzyme A dehydrogenase deficiency	0 to ≤ 1 day	Male (1)	C4	>0.47	0.11-0.32	Yes
		Female (0)	C4	>0.47	0.12-0.33	\
	1 day to ≤ 2 days	Male (2)	C4	>0.47	0.07-0.32	Yes
		Female (1)	C4	>0.47	0.07-0.33	Yes
	2 days to ≤ 3 days	Male (27)	C4	>0.47	0.10-0.33	Yes
		Female (20)	C4	>0.47	0.11-0.36	Yes
	3 days to ≤ 4 days	Male (14)	C4	>0.47	0.11-0.32	Yes
		Female (10)	C4	>0.47	0.12-0.34	Yes

	4 days to ≤ 5 days	Male (3)	C4	>0.47	0.10-0.30	Yes
		Female (2)	C4	>0.47	0.11-0.31	Yes
	5 days to ≤ 6 days	Male (1)	C4	>0.47	0.10-0.28	Yes
		Female (1)	C4	>0.47	0.10-0.30	Yes
	6 days to ≤ 7 days	Male (2)	C4	>0.47	0.10-0.28	Yes
		Female (2)	C4	>0.47	0.10-0.29	Yes
	7 days to ≤ 8 days	Male (1)	C4	>0.47	0.09-0.27	Yes
		Female (0)	C4	>0.47	0.10-0.28	\
	11 days to ≤ 12 days	Male (1)	C4	>0.47	0.09-0.26	Yes
		Female (2)	C4	>0.47	0.09-0.27	Yes
	13 days to ≤ 14 days	Male (0)	C4	>0.47	0.09-0.26	\
		Female (2)	C4	>0.47	0.09-0.27	Yes
3-methylcrotonyl coenzyme A carboxylase deficiency	1 day to ≤ 2 days	Male (5)	C4-DC+C5-OH	>0.40	0.06-0.30	Yes
		Female (0)	C4-DC+C5-OH	>0.40	0.07-0.28	\
	2 days to ≤ 3 days	Male (15)	C4-DC+C5-OH	>0.40	0.10-0.33	Yes
		Female (21)	C4-DC+C5-OH	>0.40	0.10-0.30	Yes
	3 days to ≤ 4 days	Male (10)	C4-DC+C5-OH	>0.40	0.11-0.32	Yes
		Female (5)	C4-DC+C5-OH	>0.40	0.11-0.30	Yes
	4 days to ≤ 5 days	Male (2)	C4-DC+C5-OH	>0.40	0.11-0.30	Yes
		Female (0)	C4-DC+C5-OH	>0.40	0.10-0.28	\
	6 days to ≤ 7 days	Male (0)	C4-DC+C5-OH	>0.40	0.10-0.29	\
		Female (1)	C4-DC+C5-OH	>0.40	0.10-0.28	Yes
	8 days to ≤ 9 days	Male (1)	C4-DC+C5-OH	>0.40	0.10-0.28	Yes
		Female (2)	C4-DC+C5-OH	>0.40	0.10-0.27	Yes
	10 days to ≤ 11 days	Male (2)	C4-DC+C5-OH	>0.40	0.10-0.28	Yes
		Female (0)	C4-DC+C5-OH	>0.40	0.10-0.27	\
	11 days to ≤ 12 days	Male (0)	C4-DC+C5-OH	>0.40	0.10-0.28	\
		Female (1)	C4-DC+C5-OH	>0.40	0.10-0.27	Yes
	13 days to ≤ 14 days	Male (1)	C4-DC+C5-OH	>0.40	0.10-0.28	Yes
		Female (0)	C4-DC+C5-OH	>0.40	0.10-0.27	\
Citrin deficiency	1 day to ≤ 2 days	Male (1)	CIT	>35.0	7.5-20.3	Yes
		Female (0)	CIT	>35.0	7.6-20.7	\
	2 days to ≤ 3 days	Male (13)	CIT	>35.0	7.6-21.3	Yes
		Female (11)	CIT	>35.0	7.9-22.0	Yes
	3 days to ≤ 4 days	Male (4)	CIT	>35.0	7.8-21.2	Yes
		Female (4)	CIT	>35.0	8.1-22.1	Yes
	4 days to ≤ 5 days	Male (1)	CIT	>35.0	7.6-20.5	Yes

		Female (0)	CIT	>35.0	8.0-21.4	\
	5 days to ≤ 6 days	Male (1)	CIT	>35.0	7.7-20.6	Yes
		Female (1)	CIT	>35.0	8.0-21.4	Yes
	6 days to ≤ 7 days	Male (2)	CIT	>35.0	7.6-21.0	Yes
		Female (0)	CIT	>35.0	8.0-21.7	\
	7 days to ≤ 8 days	Male (1)	CIT	>35.0	7.6-21.7	Yes
		Female (0)	CIT	>35.0	7.9-22.4	\
	8 days to ≤ 9 days	Male (1)	CIT	>35.0	7.6-22.5	Yes
		Female (2)	CIT	>35.0	8.0-23.2	Yes
Hypermethioninemia	0 to ≤ 1 day	Male (2)	MET	>40.0	11.7-31.0	Yes
		Female (0)	MET	>40.0	11.7-31.0	\
	1 day to ≤ 2 days	Male (0)	MET	>40.0	10.5-31.0	\
		Female (2)	MET	>40.0	10.8-32.4	Yes
	2 days to ≤ 3 days	Male (8)	MET	>40.0	9.0-30.5	Yes
		Female (14)	MET	>40.0	9.5-31.8	Yes
	3 days to ≤ 4 days	Male (4)	MET	>40.0	9.0-31.3	Yes
		Female (10)	MET	>40.0	9.6-32.7	Yes
	4 days to ≤ 5 days	Male (4)	MET	>40.0	8.7-30.5	Yes
		Female (1)	MET	>40.0	9.2-31.8	Yes
	5 days to ≤ 6 days	Male (1)	MET	>40.0	8.5-29.9	Yes
		Female (0)	MET	>40.0	9.0-31.1	\
	6 days to ≤ 7 days	Male (1)	MET	>40.0	8.7-30.0	Yes
		Female (1)	MET	>40.0	9.0-30.8	Yes
	10 days to ≤ 11 days	Male (0)	MET	>40.0	9.5-33.3	\
		Female (2)	MET	>40.0	9.8-33.7	Yes
Maple syrup urine disease	2 days to ≤ 3 days	Male (3)	LEU/ILE/ALLO-ILE/PRO-OH	>500.0	92.0-229.4	Yes
		Female (1)	LEU/ILE/ALLO-ILE/PRO-OH	>500.0	94.5-234.7	Yes
	3 days to ≤ 4 days	Male (3)	LEU/ILE/ALLO-ILE/PRO-OH	>500.0	98.2-245.7	Yes
		Female (1)	LEU/ILE/ALLO-ILE/PRO-OH	>500.0	101.3-250.9	Yes
	8 days to ≤ 9 days	Male (1)	LEU/ILE/ALLO-ILE/PRO-OH	>500.0	100.4-277.5	Yes
		Female (0)	LEU/ILE/ALLO-ILE/PRO-OH	>500.0	103.6-284.4	\

	11 days to ≤ 12 days	Male (0)	LEU/ILE/ALLO-ILE/PRO-OH	>500.0	101.5-273.6	\
		Female (1)	LEU/ILE/ALLO-ILE/PRO-OH	>500.0	105.2-282.0	Yes
Very long-chain acyl coenzyme A dehydrogenase deficiency	1 day to ≤ 2 days	Male (0)	C14:1	>0.30	0.04-0.19	\
		Female (1)	C14:1	>0.30	0.04-0.18	Yes
	2 days to ≤ 3 days	Male (2)	C14:1	>0.30	0.04-0.15	Yes
		Female (5)	C14:1	>0.30	0.04-0.17	Yes
	3 days to ≤ 4 days	Male (3)	C14:1	>0.30	0.04-0.15	Yes
		Female (2)	C14:1	>0.30	0.03-0.13	Yes
	5 days to ≤ 6 days	Male (0)	C14:1	>0.30	0.03-0.09	\
		Female (1)	C14:1	>0.30	0.03-0.09	Yes
	7 days to ≤ 8 days	Male (0)	C14:1	>0.30	0.03-0.08	\
		Female (1)	C14:1	>0.30	0.02-0.08	Yes
	8 days to ≤ 9 days	Male (0)	C14:1	>0.30	0.02-0.08	\
		Female (1)	C14:1	>0.30	0.02-0.08	Yes
	9 days to ≤ 10 days	Male (1)	C14:1	>0.30	0.02-0.08	Yes
		Female (0)	C14:1	>0.30	0.02-0.06	\
	13 days to ≤ 14 days	Male (0)	C14:1	>0.30	0.02-0.07	\
		Female (1)	C14:1	>0.30	0.02-0.07	Yes
Isovaleric acidemia	0 to ≤ 1 day	Male (0)	C5	>0.30	0.04-0.15	\
		Female (1)	C5	>0.30	0.04-0.15	Yes
	1 day to ≤ 2 days	Male (1)	C5	>0.30	0.02-0.17	Yes
		Female (0)	C5	>0.30	0.02-0.18	\
	2 days to ≤ 3 days	Male (6)	C5	>0.30	0.05-0.17	Yes
		Female (0)	C5	>0.30	0.04-0.18	\
	3 days to ≤ 4 days	Male (2)	C5	>0.30	0.06-0.17	Yes
		Female (4)	C5	>0.30	0.06-0.18	Yes
	4 days to ≤ 5 days	Male (1)	C5	>0.30	0.06-0.18	Yes
		Female (0)	C5	>0.30	0.06-0.20	\
	6 days to ≤ 7 days	Male (0)	C5	>0.30	0.06-0.22	\
		Female (2)	C5	>0.30	0.06-0.23	Yes
	8 days to ≤ 9 days	Male (0)	C5	>0.30	0.06-0.23	\
		Female (1)	C5	>0.30	0.06-0.25	Yes
	12 days to ≤ 13 days	Male (1)	C5	>0.30	0.06-0.26	Yes
		Female (0)	C5	>0.30	0.06-0.26	\
Glutaric acidemia type I	0 to ≤ 1 day	Male (0)	C5-DC+C6-OH	>0.30	0.06-0.19	\

		Female (1)	C5-DC+C6-OH	>0.30	0.06-0.18	Yes
	1 day to ≤ 2 days	Male (0)	C5-DC+C6-OH	>0.30	0.06-0.25	\
		Female (6)	C5-DC+C6-OH	>0.30	0.06-0.24	Yes
	2 days to ≤ 3 days	Male (2)	C5-DC+C6-OH	>0.30	0.06-0.20	Yes
		Female (5)	C5-DC+C6-OH	>0.30	0.06-0.19	Yes
	3 days to ≤ 4 days	Male (2)	C5-DC+C6-OH	>0.30	0.06-0.18	Yes
		Female (2)	C5-DC+C6-OH	>0.30	0.05-0.18	Yes
	4 days to ≤ 5 days	Male (0)	C5-DC+C6-OH	>0.30	0.05-0.15	\
		Female (1)	C5-DC+C6-OH	>0.30	0.05-0.15	Yes
	5 days to ≤ 6 days	Male (1)	C5-DC+C6-OH	>0.30	0.05-0.14	Yes
		Female (0)	C5-DC+C6-OH	>0.30	0.05-0.13	\
	7 days to ≤ 8 days	Male (0)	C5-DC+C6-OH	>0.30	0.04-0.14	\
		Female (1)	C5-DC+C6-OH	>0.30	0.04-0.14	Yes
	8 days to ≤ 9 days	Male (1)	C5-DC+C6-OH	>0.30	0.04-0.12	Yes
		Female (0)	C5-DC+C6-OH	>0.30	0.04-0.12	\
	10 days to ≤ 11 days	Male (1)	C5-DC+C6-OH	>0.30	0.04-0.12	Yes
		Female (0)	C5-DC+C6-OH	>0.30	0.04-0.12	\
Carnitine palmitoyltransferase I deficiency	0 to ≤ 1 day	Male (0)	C0	>55.00	10.44-33.08	\
		Female (1)	C0	>55.00	10.14-29.97	Yes
	2 days to ≤ 3 days	Male (2)	C0	>55.00	11.30-37.73	Yes
		Female (0)	C0	>55.00	10.65-34.69	\
	3 days to ≤ 4 days	Male (0)	C0	>55.00	11.88-38.59	\
		Female (1)	C0	>55.00	11.09-35.64	Yes
	4 days to ≤ 5 days	Male (1)	C0	>55.00	11.86-38.23	Yes
		Female (0)	C0	>55.00	11.05-35.71	\
	6 days to ≤ 7 days	Male (0)	C0	>55.00	11.76-38.68	\
		Female (1)	C0	>55.00	11.22-36.42	Yes
	7 days to ≤ 8 days	Male (0)	C0	>55.00	11.78-39.12	\
		Female (1)	C0	>55.00	11.28-37.26	Yes
Argininemia	2 days to ≤ 3 days	Male (2)	ARG	>80.0	1.5-25.4	Yes
		Female (0)	ARG	>80.0	1.5-23.8	\
Tyrosinemia	2 days to ≤ 3 days	Male (1)	TYR	>240.0	49.7-176.6	Yes
		Female (1)	TYR	>240.0	50.7-166.0	Yes
Hyperprolinemia	2 days to ≤ 3 days	Male (2)	PRO	>440.3	120.3-294.2	Yes
		Female (0)	PRO	>440.3	122.3-295.2	\
	3 days to ≤ 4 days	Male (8)	PRO	>440.3	125.3-310.4	Yes
		Female (5)	PRO	>440.3	127.6-312.6	Yes

	4 days to ≤ 5 days	Male (12)	PRO	>440.3	119.7-310.7	Yes
		Female (15)	PRO	>440.3	122.1-314.3	Yes
	6 days to ≤ 7 days	Male (6)	PRO	>440.3	117.0-316.1	Yes
		Female (5)	PRO	>440.3	121.7-319.1	Yes
	7 days to ≤ 8 days	Male (5)	PRO	>440.3	117.4-313.8	Yes
		Female (2)	PRO	>440.3	120.7-316.0	Yes
	10 days to ≤ 11 days	Male (2)	PRO	>440.3	116.7-302.1	Yes
		Female (1)	PRO	>440.3	120.5-305.7	Yes

Abbreviations: ARG, arginine; CIT, citrulline; MET, methionine; PHE, phenylalanine; TYR, Tyrosine; C0, free carnitine; C3, propionylcarnitine; C4, butyrylcarnitine+isobutyrylcarnitine; C4-DC+C5-OH, methylmalonylcarnitine+3-hydroxyisovalerylcarnitine; C5, isovalerylcarnitine+methylbutyrylcarnitine; C5-DC+C6-OH, glutarylcarnitine+3-hydroxyhexanoylcarnitine; C6, hexanoylcarnitine; C8, octanoylcarnitine; C14:1, tetradecenoylcarnitine.

Note: "/" means not validated.

Table S13. The information of additional co-authors

Name of co-authors	Affiliation
Yuxia Zhou	The Maternal and Child Health Care Hospital of Shandong Province, Jinan, P. R. China
Kai Mu	Z. B. Maternity and Child Health Care Hospital, Zibo, P. R. China
Qiaoling Sun	Anhui Woman and Child Health Care Hospital, Hefei, P. R. China
Xinmei Mao	The Maternity and Child Health Care Hospital of Ningxia Hui Autonomous Region, Yinchuan, P. R. China
Jun He	Changsha Hospital for Maternal and Child Health Care, Changsha, P. R. China
Hua Tang	Hunan Provincial Maternal and Child Health Care Hospital, Changsha, P. R. China
Juying Pei	Hebei Maternity and Child Health Care Hospital, Shijiazhuang, P. R. China
Dehua Zhao	NO. 3 Affiliated Hospital of Zhengzhou University, Zhengzhou, P. R. China
Yanmin Wang	Department of Newborn Screening Center, Shanghai Children's Hospital, Shanghai Jiao Tong University, P. R. China
Rong Qiang	The Affiliated Northwest Women and Children’s Hospital of Xi'an Jiao Tong University, Xi'an, P. R. China
Junkun Chen	Ganzhou Maternity and Child Health Care Hospital, Ganzhou, P. R. China
Jianping Yang	The Maternity and Child Health Care Hospital of Shanxi Province, Taiyuan, P. R. China
Wenbin Zhu	Fujian Maternity and Child Health Hospital, Fuzhou, P. R. China
Lixin Ye	Dongguan City Maternal and Child Health Hospital, Dongguan, P. R. China
Xin Fan	The Maternal and Child Health Hospital of Guangxi Zhuang Autonomous Region, Nanning, P. R. China
Mingcai Ou	Sichuan Provincial Hospital for Women and Children, Chengdu, P. R. China
Wei Wei	Changzhi Maternity and Child Health Care Hospital, Changzhi, P. R. China
Yulin Zhou	The Women and Children’s Hospital, School of Medicine, Xiamen University, Xiamen, P. R. China
Ying Gu	Lianyungang Maternity and Child Health Care Hospital, Lianyungang, P. R. China
Wei Wen	Shenzhen Children’s Hospital, Shenzhen, P. R. China
Limin Wang	Women and Infants Hospital of Zhengzhou, Zhengzhou, P. R. China
Ying Liu	Jilin Maternity and Child Health Care Hospital, Changchun, P. R. China
Shengju Hao	Gansu Provincial Maternity and Child-care Hospital, Lanzhou, P. R. China
Honghong Zhang	Guiyang Maternity and Child Health Care Hospital, Guiyang, P. R. China
Yuhong Wang	Hubei Maternity and Child Health Care Hospital, Wuhan, P. R. China
Liliang Wan	Huanggang Maternity and Child Health Care Hospital, Huanggang, P. R. China
Yuanyuan Kong	Beijing Maternity and Child Health Care Hospital, Beijing, P. R. China
Jie Wang	Hainan Women and Children's Medical Center, Haikou, P. R. China
Hong Chang	Shenyang Maternity and Child Health Hospital, Shenyang, P. R. China
Ying Chan	The First People's Hospital of Yunnan Province, Kunming, P. R. China
Jing Liu	Bozhou Maternity and Child Health Family Planning Service Center, Bozhou, P. R. China
Peng Xie	Chuzhou Maternity and Child Health Family Planning Service Center, Chuzhou, P. R. China
Long Li	The People's Hospital of Xinjiang Uygur Autonomous Region, Urumqi, P. R. China

Figure S1. The dynamic change of methionine over age and sex.

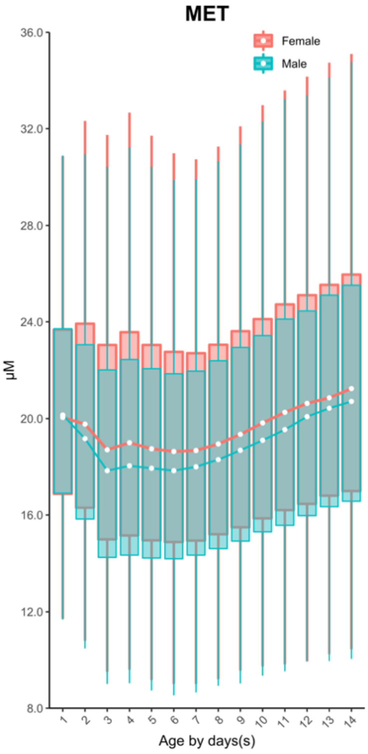

The data of male and female partitions are shown in green and red boxes with whiskers, respectively. The boxes extend from the 25th to the 75th percentile, with whiskers extending to the 2.5th or 97.5th percentile. The medians are shown as white circles in the body of the boxes, and are linked with green (male) or red (female) line to shown the dynamic trends over age. MET, methionine.

Figure S2. The dynamic change of C4-DC+C5-OH, C8, C8:1, C10:1, C16:1-OH and C18:1 over age and sex.

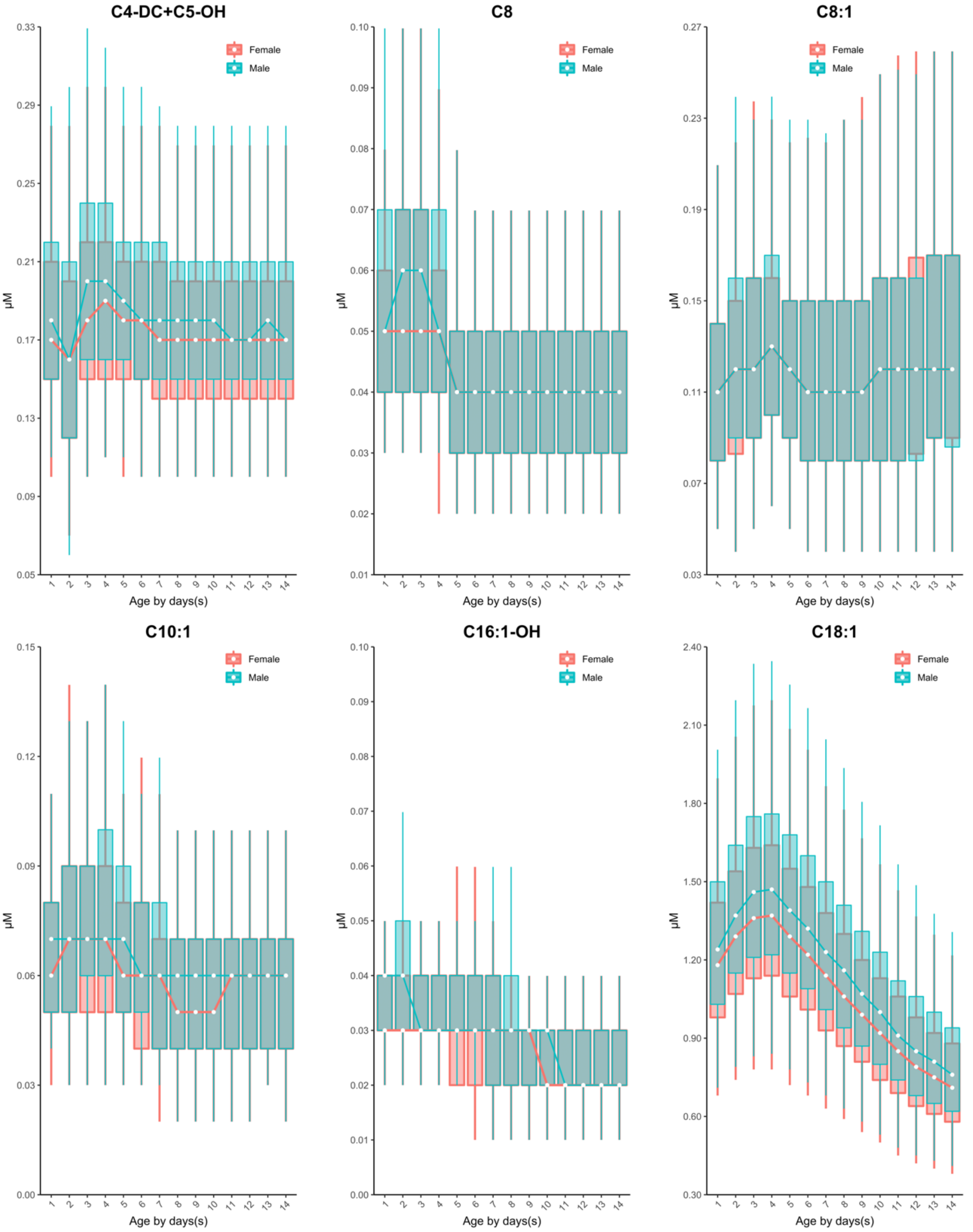

The data of male and female partitions are shown in green and red boxes with whiskers, respectively. The boxes extend from the 25th to the 75th percentile, with whiskers extending to the 2.5th or 97.5th percentile. The medians are shown as white circles in the body of the boxes, and are linked with green (male) or red (female) line to shown the dynamic trends over age. Abbreviations are listed in the legend of Table 1.
